# A Review of the Use of GPEs in Zinc-Based Batteries. A Step Closer to Wearable Electronic Gadgets and Smart Textiles

**DOI:** 10.3390/polym12122812

**Published:** 2020-11-27

**Authors:** Sebastián Lorca, Florencio Santos, Antonio J. Fernández Romero

**Affiliations:** Grupo de Materiales Avanzados para la Producción y Almacenamiento de Energía (MAPA), Campus de Alfonso XIII, Universidad Politécnica de Cartagena, Cartagena, 30203 Murcia, Spain; sebastian.lorca.ro@gmail.com

**Keywords:** GPEs, gel polymer, zinc-air battery, biobased GPE, wearable devices, PVA, PAM, PAA

## Abstract

With the flourish of flexible and wearable electronics gadgets, the need for flexible power sources has become essential. The growth of this increasingly diverse range of devices boosted the necessity to develop materials for such flexible power sources such as secondary batteries, fuel cells, supercapacitors, sensors, dye-sensitized solar cells, etc. In that context, comprehensives studies on flexible conversion and energy storage devices have been released for other technologies such Li-ion standing out the importance of the research done lately in GPEs (gel polymer electrolytes) for energy conversion and storage. However, flexible zinc batteries have not received the attention they deserve within the flexible batteries field, which are destined to be one of the high rank players in the wearable devices future market. This review presents an extensive overview of the most notable or prominent gel polymeric materials, including biobased polymers, and zinc chemistries as well as its practical or functional implementation in flexible wearable devices. The ultimate aim is to highlight zinc-based batteries as power sources to fill a segment of the world flexible batteries future market.

## 1. Introduction

Zinc presents a promising alternative as battery material given its relatively high abundance, low toxicity, higher safety parameters, and low potential, making it appropriate for use in energy storage systems [1]. There are convincing reasons to use zinc as battery material: this metal has a considerable negative standard potential of −0.76 V, it can be produced in high purity, and subsequently can be alloyed to get stable formulations in aqueous electrolytes for long times. It has a low price and around the world there exists a long tradition in zinc recycling. Batteries with zinc are inherently safe, even in case of destruction of puncture of the battery casing, there is no fire hazard associated with that.

The world demand for refined zinc was around 14 Million metric tons (Mt) according to the International Metals Study Groups [2]. There is an estimated 2.800 million Mt of zinc contained in the Earth’s crust in such form and amount, that economic extraction is currently or potentially feasible with 480 million Mt of reserve base; consequently, zinc is a non-critical metal. Besides, zinc recycling contribute significantly to sustainability, which is already an important share of the worldwide used zinc [3], reducing concentrate demand, energy use, emissions and minimizes waste disposal. As a comparison, lithium reserves account only for 17 million Mt [4] and none of the current battery recycling technologies for lithium are perfect and need to be adjusted based on composition of active materials [5]. Sodium and potassium suffer from both high reactivity as Lithium and their production process, as intensive energy consuming process, chlorine-alkali sector, are affected, for instance, in EU by Emissions Trading System [6]. Besides the use of aprotic non-aqueous complex electrolytes can hinder their development. 

Battery systems based on multivalent metals such as calcium (Ca) have attracted much interest to become the next-generation energy storage systems, but they are in a very early stage of development trying to obtain a practical electrolyte for efficient Ca deposition [7]. Production of Magnesium is also intensive energy consuming and it is capable of reducing water, like sodium and potassium, and releasing highly flammable hydrogen gas. It reacts also with ambient carbon dioxide releasing heat. Iron shows low voltage and aluminum batteries relies on the mobility of AlCl_4_^−^ instead of Al^3+^ due to its low mobility in many electrolytes [8].

The above-mentioned details set a framework where the family of zinc-based batteries (Ni-Zn, Ag-Zn, Zn-MnO_2_, Zn-ion, Zn-air, and Zn-Bi_2_O_3_) offer a compelling alternative and trustworthy operation and safety in aqueous alkaline electrolytes [9], with practical specific energies ranging from 80 to 475 Whkg^−1^, Table 1.

**Figure 1:**
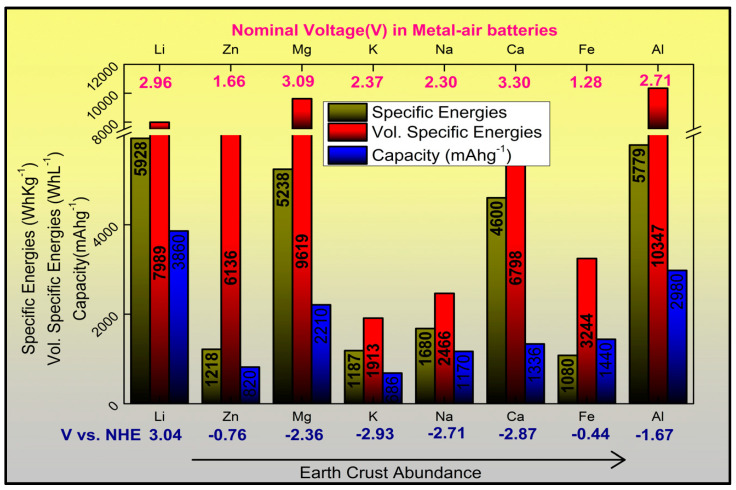
Theoretical gravimetric and volumetric energies, oxygen included, capacities, standard reduction potential vs. normal hydrogen electrode (NHE) and nominal voltages in metal-air batteries of metal negative electrodes used in electrochemical storage systems as well as their earth crust abundance. Adapted from reference [10].

Primary and secondary metal–air batteries, with metals such as zinc, aluminum, iron, lithium, potassium, sodium, and magnesium attracted much attention due to their theoretical specific energies. Gravimetric energy densities, volumetric energy densities, and nominal cell voltages of various metal anodes in metal–air batteries are shown in Figure 1.

A special case is the Zn-air system with a theoretical energy density of 1350 Whkg^−1^, which is about 5 times higher than Li-ion batteries and so far is the only metal-air battery commercially available. Just recently, Ecomarinepower, in co-operation with Furukawa Battery Co., Ltd., Fukuoka, Japan, introduced an emergency magnesium-air battery. Recently our group carried out a LCA (Life Cycle Analysis) analysis of a lab scale battery where the levelized cost of energy obtained revealed that this zinc technology could become competitive if cyclability is improved: a calculation for 2000 cycles delivered a capital cost for energy storage around 100 $/MWh/cycles, better than most of current technologies [11].

With the flourishing of flexible and wearable electronics gadgets, their flexibility has become essential [12,13]. The growth of this increasingly diverse range of devices boosted the necessity to develop materials for the conversion and storage of energy devices such as secondary batteries, fuel cells, supercapacitors, sensors, dye-sensitized solar cells (DSSC), etc. that supply energy to these wearable electronics. IDC (International Data Corporation) expects worldwide IoT spending will maintain a double-digit annual growth rate throughout the 2017–2022 forecast period and surpass the $1 trillion mark in 2022 [14]. Data-driven customized services enabled by wearable electronics will be another key aspect for this growth according to EU commission [15]. It can be said, flexible batteries is now at the spotlight as reflected by the number of papers published in recent years [16]. The race has begun, in the market there are already several manufacturers offering their flexible batteries [17,18], even custom-shaped batteries [19].

When it comes to flexible batteries, it should undoubtedly be necessary to bring up the manufacturing process to the front page. Printed batteries are based on the development of special inks for each battery component, whereas thin-film batteries are typically deposited using physical vapor deposition, typically by thermal evaporation and sputtering in vacuum. In this methodology, patterning is normally accomplished indirectly masking or substrate dicing, but both types of batteries hold the desirable flexible property in order to be used in flexible and wearable electronics gadgets. The main commercially available printed batteries are non-rechargeable batteries based on zinc-manganese dioxide (Zn/MnO_2_) with ZnCl_2_ as an electrolyte. Different companies offer printed batteries and it is also possible to obtain customized batteries with different application requirements [28,29,30].

Electrolytes are fundamental components in power sources. Safety is of paramount importance when dealing with electronic devices in which will be used, for instance, in a continuous monitoring thermometer to check the temperature of a baby or glucose sensors [31]. Traditional liquid electrolytes suffer major drawbacks, which sometimes become unaffordable or impossible to overcome as far as these devices are concerned. Liquid electrolytes have always played an essential role in electrochemical energy storage mainly due to their high ionic conductivities (10^−3^−10^−2^ Scm^−1^) and good contact with electrodes [32]; however, the use of liquid electrolytes entail risks such leakages and even combustion of organic electrolytes which render them unsuitable for this kind of devices. Others drawbacks of liquid electrolytes are: dendrite growth in liquid solution, which is caused by inhomogeneities that produce preferential nucleation and uneven currents when charging [33] (which may also cause fire [34]), evaporation, handling issues, etc. Therefore, the use of gel polymer electrolytes would be the more suitable electrolytes for such batteries because they encompass both benefits; a leak-free design and high conductivity and reaction kinetics [13].

The study of polymer electrolytes commenced with Wright et al. [35] in 1973, but their technological significance was not noticed until the research undertaken by Armand et al. [36] demonstrated that the semi crystalline phase formed from alkali metal salts and polyethylene oxide (PEO) were capable of with significant ionic conductivity, and pointed out their possible application as battery electrolytes. Since then, great progress in the field of polymer electrolytes has been made and a big number of technical papers and reviews have been produced [32,37,38,39,40,41,42,43,44].

**Figure 2:**
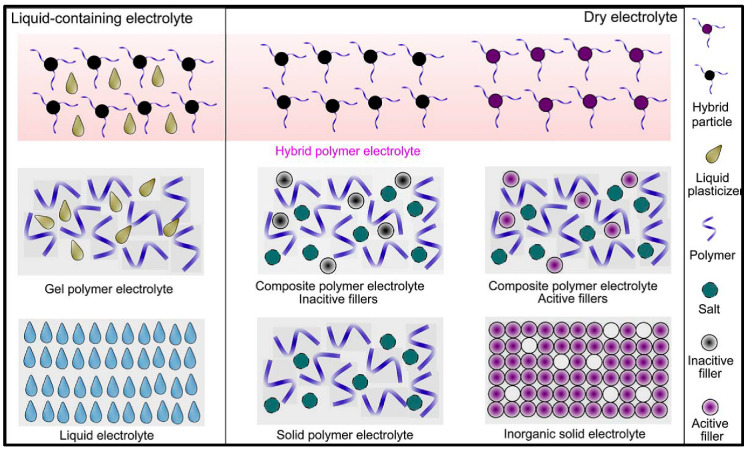
Classification of electrolytes for batteries: Reproduced with permission from [45]. Copyright 2011 Elsevier.

Polymer electrolytes (Figure 2) are classically classified in dry solid polymer electrolyte (S-PE), plasticized polymer electrolytes (P-PE), gel polymer electrolytes (G-PE), Ionic rubber polymer electrolytes (IR-PE) and Ion-conducting polyelectrolytes (IC-P). Inorganic species that introduced into the former types, generally oxide nanopowders, give rise to the well-known composite polymer electrolytes (C-PE). A more comprehensive and detailed classification of this kind of materials are out of the scope of this chapter but can be found in the extensive report done by Di Noto et al. [44].

Solid polymer electrolytes (SPE) are unsuitable to be used in power sources due their low ionic conductivities (10^−8^−10^−5^ Scm^−1^) and poor interfaces with electrodes [32,46,47]. One of the most common approaches to further optimize polymer-salt electrolytes conductivity is the introduction of ionic liquids into the polymer-salt system [48]. Gel polymer electrolytes (GPE) also known as plasticized PE, was first introduced by Feuillade and Perche in 1975 [49]. Gel polymer electrolytes (GPEs) are materials, which are neither solids nor liquids, but hold both the cohesive properties of solids and the diffusive character of liquids. Hence, these electrolytes became relevant due to their use as excellent substitutes of the liquid electrolytes or as separator in batteries [39]. GPEs have higher conductivity at room temperature than SPEs, approximately 10^−2^ Scm^−1^; however present a safety concern in the long-term operation due to the content of solvent, but it is also true that this concern is lower than using liquid electrolyte. GPEs can act as a separator to reduce Zn dendrite formation and increase Zn use as well as, in case of zinc-air batteries with alkaline electrolyte, prevent the electrolyte from penetrating the air electrode where it can form carbonates in alkaline electrolytes blocking the oxygen access to the actives sites [10].

In addition, it is necessary to mention hydrogels as water containing gel polymer electrolytes. Aqueous-based GPEs or hydrogels are three-dimensional (3D) network of hydrophilic polymers that can swell in water and hold a large amount of water while maintaining the structure due to chemical or physical cross-linking of individual polymer chains [50]. This type of GPE can be used as electrolyte [51] in batteries and supercapacitor and can be considered emerging materials for energy storage [52]. Hydrogels are also promising materials as bioelectronics interfaces [53]. The development of new electrolytes to replace the existing traditional synthetic polymer electrolyte, is also explored as to obtain polymer electrolytes from natural and renewable resources. Among natural polymers, polysaccharides and proteins are best candidates due to their abundance in the environment [54,55]. There is real feverish research activity in screening any kind of natural biobased product that could be used as electrolytes in electrochemical devices [56,57,58,59,60,61,62,63,64,65,66,67,68], without mentioning they can also be used as additives or binders.

The uncontrollable growth of zinc dendrites and the dissolution of Zn in alkaline electrolytes during the charge-discharge process result in detrimental effects in its performance. The use of gel polymer electrolyte (GPE), apart from reasonable flexibility, stability and mechanical strength and high ionic conductivity (10^−4^–10^−3^ S/cm) offers a good protective effect toward zinc metal electrode. In aqueous electrolyte, all the reactions on zinc metal involve a strong interaction between Zn and water molecules, the generation of a hydroxyl ion (OH^−^) and protons (H^+^) via water dissociation often drives the formation of hydrogen gas, Zn(OH)_2_, or zincates [23]. One way to avoid this problem may be similar to the concept of high concentration salt in liquid electrolytes, the amount of free water in GPE, in case of aqueous GPEs, is significantly reduced compared to liquids such as aqueous electrolyte and hence, the problem that zinc metal would have in normal aqueous electrolyte is effectively suppressed. However, an important mayor drawback about GPEs is the difficulty in matching the polymer component and the electrolyte salt to avoid the salting-out or salt precipitation of the commonly used water-dispersible polymers [64]. Likewise, syneresis is another problem where GPE undergo severe exudation of liquid electrolytes from porous gel polymer structure upon long storage [69].

The chemistry of solid electrolyte interfaces (SEI) is one of the critical factors that govern the cycling life of rechargeable batteries. An ideal SEI stabilizes electrodes, avoids corrosion, and hinders the dendrite formation, which are vital for improving the cell performance. This electron-insulating interface chokes or impedes direct electron supply between the electrode to electrolyte molecules, and subsequently stops or hinders the unwanted reactions and minimizes the degradation of the electrolyte and electrodes materials, all of them, factors that undermine discharge-charge efficiency. These layers were termed as the “solid–electrolyte interface (SEI)” by Peled in 1979 [70]. The solid electrolyte interface (SEI) formed on the surface of Li electrode, for instance, in Li metal batteries is considered to be favorable for enhancing the cycling ability of the Li electrode as a protective layer. Therefore, approaches were developed to improve the stability and uniformity of SEI, or build an artificial SEI to stabilize both Li or zinc electrodes [71,72]. 

Zn deposition is inevitably interfered with by competitive H_2_ evolution and insulating reaction products, such as Zn hydroxides and zincates, especially for aqueous alkaline electrolytes that passivates the fresh Zn electrode. In a long-running operation, such irreversible reactions proliferate at the electrolyte-Zn interface, lessening cyclability. Thus, the viable aqueous Zn metallic anodes are enabled only if both requisites of dendrite-free deposition and side-reaction suppression are fulfilled. Homogeneous SEI prevented localized Zn metal nucleation and growth, thus eliminating the Zn dendrite formation, furthermore, accommodated the interface fluctuation during repeated charging/discharging without breakdown. More importantly, SEI facilitated uniform transport of ions throughout the whole electrode surface.

The GPEs themselves, on the other hand, form a protective coating on the zinc metal [73] which was also demonstrated extensively for other electrode materials [73,74,75]. This protective feature assimilated to GPEs have been also revealed by Zhao et al. [76] in a liquid aqueous Zn/MnO_2_ battery. According to the authors, the ideal protective interphase should allow ionic conduction but forbidding electronic conduction, the polymer coating should be dense, hydrophilic but water-insoluble and strong but at the same time also flexible to accommodate the volumetric change during electrochemical cycling. Besides, the polymer chains are required to be rich with polar groups for interacting with solvated metal ions. They engineered a multifunctional polymeric interphase of polyamide (PA) and ZnTf_2_ (zinc triflate), what in the paper is denominated a “brightener”, with unique hydrogen-bonding network and the ability to strongly coordinate with metal ions. Dendrite-free and stable Zn deposition combined with an effective suppression of side reactions, the Zn/Zn^2+^ cycling demonstrates an ultralong lifespan up to 8000 h at a low current density of 0.5 mAcm^−2^ though. Moreover, this polymer-engineered Zn anode supports the operation of an aqueous Zn/MnO_2_ battery to deliver a capacity retention of 88% after 1000 cycles with a Coulombic efficiency (CE) above 99%.

Resuming, polymer gel electrolyte with large ion migration channels, cheap, reasonable mechanical properties, and high affinity are desired for batteries with high performance to be used in wearable devices. The main objective of this chapter is to gather and review all the relevant or innovative work done on zinc batteries lately using gel polymer electrolytes. The task ahead is enormous; hence the work has been constrained to the most important or widespread gel polymer electrolytes focusing ourselves in Zinc-MnO_2_ and Zinc-air batteries, but we have not disregarded any other representative zinc chemistry or cutting edge work that could provide a valuable perspective to the readers. This detailed discussion is organized from the perspective of the gel polymer electrolyte followed by cathode materials, considering the anode the less crucial component of a zinc-based battery for several reasons; air cathodes are still the performance-limiting electrode of Zn–air batteries [77], the availability of the Zinc makes it a cheap element which are normally in excess within the batteries, extensive research about zinc anodes is available in the literature [78,79,80,81,82,83] and Coulombic efficiency (CE) may be considered high enough, reaching in some cases up to 90% and higher, hence with little perspective of improvement [84]. Cyclability is a common denominator for all electrochemically rechargeable secondary batteries, not only for zinc-based batteries. In some excellent works with complex fully nanostructured cathodes, batteries are tested with plain bare Zn foil [12,64,85,86,87,88], which led us to not consider Zn anodes as relevant in this work. Thus, future zinc-based batteries research should be more directed toward incorporating the best performing Zn anodes to enable long-term stable and efficient battery cycling [78] as well as enabling easier benchmarking. 

Comprehensive studies on flexible conversion and storage energy devices were recently released for other technologies standing out the importance of the research done lately in the GPEs for energy conversion and storage research field [89,90,91,92,93]. This work is intended to resemble to zinc batteries what the outstanding and extensive work done by Mauger et al. [92] devoted to Lithium, although the main title may suggest other technologies were also included. In others recent extensive reviews [43,89,90,94] of non-lithium-based flexible batteries, zinc batteries were treated scarcely or not thoroughly. Just very recently, Tilahun Tsehaye et al. published a review of membranes only for zinc-air batteries [95]. Likewise, Chen et al., in a section of a recent review, have gone through flexible zinc-air batteries using GPEs [96]. According to Tilahun and co-workers, membranes can be categorized in seven types, GPEs being one of them, which are called ion solvating membranes. Tsehave also devoted another work to AEM in metal-air batteries [97]. Thus, this work aims to fill the gap we believe exists specifically in zinc batteries when solid electrolyte is used.

## 2. Chemistries of Zinc-Based Batteries

This section will be an abbreviated one as it is not one of the main objectives of this work and more detailed information about zinc-based batteries chemistries can be found for more classical Zn-Ag, Zn-Ni and Zinc-air batteries [98,99], and for more recent Zn-Ion ones [16,100].

Nevertheless, basic description about zinc-ion mechanisms, with MnO_2_ only, will be given due to the relevance acquired lately. The energy storage mechanism of the ZIBs is mainly the conversion of the cathode accompanied by the ion insertion/extraction into and from the host materials, similar to the “rocking-chair” model proposed by Armand in the late 70th [101]. Many different cathode materials are used as hosts to accommodate Zn^2+^ ions: Manganese Oxide Cathode Materials, Vanadium-Based Cathode Materials, Prussian Blue Materials and others MoS_2_, graphite, ChevrelPhase, etc. [102].

There are six polymorphs of manganese dioxide: α-MnO_2_ (2 × 2 tunnel or hollandite), β-MnO_2_ (1 × 1 tunnel or pyrolusite), R-MnO_2_ (2 × 1 tunnel or Ramsdellite), γ-MnO_2_ (mix of 2 × 1 and 1 × 1 tunnels or nsutite), δ-MnO_2_ (layered or birnessite), and λ-MnO_2_ (3-dimensional pores or spinel); being λ-MnO_2,_ known as electrolytic manganese dioxide, the predominant polymorph in commercial alkaline batteries [103]. The polymorphs have distinctive atomic arrangements that result in various types of pores or tunnels within the crystal structure. In Manganese-based cathodes for ZIBs, three energy storage mechanisms have mainly been demonstrated: Zn^2+^ intercalation-deintercalation, H^+^/Zn^2+^ co-insertion, and reversible H^+^ redox conversion reaction. 

Generally, the intercalation/extraction of Zn^2+^ ions take place repeatedly during the charge/discharge course in the host materials with layers and tunnels, such as α-MnO_2_, δ-MnO_2_, and γ-MnO_2_. δ-MnO_2_ is usually viewed as superior candidate for cathode material of ZIBs among various analogs since its large interspace can decrease electrostatic interactions between the framework and Zn^2+^, leading to a facilitated cation diffusion in the host structure [104]. Spinel ZnMn_2_O_4_ is the intermediate formed after Zn^2+^ insertion, which is converted back to MnO_2_ during the extraction of Zn^2+^ (sometimes not completely). Spinel-type structures are not an ideal choice for the insertion of Zn^2+^ due to the limited three-dimensional (3D) tunnels and strong electrostatic repulsion between Zn-ion and the lattice. The equations in both electrodes are shown below with α-MnO_2_ as an example:

Cathode: Zn^2+^ + 2e^−^+ 2MnO_2_ ↔ ZnMn_2_O_4_

Anode: Zn ↔ Zn^2+^ + 2e^−^

In neutral mildly acidic environment (ZnSO_4_ + MnSO_4_), it is probable that Zn^2+^ and H^+^ to be inserted into the Mn-host. This mechanism has been relatively soon revealed in Zn/MnO_2_ batteries with aqueous electrolytes [105]. The successive H^+^ and Zn^2+^ insertion processes proceed with difference in reaction kinetics during the discharging and was revealed by two discharge plateaus in the galvanostatic discharge curves at 1.4 and 1.3 V respectively, being the first plateau related to the H^+^ insertion due to its smaller size, whereas the second plateau is mainly attributed to the Zn^2+^ insertion [105].

Redox conversion reversible mechanism is observed in α-MnO_2_ in mild aqueous ZnSO_4_ [106]. During the charge/discharge process, the cathode experiences the chemical transformation between MnOOH and α-MnO_2_ following:

Cathode: H_2_O ↔ H^+^ + OH^−^

MnO_2_ + H ^+^ + e^−^↔ MnOOH

12Zn2+ +OH− + 16ZnSO4 + x6H2O↔16 ZnSO4[Zn(OH2)]3·xH2O

Anode:12Zn↔12Zn2+ + e−

**Figure 3:**
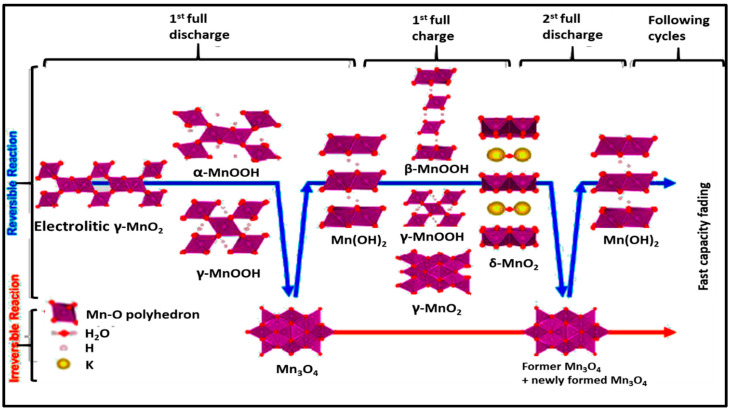
Reaction mechanism of γ-MnO_2_ cathode in a standard alkaline battery. Reproduced with permission from [24]. Copyright 2016 the American Chemical Society.

γ-MnO_2_ (electrolytic manganese) in rechargeable alkaline Zn/MnO_2_ experiences also the redox conversion of γ-MnO_2_ to MnOOH in a first step, then it will be reduced to Mn_3_O_4_ or Mn(OH)_2_ depending on the depth of discharge (DOD) or the cut-off voltage, Figure 3. If only 1.33 electrons per MnO_2_ were drawn out, the end product would be Mn_3_O_4_. However, if two electrons are completely drawn out from the MnO_2_, the final product would be Mn(OH)_2_. When electrolytic manganese dioxide is recharged, the process is reversed [98]. The irreversibility of MnO_2_ is strongly correlated with the formation of the spinel phases Mn_3_O_4_ and ZnMn_2_O_4_ [24]. The overall reaction, depending on the DOD, in alkaline Zn-MnO_2_ batteries are as follows:

2Zn + 3MnO_2_ → 2ZnO+ Mn_3_O_4_

Zn + MnO_2_ + 2H_2_O → ZnO + Mn(OH)_2_ + 2OH^−^

Although considerable progress has been made, the electrochemical reaction mechanisms involving the MnO_2_ cathodes for ZIBs is still a topic of controversy. Among these mechanisms, reversible Zn^2+^ insertion/extraction apparently is the main responsible for the high capacity of ZIBs [104].

## 3. The Polymer Matrix of the GPEs

The preparation of GPEs requires at least one host polymer acting as base material prior to the addition of any other component. Examples of host polymer that are commonly used in the preparation of GPEs are poly(vinyl chloride) (PVC), poly(vinyl alcohol) (PVA), poly(acrylic acid) (PAA), PEO, poly(acrylonitrile) (PAN), poly(vinylidene fluoride) (PVdF), poly(ethyl methacrylate) (PEMA), poly(methyl methacrylate) (PMMA), poly(vinylidenefluoride-hexafluoropropylene) (PVdF-HFP), biopolymers, etc. [43]. Better GPEs properties were obtained using structurally similar polymers or copolymers [107,108,109,110,111]. As a result, GPEs with better electrochemical and mechanical stability as well as better flexibility has been produced. Table 2 gathers in a list, which is not exclusive, the most popular polymer used for making GPEs. 

Typically, a polymeric framework is used in GPEs as host material, providing mechanical integrity. Several criteria that polymer must fulfill to be considered to be good hosts are [112]:

- fast segmental motion of polymer chain.
- functional groups promoting the dissolution of salts.
- low glass transition temperature (*T*_g_).
- high molecular weight.
- wide electrochemical window.
- high degradation temperature.

The properties of the GPEs components should confer the right properties to the GPEs in order to fulfill three basic requirement in respect of performance, durability and cost [44]. In general, the “performance level” is defined in terms of the ionic conductivity of the material, which should be as high as possible. Its durability or reliability must be enough to withstand the normal conditions of operation in the long term. Cost should be affordable in order to allow the devices in which GPEs will be used to become a mass market good. More specifically, GPEs must have:

- Good Ionic Conductivity: In GPEs, the ionic conductivity is strongly related to the degree of crystallinity, porosity, and the ability of the polymer to uptake solvent in the matrix [113]. Since ionic conductivity is directly proportional to the concentration of charged species and their electrical mobility, as per σ = n_i_*q_i_*µ_i_, it is expected that ionic conductivity to be lower in GPEs as the mobility of ions is higher in liquid electrolytes [114].
- High Cationic Transference Number (t_+_): Besides the ionic conductivity, the transference number of polymer electrolytes is an important figure of merit when assessing their efficacy. The cationic transference number is the cation mobility relative to the anion in single salts GPEs. In GPEs, working species need to have a transference number close to unity [114].
- Good Chemical and Thermal Stability. For a gel polymer electrolyte to be effective, it needs to maintain high ionic conductivity over a wide temperature range and remain structurally stable during manufacturing, cell assembly, storage, and usage [69]. Introducing inorganic particles such as TiO_2_, SiO_2_ or Al_2_O_3_ is an strategy to improve the thermal stability of GPEs [32,115,116]. Another strategy is to create a composite of several polymers so that could enhance the thermal stability of GPEs [117].
- Good Mechanical Properties. Since a GPE is “sandwiched” between the cathode and the anode, it should be mechanically stable and not undergo deformation or strain that could jeopardize the stability of the battery itself. At the same time, they must be able to withstand volume change; however, gel polymer electrolytes most of the time show poor mechanical properties due to solvent or plasticizer used trying to increase conductivity. Therefore, there must be a trade-off between ionic conductivity and mechanical endurance.
- Low cost. GPEs with excellent characteristic only would be wearable for commercial application if it can be produced at low cost. It makes necessary an easy fabrication process. We have to keep in mind that the process of developing any new energy storage device must follow the idea underlying in Barnhardt and Benson’s quote for stationary energy storage batteries, “if a battery’s energetic cost is too high, its overall contribution to global warming could negate the environmental benefits of the wind or solar farm it was supposed to support”, but applied to this flexible GPEs as component of wearable electronics gadgets [118].

Ion-conducting polymers are formed dissolving low lattice dissociations energy salts in a polar polymer matrix. The polymer electrolytes, being mixed phase (crystalline and amorphous) together to charge carriers, are an example of the so-called disordered material. Hence, these materials are dealt with theoretical models applicable to other disordered materials [119]. Within the polymer network, the salts in the GPEs serve as the source of the charge carriers, which are generally required to have low dissociation energy in order to dissociate in the free mobile ions [32] that will be responsible for the conductivity. The mobility of ions in the GPE determines the conductivity σ and its performance. Plasticizers, such as ethylene carbonate (EC), propylene carbonate (PC), dimethyl carbonate (DMC), etc. are usually added to the polymer electrolyte to improve the electrical conductivity of PE by enhancing polymer chains flexibility as well as the stability of electrode/electrolyte interface [120,121,122,123,124,125]. 

The main macroscopic theories to study ionic transport, encompass the empirical conductivity vs. Temperature model such Arrhenius and Vogel-Tamman-Fulcher (VTF), free volume-based models and configurational entropy model. For dry solid polymers, as PEO alkaline metal complex electrolytes, inter and intra chain hoping mechanism should be responsible for the ionic transport due to the stiffness and immobile polymers chains. This blend shows no linear behavior of Arrhenius plots below its glass transition temperature. More recently, works with PEO_6:_LiXF_6_ (X = P, As, Sb) [126] showed two folded polymer chains to form cylindrical tunnels, in which the cations are located inside and coordinated by the functional groups of the polymers whereas the anion is located outside these tunnels and do not coordinate the cations and the conduction mechanism seem to be fully decoupled from the segmental relaxation which is a key for the development of superionic polymer electrolytes [127]. For gel polymer electrolytes must the ionic conductivity due to combination of ion/polymer cooperative motion with the occasional independent ion movement due to the presence of more amorphous region. As gel polymer electrolytes hold a certain amount of liquid electrolyte, sometimes in vast quantities with respect to the polymer matrix weight, the ability of the polymer matrix to absorb the liquid electrolyte depend on the cohesive energy of the polymer matrix and therefore of the chain interactions, free volume which depend of the chain movements, glass transition temperature, crosslinking degree, nature of the liquid electrolyte and temperature [128]. 

The incorporated liquid electrolyte forms a network whereby the ion conduction takes place along with the host polymer. The connected network of amorphous regions will eventually reach the The amorphous regions grow larger in number and size in GPE, owing to the absorption of liquid. percolation threshold and fast ion-conducting pathways are developed, which acts upon enhancing the ion mobility and hence the higher ionic conductivity [130]. 

As we have already mentioned, the performance level of a GPE is mainly assessed by its conductivity value. Therefore, to conclude this section, a comprehensive table with the most relevant GPEs presented in this review is included as reference guide including Ionic conductivity, mechanical strength as well as the type of battery where the GPEs is included in Table 3.

### 3.1. Poly(Vinylidene Fluoride-Co-Hexafluoropropylene) (PVdF-HFP)

Polyvinylidene fluoride (PVdF) is an industrial thermoplastic of the fluoropolymer family, semicrystalline with three crystalline phases (α, β and γ), the first two prevail at room temperature, being the latter responsible for the piezoelectric characteristics in the PVdF, due to the orientation of the atoms in its molecular structure [131]. PVdF shows piezoelectricity which allows it to be used interestingly as Piezofiber in Smart Textiles [132,133]. 

PVdF has a simple melting process because of its relatively low melting point of around 177 °C. It has low density; its molecular structure and high crystallinity give rise to a high mechanical stability, even at high temperatures. Its excellent resistance to chemical products makes it an ideal material for a great number of applications, generally in applications that require high purity, strength and resistance to solvents, acids, bases, heat generation and low smoke emission during an event of fire. 

In particular, PVdF with strong electro-withdrawing functional groups (–C–F) shows a high dielectric constant (ε = 8.4), which is beneficial for the dissolution of salts to keep a high concentration of charge carriers within the matrix fostering this way conductivity [134]. Thus, copolymerization of VdF and HFP, to give PVdF-HFP lowers crystallinity compared to PVdF. Therefore, PVdF-HFP contains more amorphous domains capable of holding large amounts of salts and liquid electrolytes [135]. 

It is considered also that the increase of amorphous domains within a polymer matrix provides better conductivity since the ions are more freely to move. Hence, PVdF-HFP is regarded as a promising matrix as gel polymer electrolyte material. Main properties of PVdF-HFP properties can be found in [136].

Many articles were devoted, firstly, to study and characterize PVdF-HFP GPEs, but not being the fabricated GPEs used in any prototype battery. Tsunemi and Tsuchida [137] noted that the most critical problem of the PVdF-based polymer electrolyte is its incompatibility toward Lithium metal. In fact, fluorinated polymers are not chemically stable toward Lithium, which results in the formation of LiF and renders PVdF-based electrolytes unsuitable for Lithium metal batteries. Xu et al. [138] reported in 2005 a novel zinc-ion conducting polymer gel electrolytes based on non-volatile RT ionic liquids (ILs). The GPE consists of an ionic liquid, with a zinc salt dissolved in PVdF-HFP. The resultant electrolyte membranes are freestanding, translucent, flexible and elastic, with excellent mechanical integrity and strength. Conductivity values ranged from 10^−4^ Scm^−1^ at −20 °C to 4–5·10^−3^ Scm^−1^ at 80 °C. Electrochemical tests show that zinc ions are mobile in the membranes and zinc metal is capable of dissolution into and deposition from the membranes.

Likewise, Tafur et al. [195] studied GPEs with different ILs and PVdF-HFP, with and without ZnTf_2_ salt. The incorporation of IL (ionic liquid) or IL + ZnTf_2_ salt to the polymer produces more amorphous and polar membranes compared to the pristine PVdF-HFP film, as it was deduced from ATR-FTIR and XRD spectroscopies. Ionic conductivity, impedance and voltammetry measurements indicated that the electrical properties of the GPEs clearly depend on the IL cation used. From impedance results, a P_6,6,6,14_ TFSI electrolyte proved to be more resistive than EMIMTFSI and EPyTFSI-based electrolytes, which can be attributed to the major volume of the P_6,6,6,14_ cation. The inclusion of ZnTf_2_ salt inside the ionic liquid-based GPE produced an increase in the ionic conductivity and a decrease in activation energy, which is related to the existence of free triflate anions, as it was deduced from ATR-FTIR analyses. In addition, the observation of Zn^2+^ movement inside these membranes provides the possibility to use these new GPEs in Zn-based batteries. Same authors studied also the influence of the IL type in the performance of the gel polymer electrolytes in Zn-based batteries. The changes observed occurred only when no ZnTf_2_ salt was added to the gel or the added quantity was too low. For higher concentration the salt addition masked the differences making Zn^2+^ the most important charge carrier in all GPEs regardless of the IL used [139].

The interest in PVDF-HFP is not decaying. Proof of that is the “fresh from the oven” in depth study conducted by Liu et al. [196] on the dielectric properties and Zn^2+^ ionic conduction mechanism involved in the PVdF-HFP/Zn(Tf)_2_ complex system. They found, using dielectric data that the optimal mass ratio in respect of conductivity values was achieved at 0.4 (Zn(Tf)_2_:PVdF-HFP with a conductivity value of 2.44∙10^–5^ Scm^−1^ and a ion transference number of 0.98. They also demonstrated that these types of GPEs hinder the formation of dendrites comparing the same battery in liquid and in GPE electrolytes.

The use of nanofillers have been also explored for PVDF-HFP GPEs. Johnsi et al. [140,197] reported nanocomposite SPEs based on PVdF-HFP and Zn (CF_3_SO_3_)_2_ (Zn/Tf_2)_ containing different inorganic fillers (i.e., TiO_2_, ZrO_2_, and CeO_2_). Three composite materials showed similar features: the maximum ion conductivity and amorphous ratio could be achieved for the samples with ~5 wt.% nanomaterials (5 wt.% for TiO_2_ and CeO_2_, 7 wt.% for ZrO_2_). It was also found that the GPEs with ZrO_2_ exhibited the best ion conductivity performance (0.46 mScm^−1^ at 25 °C). Sellan and Hasmi [174] found a substantial enhancement in Zn^2+^ ion transport from 0.35 to 0.55 due to the dispersion of ZnO nanoparticles in a blend of 1.0 M solution of zinc triflate [Zn(Tf)_2_] in ethylene carbonate (EC)–propylene carbonate (PC) immobilized in PVdF-HFP.

Especially interesting would be Zn–air batteries based on non-aqueous polymer electrolytes like PVDF-HFP. They would offer a main advantage over current Zn–air batteries using aqueous alkaline electrolytes to avoid evaporation, but this polymer is not suitable to be used in alkaline media due to dehydrofluorination [101]; however, it has been used profusely in Zn-MnO_2_ and Zinc-ion batteries.

#### 3.1.1. Zn-MnO_2_

Batteries using GPEs can also be manufactured by using a state-of-the art or fashionable way through ink printing. Thus, Ho and co-workers [198] developed a printed microbattery with zinc and manganese dioxide electrodes sandwiching an ionic liquid gel electrolyte using direct dispenser printing. The stacked type battery, 0.25 cm^2^, delivered 0.98 mA hm^−2^ over more than 70 cycles. The test cells had a thickness between 80 and 120 μm. Inks for the dispenser were composed of: electrode 95 wt.% zinc powder and 5 wt.% PVDF-HFP for the zinc electrode and 90 wt.% activated MnO_2_ powder, 6 wt.% acetylene black and 4 wt.% PVDF-HFP for MnO_2_ electrodes. The gel electrolyte is composed of a 1:1 mixture of PVDF-HFP and 0.5 M solution of zinc trifluoromethanesulfonate Zn(Tf)_2_ salt dissolved in BMIMTf ionic liquid and was determined to have optimal mechanical integrity and transport properties. At this composition, the room temperature gel ionic conductivity was 0.37 mScm^−1^. Same group using roll-to-roll flexographic printing [199] managed to print cathodes which were tested in batteries using the same GPE composition. The charge–discharge cycles can be repeated up to 55 cycles with minimum loss of capacity. Also, Tafur et al. [200] investigated the charge storage mechanism of zinc cation in a Zn/MnO_2_ battery using GPEs made of PVdF-HFP, BMINTf as ionic liquid, and ZnTf_2_ as salt. They observed by EDX the presence of Zn along the whole depth of the discharged cathode. Thus, they could confirm that Zn^2+^ cations are intercalated in the whole cathode, besides, not all Zn^2+^ cations are pulled out during the recharge. With the addition of ionic liquid to PVDF-HFP-based electrolyte, it is observed that the interaction between PVdF chains and zinc-ion becomes weaker, enhancing the ion mobility.

#### 3.1.2. Zn-Ion

First example of the use of manganese oxide for zinc-ion batteries was provide by Kumar and Sampath [201] in one of the first studies also of PVdF-HFP GPE applied to zinc batteries. The authors stated that an electrochemical equilibrium between zinc metal and the Zn^2+^ ions in the GPE is reached, which is of primary concern from any application using GPE in Zn batteries. Zn//GPE//MnO_2_ cells were assembled and analyzed for their discharge capacity and cycling life data. Liu et al. [202] reported the synthesis of a GPE ionic liquid-based, 1-butyl-1- methylpyrrolidinium trifluoromethylsulfonate, [Py1,4]Tf, containing 0.2 M ZnTf_2_, immobilized in a PVdF-HFP copolymer. Kim et al. [203] investigated the effects of layer-by-layer printing, using also PVdF-HFP, in the performance of a Zn//GPE//MnO_2_ battery stencil printed. Fully printed cells showed significant improvements in discharge capacity in large part due to the increased interfacial cohesion between layers by direct layer-by-layer printing.

The very active group of Longtao Ma [141] belonging to Department of Materials Science and Engineering, City University of Hong Kong, developed a 1-ethyl-3-methylimidazolium tetrafluoroborate (EMIMBF_4_) ionic liquid-based zinc-ion battery with; zinc tetrafluoroborate (Zn(BF_4_)_2_) as supporting electrolyte salt (ILZE), PVDF-HFP-PEO (5 wt. % PEO) as gel electrolyte (PHP-ILZE) and cobalt hexacyanoferrate (CoHCF) as cathode. The PHP-ILZE gel achieves a very low density of 10.15 mgcm^−2^, which is lower than 10% that of polyacrylamide (PAM), poly(acrylic acid) (PAA), sodium polyacrylate (PANa), and poly(vinyl alcohol) (PVA) hydrogels, which are include in the study for comparison purposes. Furthermore, PHP-ILZE gel, allegedly, can supply higher energy density in zinc-ion batteries. The manufactured battery Zn//PHP-ILZE//CoHCF behaviors similarly to the liquid one counterpart. The system delivers a specific capacity of 149.53 mAhg^−1^ at 0.2 Ag^−1^. Furthermore, after 30,000 cycles at 2 Ag^−1^ the system still exhibits a high coulombic efficiency of ≈100% and a specific capacity of 93.1 mAhg^−1^, which is 90% of the initial capacity. Such stable cycle performance is attributed to the electrochemical stability of PHP-ILZE electrolyte and excellent interface stability among different components.

One of the most remarkable features of the PHP-ILZE GPE is the suppression of both HER and corrosion of Zn. The more positive corrosion potential and lower corrosion current represent less tendency of corrosion reaction and low corrosion rate, respectively. Thus, compared with the corrosion potential of Zn in 2 M ZnSO_4_ (−0.967 V) and 2 M Zn(BF_4_)_2_ (−0.908 V) aqueous electrolytes, the Zn in PHP-ILZE GPE shows a much more positive corrosion potential of −0.579 V. The corrosion rate is also lower for PHP-ILZE GPE. Certainly, the PHP-ILZE GPE creates a highly stable environment for metallic Zn electrodes. 

### 3.2. Polyvinil Alcohol (PVA)

PVA is a carbon chain backbone polymer with hydroxyl groups attached to the methylene. Despite sharing the same constitutional unit formula (−C_2_H_4_O−) with PEO, polyvinyl alcohol PVA is more versatile due to their OH pendant groups susceptible to physical crosslinking via H-bonding or chemical esterification. It is highly hydrophilic, non-toxic and exhibits good thermal and chemical stability. It has excellent film-forming properties [204], possess astonishing optical properties, large dielectric strength, and excellent charge storage ability [205]. Its mechanical, optical, and electrical attributes can be tailored by doping with nanofillers. PVA is available on the market in different grades on viscosity and degree of hydrolysis basis.

Besides that, PVA has hydroxyl groups that produce inter and intra-molecular hydrogen-bonding. PVA is biocompatible, non-toxic and exhibits minimal cell adhesion and protein absorption. It is used in many application of relevance even used in the biomedical field as scaffolding for tissue engineering where the cell growing will take place [206].

Much work has been done with PVA as GPE for batteries different of the ones dealt with in this work highlighting the usefulness of PVA and PVA blends as GPE in batteries and not only for zinc batteries [207,208,209,210,211,212,213]. On the other hand, there are also numerous papers devoted to blend PVA with biopolymers [64,214,215].

PVA has been also modified by UV radiation with various amounts of 2-hydroxy ethyl methacrylate (HEMA) monomer, resulting PVA/HEMA copolymers. Besides, SiO_2_ additive was incorporated in different proportions to this blend obtaining PVA/HEMA–SiO_2_ [216]. Next, both of the prepared blends were soaked in 40 wt.% KOH solution to form the GPEs. Copolymerization of PVA and HEMA provided decreasing of the crystalline structure with respect to the pure PVA, and thus increasing the amorphous regions, as it was demonstrated by several experimental techniques. This aspect also confirms that HEMA acts as a crosslinking agent. Besides, the inclusion of SiO_2_ to the copolymer resulted in interactions between the OH^−^ groups of PVA and/or HEMA molecules causing an additional decrease of the gel crystallinity with the SiO_2_ amount. In addition, incorporation of SiO_2_ particles with ≡SiOH groups into the membrane provides a superior absorption of water due to the formation of H-bonds, resulting a swelling ratio at about 152%, whereas a maximum value of 117% was obtained for PVA/HEMA. Finally, the ionic conductivity increased from 0.044 (PVA) to 0.073 Scm^−1^ for the (PVA/HEMA) membrane, whereas it was about 0.11 Scm^−1^ for the PVA/HEMA–SiO_2_ membranes, at room temperature.

#### 3.2.1. Zn-MnO_2_

Among the various energy storage devices, alkaline zinc-manganese dioxide (Zn-MnO_2_) batteries have long dominated the battery market for most kind of applications needing a low cost, high safety, easy manufacturing and high energy density power sources [217].

This battery technology is also used to create fiber-shaped batteries that could be used in smart textiles or E-textiles. In this sense Wang et al. [218] developed a flexible solid-state rechargeable Zn-MnO_2_ metal microbattery based on a MnO_2_ electrodeposited on CNT (carbon nanotubes) as cathode and a zinc wire as anode using a PVA/LiCl/ZnCl_2_ gel electrolyte. The specific capacity of Zn-MnO_2_ cable micro-battery at 0.1 Ag^−1^ with ZnCl_2_ gel polymer electrolyte was 290 mAhg^−1^, almost the same capacity obtained when liquid electrolyte was used.

**Figure 4:**
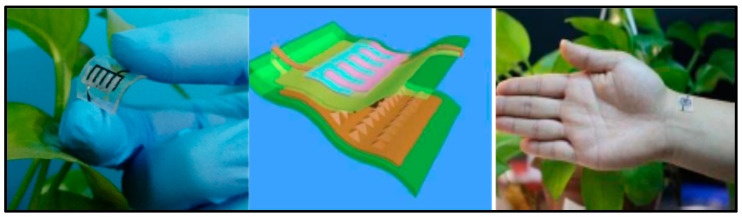
Optical photographs of a microbattery-pressure sensor system for pulse detection. Schematic diagram of the microbattery-pressure sensor system (center). Reproduced with permission from [219]. Copyright 2018 Royal Society of Chemistry.

He et al. [219] used highly conductive three-dimensional (3D) Ni skeleton for current collection in order to improve battery capacity. Thus, the interdigitated CNTF@Zn//GPE//MnO_2_@Ni@CNTF battery, using a GPE with PVA and LiCl, ZnCl_2_, and MnSO_4_ as ionic species, powered an integrated pressure sensor. The pressure sensor was constructed using two SWCNTs–PDMS bilayers (SPP) with the micro-patterned surfaces touching each other and the microbattery was assembled on the other side of the PDMS, Figure 4. Pressure-sensing tests were performed; sensitivity, response time, stability, and reproducibility of the sensor were confirmed. The battery delivered 0.718 mAhcm^−2^ and a remarkable energy density of 0.98 mWhcm^−2^.

Zeng et al. [142] covered the cathode with a layer of PEDOT:PSS (poly(3,4-ethylenedioxythiophene):polystyrene sulfonate). According to the authors this protective layer enhanced the initial capacity retention along cycling at a discharge current of 1.11 Ag^−1^, the Zn–MnO_2_@PEDOT battery delivered a reversible specific capacity of ≈310 mAh^−1^, and recovered to ≈280 mAhg^−1^ after 300 cycles. As a proof-of-concept, a flexible quasi-solid- state Zn–MnO_2_@PEDOT battery with a voltage of 1.8 V was also assembled with PVA/ZnCl_2_/MnSO_4_ gel as electrolyte. This quasi-solid-state Zn//GPE//MnO_2_@PEDOT battery achieved a capacity of 282.4 mAhg^−1^ and considering the total mass of the cell, it resulted in a gravimetric energy density of 34 WhKg^−1^. This two last works have developed their GPEs using PVA with ZnCl_2_, and MnSO_4_, but both of then failed to provide comparative conductivity or mechanical strength values.

#### 3.2.2. Zn-Air

Power sources that are textile-based are thought to be a key component for wearable electronic devices, still in the research and development stage. The advantage of “smart textiles” [220,221] is its flexibility and ability to be integrated into clothing. The uniform of the dismounted warfighter is an example of a military application for electronic devices and power sources textile-based [222]. Power sources that are Zinc-air-based will be open to the surrounding air, due to their semiopen structure. It is important to study the influence of the environment on the electrolyte and the corresponding battery performance. Recently Fan et al. [143] accomplished this study for the most common used polymer electrolyte, namely PVA-KOH, in zinc-air batteries including its dimensional stability and water and ionic conductivity retention capability in ambient air. With the increase in the exposure time in ambient air, the PVA–KOH GPE suffered from an obvious shape change and severe loss of water and ionic conductivity, which resulted in degradation of the cycling property, discharge performance and power output of the fabricated FZABs (Fuel Zinc-Air battery). Most extended testing period of time was used by Santos et al. [51] to check water content and conductivity of soaked in 12 M KOH PVA-KOH GPEs.

Furthermore, Li et al. [144] use a novel polymer electrolyte based on quaternary ammonium hydroxides, TEAOH–PVA, instead of the typical PVA-KOH, flexible zinc–air batteries. The electrolyte exhibits excellent discharge performance and cycling life of ZABs using TEAOH−PVA electrolyte and zinc foil (0.3 mm thick, 1 × 2 cm^2^) was investigated by galvanostatic charge−discharge (GCD) measurements compared to that based on the commonly used KOH–PVA electrolyte, and no notable degradation is observed after two weeks. The presence of water in the polymer electrolyte plays a crucial role in determining the performance of ZABs. High water content in GPEs facilitates the conduction of OH^−^ and could determine the reaction kinetics of batteries. The loss of water reduces the water molecules required for the OH^−^ transport in the electrolyte which leads to a large decrease in ionic conductivity and performance degradation of the electrolyte [51]. Similarly, Qu et al. [223] performed the same study but using porous Zn anode with 3D foam architecture via electroplating Zn on Cu foam substrate instead, reaching same conclusions. Both studies contained Co_3_O_4_ as catalyst in the air electrodes.

Currently, the main bottleneck of ZABs (Zinc-air batteries) still lies in the kinetically sluggish rate of Oxygen reduction reaction (ORR) on the cathode side, and determines the energy conversion efficiency and output power density, but some progress has been made lately though. The use of Co_3_O_4_ as catalyst in air electrodes, some other catalysts are also explored, in flexible batteries PVA-based has been fruitful. The most common feature is the use of carbon cloths as substrate, not exclusive, where the catalyst is applied using diverse techniques and with different nanostructured morphologies [224,225,226]. The following works describe flexibles zinc-air batteries, with different geometries, where huge effort have been put in developing air electrodes with enhanced capabilities.

With the aforementioned issues in mind, Li et al. [145] used a 1D knittable Zinc–Air battery. The battery, a flexible yarn-shaped ZAB with a controllable length, fabricated by a quick, facile, and continuous method, subsequently, can be knitted into clothes and textiles for wearable electronics. The bifunctional catalyst of mesoporous Co_3_O_4_/nitrogen-doped reduced graphene oxide (N-rGO) hybrid nanosheets, revealed itself as the key parameter for the good performance of the battery. The hybrid nanosheets exhibited uniform layer-by-layer morphology consisting of a top Co-based mesoporous layer and an N-rGO substrate with an averaged thickness of 1.8 nm as ascertain by AFM. Zinc wire (0.25 mm) was wrapped in chiffon and soaked continuously in a PVA solution (0.15 M KOH), subsequently, this was coated with carbon fibers that previously were sprayed with the catalysis ink. The authors managed to charge iPhone 4S cellphone with three ZAB sets in parallel knitted (each set contains three in series ZABs). Similarly, Guan et al. [227] created fiber-shaped Zn-air battery, ~0.3 cm diameter that could be also knitted provided the diameter is reduced but using a PAA-based GPE.

Yu et al. [228] introduced a novel additive-free design to fabricate highly active air-cathode for flexible ZABs avoiding time-consuming operations, but also allowing also large-scale preparation. Mesoporous Co_3_O_4_ nanowire arrays on carbon cloth as cathodes were used in flexible zinc-air batteries, CC/Zn//GPE//N-Co_3_O_4_/CC achieving 2.5 mAcm^−3^ exhibited a volumetric capacity of 98.1 mAhcm^−3^. PVA grafted with a KOH base was used as gel polymer electrolyte.

**Figure 5:**
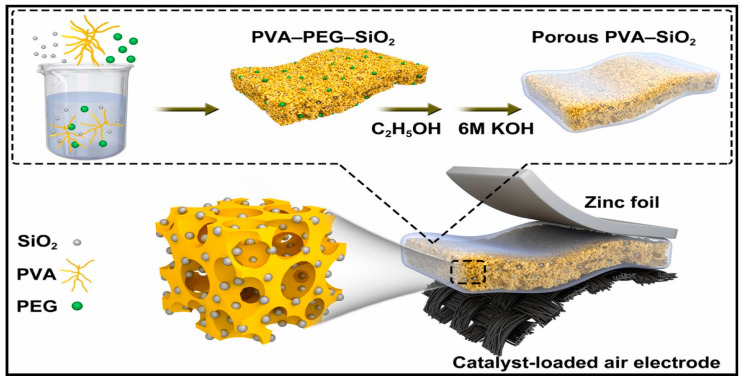
Schematic diagram of the flexible ZAB and preparation process of the porous PVA-based nanocomposite GPE along with its inner structure. Reproduced with permission from [146]. Copyright 2019 Elsevier.

Chen et al. [229] used, also as bifunctional catalyst, Co_3_O_4_ grown on the surface of carbon fibers trying to overcome the intrinsically poor electrical conductivity of Co_3_O_4_. Inspired by its excellent flexibility and strong adhesion of the ultrathin Co_3_O_4_ layers on the carbon cloth electrode, a flexible display unit, composed of an electroluminescent device, fabricated on a flexible PET with ITO, (Cu-doped ZnS) as active layer and BaTiO3 as dielectric layer, was attached to the flexible zinc-air battery, Cu/Zn//PVA-KOH//Co_3_O_4_/CF to become an integrated device able to work under bending, twisting, etc. conditions. Fan et al. [146] used also Co_3_O_4_ on carbon cloth as cathode but in sandwiched type battery using zinc foils as anode. The nanoporous GPE structure created in the PVA network by adding PEG, which was removed later on soaking the gel in acetone, outperformed the conventional PVA–KOH GPE (Figure 5). The obtained porous PVA-based nanocomposite GPE containing 5 wt.% SiO_2_ exhibited the highest ionic conductivity (57.3 mScm^–1^), but did not state what type of SiO_2_ was used. The discharge gravimetric capacity and energy density of ZAB using the porous PVA–5 wt.% SiO_2_ GPE of 720.6 mAhgZn^–1^ was obtained.

Fu et al. [147] showed the goodness of prefabricated sheets of customized shapes and sizes to fit them into batteries for a wide variety of applications. A porous, crosslinked with glutaraldehyde polymer electrolyte membrane was prepared by a phase inversion technique and then soaked in a solution of KOH/PVA (35/2 wt.%). The membrane resulted in being freestanding with a conductivity of 0.015 Scm^−1^. A bifunctional air electrode with core–corona structured LaNiO_3/_NCNT was compared with a Co_3_O_4_ nanoparticles-based air electrode.

Among the wire-based zinc-air batteries, Xu et al. [148] rolled zinc wire (0.25 mm Ø), around a SS rod creating this way a zinc spring. This spring was inserted in a PET tube where the hydrogel polymer electrolyte solution, 8.3 wt.% PVA, 0.83 wt.% PEO, and 8.3 wt.% KOH, was poured in. The whole assembly was kept in a refrigerator at −20 °C for 2 h to crosslink the electrolyte solution. The cathode includes aligned and cross-stacked CNT sheet for the oxygen reduction reaction (ORR) and a RuO_2_—based catalyst for the oxygen evolution reaction (OER). This fiber-shaped Zn-air battery was rechargeable, as shown by discharging/charging at 2 and 1 Ag^−1^ at discharging voltages of 0.9 and 1.0 V. Same approach was used by Meng et al. [230] but using a Zn belt (2 mm wide) instead. The zinc belt was rolled around a SS rod and covered with PVA-KOH hydrogel. The resulted assembly was carefully wrapped with Co_4_N/CNW/CC to enclose the gel electrolyte-coated zinc spring as the cathode. Finally, a punched heat-shrinkable tube was used to protect the resulting Zn-air battery. The 3D freestanding bifunctional electrode was composed of Co_4_N, carbon fibers network (CNW), carbon cloth (Co_4_N/CNW/CC), by pyrolyzation of ZIF-67 (Zeolitic Imidazolate Frameworks) on polypyrrole (PPy) nanofibres network rooted on carbon cloths. The idea behind this bifunctional air electrode catalyst is combining metallic Co_4_N with superior OER activity and Co-N-C with perfect ORR activity. The as-built flexible Zn//PVA-KOH//Co_4_N/CNW/CC battery displays excellent rechargeable performance at a high current density of 0.5 and 1 mAcm^−2^. Zhao et al. [231] also combined metallic FeNi NPs with higher OER activity and NCNTs with wonderful ORR activity. Thus, the Zn foil//PVA-KOH//FeNi@NCNTs/CC battery showed a high discharge voltage (≈1.0 V @ 2 mAcm^−2^), a charge voltage ≈1.65 V @ 2 mAcm^−2^, and a power density of 7 mWcm^−2^, along with an excellent mechanical and cycling stability. Meng and Zhao did not provide conductivity values for the gel electrolytes used in their works.

Besides, some other authors also used PVA blends to study electrochemical properties of the gel electrolytes as well as the performance of them on zinc-air batteries. Thus, Wu et al. [149] studied alkaline PVA/PAA-KOH polymer blends as membranes by ac impedance spectroscopy and the electrochemical stability window of the PVA/PAA examined by cyclic voltammetry analysis. The percent use of the cell capacity was as high as 90% when the PVA:PAA = 10:7.5 polymer electrolyte membrane was employed and the cell was discharged at C/10 rate, the power density reached 50 mWcm^−2^. Besides, this work was devoted also to study Al-air batteries with the same GPEs.

Kim et al. [150] fabricated a electrospun polyvinyl alcohol (PVA)/polyacrylic acid (PAA) nanofibre mat anion-conducting continuous phase. Into the PVA/PAA nanofibre mat, Nafion bearing pendant sulfonate groups are impregnated by immersion to form an anion-repelling continuous phase (PBE). Such bicontinuous phases enables the PBE membrane to act selectively on ion transport, suppressing Zn(OH)_4_^2−^ crossover, by Donnan exclusion effect [232], slightly impairing OH^−^ conduction. The PVA/PAA (optimized ratio, 7/3w/w) nanofibers was subjected to thermal treatment in order to allow intermolecular condensation reaction between PVA and PAA prior to being impregnated by Nafion leading to the formation of the PBE membrane. Notably, the zinc–air battery with the PBE membrane showed the electrochemical rechargeability over 2500 min (at 20 mAcm^−2^ and 10 min cycle period), compared to the cell with the Celgard3501 (900 min).

Song et al. [151] fabricated a PVA, PAA, and graphene oxide (GO) co-crosslinked alkaline GPE, named PVAA–GO. Their approach is a holistic one toward the design of a ZAB. The highly hydrophilic PVAA–GO network hydrogel exhibited essentially improved water retention capability and alkali uptake behavior compared to the conventional PVA and besides, the introduced reaction modifier KI in the GPE changes the path of the conventional oxygen evolution reaction leading to a more thermodynamically favorable path. One immediate advantage, the lower charging voltage turn into less degradation of the carbon-supported catalyst electrode during charge. The fabricated PVAA–GO was soaked in a 4 M KOH + 2 M KI solution, to obtain KI–PVAA–GO, which was used as GPEs in a cable-type and sandwich type zinc-air batteries. Thus, the Zn//KI–PVAA–GO//P/C + Co_3_O_4_/CNF/CC battery could deliver a working time of up to 200 h.

Gan et al. [233] designed a GPE based on PVA and poly(diallyldimethylammonium chloride) (PDDA) cross-linked with glutaraldehyde, to uniformly coat ZnO particles obtaining a ZnO/PVA/PDDA composite used as anode in the zinc-air battery. The continuous and flexible structure of PVA/PDDA film helps to retain zinc active material near the electrode, demonstrated by measuring zincate concentration in the electrolyte by complexometric titration with EDTA, which will lead to restricted shape change meaning a uniform distribution of current and smaller polarization of zinc electrode. The battery was, actually, a liquid battery using the gel polymer electrolyte as glue to keep ZnO particles near the anode and eventually preventing the Zn oxidized species to travel to the bulk electrolyte. Stock et al. [234] followed also the same confinement concept of oxidized zinc species in the anode, a concept that improves rechargeable batteries with zinc anodes. Anion exchange ionomers (AEI), quaternary ammonium AEI type commercials, AS-4 (5 wt.% in 1-propanol; Tokuyama) and FAA3 (10 wt.% in N-methyl- 2-pyrrolidone; FumaTech) or KOH doped meta-polybenzimidazole (PBI; 2 wt.% with 2.5 wt.% KOH in ethanol; FumaTech), 4.0 ± 0.5 µm thick were used to coat zinc electrodes and then soaked in KOH 4 M to obtain the OH^−^ form of the AEI. Galvanostatic discharge and charge profiles of all AEI-coated Zn electrodes showed longer cycling with higher total discharge capacities compared to the uncoated electrodes, but none of the electrodes were tested in a lab scale prototype battery.

In another quaternary ammonium functionalized GPE, Lin et al. [224] proposed a strategy to engineer AAEM (alkaline anion exchange membrane) for flexible ZABs by constructing ion-conducting nanochannels as well as switching conventional alkali-type into the near-neutral-type membrane. QA-functionalized PVA (QAFP) was exchanged with NH_4_Cl solution instead of KOH solution as to get a near-neutral type membrane. The exceptional water retention and high ion conductivity of QAFP resulted according to DFT calculation to strong bonding between water molecules and hydrogen’s quaternary ammonium shaping this way water nanochannel. Hydroxide transport in a water channel was studied using the CI-NEB method (Climbing Image Nudged Elastic Band Method), hydroxide transport is much easier on QAFP due to its lower energy barrier (0.18 eV) than that of PVA (0.47 eV), well-organized water channels on QAFP allows rapid hydroxide transport in a confined and directional manner. Finally, the assembled battery, Zn//GPE//Co_3_O_4_@NCNTs/CC was tested with to GPEs, 2-QAFP-6 (neutral) and 2-QAFP-14 (alkaline). The 2-QAFP-14–based battery has a higher discharge potential than the 2-QAFP-6–based battery, but fail to give longer discharge tines. Thus, the neutral 2-QAFP-6 was able to delivers energy density of 1440 WhL^−1^ (based on Zn foil volume) and specific energy density of 223 Whkg^−1^ (based on the total mass of active materials), which is better than the Li-ion battery of the Nissan Leaf (140 Whkg^−1^).

Nanoporous carbon nanofibre films (NCNFs) with large specific surface area prepared by high-temperature pyrolysis of electrospun polyimide (PI) under Ar atmosphere was used as cathode by Liu et al. [235] in a liquid Zn-air battery showing excellent performance with a high open-circuit voltage (1.48 V), maximum power density (185 mWcm^−2^), and energy density (776 WhKg^−1^). To demonstrate potential applications in a flexible/wearable device, a flexible all-solid-state Zn-air battery composed of, a freestanding NCNF-1000 air-cathode (pyrolyzed at 1000 °C), zinc foil anode, PVA gel electrolyte (KOH/ZnAc_2_) was fabricated. The calculated specific capacity and energy density of this all-solid-state battery were 378 mAhg^−1^ and ≈378 WhKg^−1^, respectively. The strength of this work, according to the authors, is the facile method for scalable synthesis of nanoporous carbon nanofibres films (i.e., NCNFs) simply by high-temperature pyrolysis of electrospun polyimide fiber films apart from the catalytic activity toward ORR, which is even better than that of Pt/C. In fact, the performance of the flexible batteries using either NCNF 1000 or Pt/C as catalysts is quite similar.

Porphyrin is a polar, conjugated, and highly delocalized organic molecule with strong ability to coordinate metal cations into complexes. These characters gift porphyrin and porphyrin derived structures as promising candidates for bifunctional oxygen electrocatalysis. Several authors have studied Porphyrin as catalyst for ORR/OER reactions, but unfortunately Porphyrin shows low affinity to conductive scaffolds and tend to aggregate itself [236]. Trying to overcome this drawbacks Li et al. [236] used porphyrin covalent organic framework (POF) in order to use it as bifunctional catalyst in zinc-air batteries. The cathode CNT/POF showed better performance than its Pt/C + IR/C counterpart did. The superb performance of the rechargeable liquid battery encouraged the authors to use the cathode in a flexible zinc-air one. Thus, the flexible Zn foil//GPE//CNT/POF battery with PVA gel (KOH + ZnCl_2_) showed a high OCV of 1.39 V, small voltage gap, and maximum peak power density of 22.3 mWcm^−2^ but lacked to demonstrate the long-term capability of the flexible battery. The authors did not provide either conductivity values for the gel electrolyte.

#### 3.2.3. Zn-Ni

The up-to-date energy storage market continues to be dominated by lithium-ion batteries despite numerous safety incidents and obstacles, including transportation restrictions, constrained resource supply of lithium and cobalt, high cost and limited or in its embryonic state recycling infrastructures [237]. Despite these disadvantages, Li-ion batteries are widely used because they provide high energy density, high specific power, and long cycle life, attributes that must also be met by any other technology to compete for market share [238]. The electrochemical performance of Ni–Zn batteries are moderately satisfactory according to Table 1, specifically for rate performance and cycling stability, only outperforming Lead-acid and comparable to Ni-Cd and Ni-MH. Gel polymer electrolyte can overcome associated problem in Zn-Ni-based secondary batteries employing liquid electrolytes, namely the change in shape of the Zn-based electrode and the dendritic growth.

PVA is, to date, the most used polymer to fabricate gel polymer electrolytes for electrochemical devices. It has been also used in other batteries technologies such Zn/Ag and Zn/Ni in either yarns other coplanar types [152,239,240,241,242,243,244,245]. Thus, Huang et al. [241] reported large-scale-yield yarns, similar to cotton yarns, wearable energy storage textile, fabricated by weaving a large conductive cloth from the conductive yarns with the use of a commercial weaving machine. They managed effectively to powers various electronic devices including a watch, a set of light-emitting diodes, and a pulse sensor. Wearable high-performance NiCo//Zn batteries are fabricated by coating both electrodes with a PVA-KOH-Zn (CH_3_COO)_2_ gel. Same line of research is followed by Zeng et al. [242] using a fiber-shaped Ni–NiO//Zn battery, based on Ni–NiO heterostructured nanosheet cathode. Compared to the NiO//Zn battery, the Ni–NiO//Zn battery delivers a much lower voltage hysteresis and longer discharge plateaus, suggesting its less polarization and higher capacity. As electrolyte, PVA-KOH gel electrolyte, saturated with ZnO, was also used. The battery was capable of running up to 10,000 cycles and achieving an impressive power density of 20.2 mWcm^−2^. Liu et al. [243] developed a prototype of a 1.77 V flexible planar quasi-solid-state Ni–Zn battery, CC-CF/ZnO//GPE//CC-CF/NiO. In the cell, ZnO nanoparticles and NiO nanoflakes are deposited on a 3D hierarchical carbon cloth-carbon nanofiber in both the anode and the cathode, respectively. The carbon cloth is evenly coated with 3D N-doped CF arrays, serving as a substrate with high surface area for loading with the active material, besides, the conductive CFs provide excellent electrical conductivity. The performance of the battery with the PVA-KOH GPE is comparable to that of the liquid electrolyte and better than other technologies. Similarly, Yan et al. [244] reported also a planar Zn-Ni battery; Graphene/ZnO//GPE//Ni-Al LDH, layered double hydroxide. In this case, ZnO nameplates were deposited on graphene and then the GPE (PVA soaked in 6 M KOH) was sandwiched between the two electrodes. According to the authors, the battery was able to exhibit 526.19 Whkg^−1^ at the current density of 1 Ag^−1^ based on the total mass of active materials and a maximum power density of 85.69 kWkg^−1^. Remarkably, when the current density increased to 50 Ag^−1^, the battery retains 74.32% of initial capacity at 1 Ag^−1^.To further assess the potential feasibility of the flexible quasi-solid-state Ni-Zn secondary battery for actual applications, two flexible quasi-solid-state Ni-Zn secondary batteries were connected in series to power a 3 W high-brightness white LED and 5 W cooling fan. Li et al. [245] also reported a fibrous shaped Ni/Zn battery in which a 3D lithium doped TiO_2_ nanotube-array fiber-shaped electrode (Li-RTiO_2_). The battery Zn@Li-RTiO2//GPE//Mn-NiOx reveals robust long-term durability in Zn containing alkaline solution and the impressive charge/discharge behavior at an ultrahigh rate. Besides the battery Li doped was compared to a non-doped one showing much better Capacity and less charge/discharge potential gap due to the poor electrical conductivity of TiO_2_.

#### 3.2.4. Zn-Ag

Li-ion batteries enabled the development of literally hundreds of innovative electronic products, and they will continue to do so in the future. However, the spur for smaller, safer, and more energy-dense power sources has increased the research on more energy-dense power sources. Silver-zinc directly addresses many of the design, safety, and power issues. The energy density of the zinc/silver oxide system (zinc/alkaline electrolyte/silver oxide) is among the highest of all battery systems making it ideal for use in small, thin ‘‘button’’. Most of these batteries are now prepared from the monovalent silver oxide (Ag_2_O) instead of divalent silver oxide (AgO), last one delivers higher capacity though, due to dual voltage discharge curves and greater instability in alkaline solutions of AgO [98].

The major advantages of the zinc/monovalent silver oxide battery are high energy density, good voltage regulation, high rate capability, flat discharge curve, comparatively good low-temperature performance, leakage risk negligible, good shelf life, and some others; however, the high cost renders this kind of batteries only suitable for specific applications.

Zamarayeva et al. [152] developed a wire flexible Zn-Ag battery dip-coating the Zn anode into the PVA-KOH polymer gel and packing the whole assembly in a chemically resistive heat shrink tube. Migration of silver ions to the zinc electrode and subsequent poisoning of the electrode was avoided in part by a PVA gelled electrolyte. The mechanical flexibility and stability of the wire battery anode depends critically on the morphology of electrodeposited Zn, which can be controlled by adjusting the plating current, on the copper wire. In this sense, a vast number of technical papers were published lately.

**Figure 6:**
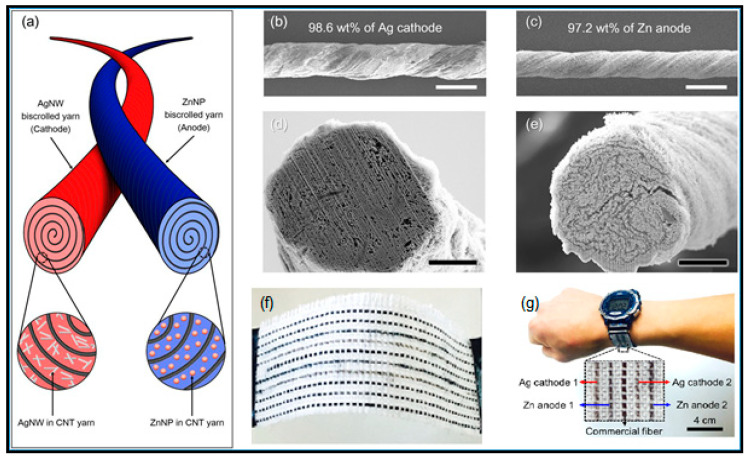
(**a**) Schematic illustration of yarn battery consists of Ag nanowire/CNT and Zn nanoparticle/CNT electrodes. SEM images showing (**b**) the Ag yarn electrode (scale bar = 300 μm), (**c**) the Zn yarn electrode (scale bar = 300 μm), (**d**) the cross-section of the Ag electrode (scale bar = 20 μm), and (**e**) the cross-section of the Zn electrode (scale bar = 20 μm). (**f**,**g**) Photographs of commercial electric watch operated by two serial connected Ag-Zn yarn battery woven in textile watchstrap. Reproduced with permission from [240]. Copyright 2018 Nature.

Li at al. [246] used another approach trying to mitigate the migration of Ag ions into the gel covering the cathode with a layer of PEDOT:PSS (poly(3,4-ethylenedioxythiophene) polystyrene sulfonate) with no influence on the rate performance of the Zn–Ag_2_O battery. This protective layer suppressed also structural pulverization of the cathode. Fiber-shaped electrodes were twisted together with PVA-KOH gel to form a battery, CNTF-NCA-Zn//GPE//PEDOT:PSS/Ag_2_O/NCA- CNTF being NCA, nitride-doped nanocarbon array, and CNTF, carbon nanotube fibers. The battery delivered 14.4 mW/cm^2^, long-term durability, sustaining >79.5% of the initial capacity and high coulombic efficiency (≈100%) after 200 cycles. Similarly Lee et al. [240] twisted the Ag electrode up to 2000 times per meter around the zinc electrode to form a fiber-shaped CNT-Zn//GPE//Ag-CNT battery (Figure 6). Ag up to 99 wt.% and Zn up to 98 wt.% could be loaded onto the CNT yarn electrodes by biscrolling method [247]. Due to high active material loading, the yarn battery showed in solid electrolyte a capacity of 0.276 mAhcm^−1^ just a bit lower than the one obtained using liquid electrolyte, which are, according to the authors, significantly higher than previously reported fiber batteries.

Chaowei et al. [239] combined a solar cell with a planar flexible quasi-solid-state Ag-Zn battery based on a novel MOF derived Ag nanowire binder-free cathode. The as-prepared quasi-solid-state CC/Zn nanoflakes//PVA-KOH//Ag-MOF/CC Zn batteries exhibit a high capacity of 1.245 mAhcm^−2^, a remarkable energy density of 1.87 mWh/cm^2^, and maximum power density of 2.8 mW/cm^2^ due to the abundant reaction sites and short electron and ion diffusion paths of the MOF derived Ag nanowires electrode. The morphology of Ag-MOF presents quasi cubic structure and the sizes are about 1~2 μm. Migration of silver ions to the zinc electrode and subsequent poisoning of the electrode was avoided in part by a PVA gelled electrolyte as stated by Zamarayeva [152], but in this case an extra separator is added. As a proof-of-concept, a commercial solar cell was attached to the battery making possible charging the battery by an integrated flexible commercial solar cell.

#### 3.2.5. Zn-Ion

The charge storage process of ZIBs relies on the migration of Zn ions between anode and cathode. ZIB uses the similar working principle of a LIB with its “rocking-chair” design, which was put forward by Kang and colleagues in 2012 [248]. Kumar et al. [201] in 2003 already reported and proposed a mechanism for the reversible intercalation/deintercalation of Zn ions in γ-MnO_2_ using a gel polymer electrolyte (GPE) with Zn(CF SO_3_)_2_ salt. Despite the already known lower conductivity, respect to liquids electrolytes, GPEs has been also used in zinc-ion systems reducing dissolution of electrode materials and avoiding the growth of dendrites. However, liquid electrolytes are the most common choice yet in Zn-ion batteries [102,249].

Huang et al. [153] at Nankai University developed a self-healing zinc-ion battery which it is of interest to avoid relative displacement or detachment of components under bending situation that is very normal when dealing with fabrics. According to the authors, the driving force producing the healing of the GPE, is the formation of hydrogen bonds between neighboring chains when the gel was cut, recovering the hydrogel, eventually, the ionic transport and mechanical strength thereafter. The layers of SWCNTs (Single-Walled Carbon Nanotubes) in the battery configuration Zn(SWCNT)//PVA//PANI(SWCNT) will also display better characteristic to regenerate the current flow path of cathode and anode after self-healing.

Cao et al. [250] in a very interesting approach, used a sponge based on PANI-SWCNT supported on Ti foil as cathode, to which a liquid solution of PVA/Zn(CF_3_SO_3_)_2_ was added. In this way, the PANI-SWCNT-based sponge together with PVA/Zn(CF_3_SO_3_)_2,_ will form the cathode-gel electrolyte assembly. To this assembly, filter paper soaked in PVA/Zn(CF_3_SO_3_)_2_ was set to the side in contact with the Zn foil anode. The most interesting feature of this battery was its compressibility. The capacity of such compressible ZIB can remain 98.4% of initial capacity after 1500 cycles even at a compressive strain of 60%.

Qiu at al. [251] used (3D) porous surface nitrogen-doped carbon cloth (N-CC) as flexible structural supports, and then evenly deposited MnO_2_ nanorod arrays and tiny Zn nanoparticles as the cathode (N-CC@MnO_2_) and anode (N-CC@Zn) respectively. N-CC hold great potential for energy storage since they not only afford higher surface areas, more active sites, and better permeability than other nanostructures, but also can dramatically improve the conductivity while maintaining electrochemical and structural stability. The highest specific capacity of 328 mAhg^−1^ is achieved for the quasi-solid-state N-CC@MnO_2_//GPE//N-CC@Zn battery at 0.5 Ag^−1^. They compared the performance of the battery in both liquid electrolyte and GPE. The flexible battery device reached a maximum of 469 WhKg^−1^ using liquid electrolyte and 440 WhKg^−1^ with PVA/lignocellulose/ZnCl_2_/MnSO_4_ gel electrolyte based on both cases on MnO_2._

In 2014, Zhang et al. [252] demonstrated a unique liquid aqueous zinc-ion battery based on the zinc anode and Zn_3_[Fe(CN)_6_] and K_2_Zn_3_[Fe(CN)_6_]_2_-based cathodes (Prussian blue analogs [253]). This cell exhibited an average operation voltage of 1.7V. In comparison with α-MnO_2_ (ca. 1.3 V), ZnHCF has a higher Zn^2+^ intercalation voltage leading to a higher operation voltage. On the other hand, Zinc hexacyanoferrate nanocubes with manganese oxide (ZnHCF@MnO_2_) prepared using an in situ co-precipitation method was used by Lu et al. [254] as cathode in a flexible GPE (ZnSO_4_/PVA-based), with neutral electrolyte, battery. The combination of capacitive properties of manganese oxide and intercalative feature of ZnHCF, in a unique structure, is able to adjust the charge storage process. In addition to that the encapsulation of ZnHCF nanocubes with manganese oxide nanosheets is able to maintain overall mechanical integrity, improving the cycling stability. The fabricated flexible device shows discharge capacities of; 89, 78, 67, 58, and 53 mAhg^−1^ at 100, 200, 400, 500 and 800 mAg^−1^, respectively, and can be cycled over 500 times with capacity retention of ~71%.

Zeng et al. [255] used a flexible 3D carbon nanotube (CNT) network as highly conductive skeleton for Zn deposition to achieve dendrite-free Zn/CNT anode. As a consequence, the Zn/CNT anode showed prolonged cycling life up to 200 h (≈28% DOD), and dendrite-free surface. Moreover, a high CE (97.9%) was also achieved thanks to the boosted reversibility of Zn plating/stripping on CNT compared with CC. Similarly, Zhang et al. [256] used also a flexible 3D carbon nanotube (CNT) conductive networks as excellent electron and charge transfer substrates. CNTs arrays anchored on the surface of carbon cloth (CC) substrate by chemical vapor deposition (denominate Cs) was coated with a layer of MnO_2_ (denominated CMO) and subsequently decorated on the surface by a second electrochemical deposition process with a fine coating of conductive PEDOT to further stabilizing the core–shell structure and mitigating the dissolution loss of MnO_2_ upon cycling (denominated CMOP). The electrochemical behavior of CMO, and CMOP electrodes together with MO, MOP (electrodes without CNT array) were evaluated by using electrodeposited Zn anode and 2 M ZnCl_2_ + 0.4 M MnSO_4_ electrolyte. The high rate performance has been considered to be a pivotal indicator, making batteries preferable for applications, such as quick charging of cellphones and regenerative braking of electric vehicles. In this regard, the CMOP electrode with 3D ion channels and short electronic transport path is much favorable for accelerating charge transfer at the electrode/electrolyte interface. In view of the superior energy storage performance of CMOP cathode, a flexible electrodeposited Zn//GPE//CMOP achieved 17.5 mWh·cm^−3^ (379.4 Wh·kg^−1^) and peak power density of 0.8 W·cm^−3^ (17.1 kW·kg^−1^).

It would be very interesting to extend the work done by Zhang et al. [257] with liquid electrolyte to GPEs. In this work a chemically self-charging aqueous ZIBs system, is integrated in a single CaV_6_O_16_·3H_2_O (CaVO) cathode. It can harvest energy from ambient environment through spontaneous redox reaction, and then convert chemical energy into electrical energy and store them in ZIBs. Therefore, the resultant ZIBs can be self-recharged by directly exposing CaVO cathodes to air without any external power supply.

#### 3.2.6. Zn-Bi_2_O_3_

Bismuth oxide (Bi_2_O_3_) is a nontoxic and low cost metal oxide that has been studied in depth and applied in different fields, such as catalysts, optical materials, or gas sensors. In energy storage systems, this material has attracted a great deal of interest due to its good reversibility properties [258]. Very recently Lorca et al. [259] studied the cyclability of a battery composed of a Zn negative electrode and a Bi_2_O_3_ positive one have been tested, using a PVA-KOH gel polymer as the electrolyte. Specific capacity efficiencies higher than 90% with respect the initial Bi_2_O_3_ content have been reached during the discharge process. Besides, a low gap between the discharge and charge voltages was observed, indicating a high energy efficiency in a cycle, which is essential for its commercial application.

The most frequently used synthetic homopolymer for hydrogels fabrication is the PVA, which contains abundant hydroxyl hydrophilic functional groups. The ubiquitous alkaline PVA-KOH gel is the easiest way to have and hydrogel but it suffers from excessive water loss, low elasticity, and moderate mechanical strength, what makes it not the best option for GPEs in batteries, especially zinc-air batteries due to their intrinsic semi-open architecture. This excessive water loss is regarded as PVA-KOH gel’s Achilles heel. However, along this section, it has been seen PVA hydrogel; cross-linked, physically and chemically, easily processed by biscrolling or electrospun, quaternized with ammonium salts, knitted in textiles, blended with others polymers, showing overall its versatility. PVA-based hydrogels can accept different type of salts, namely: KOH, ZnAc_2_, ZnTf_2_, ZnCl_2_, ZnSO_4_, etc., and also being saturated with ZnO showing chemical compatibility. The crosslinking methods for PVA hydrogels can be by either physical methods including H-bonding or using chemical crosslinking agents, endowing them with a certain degree of stretchability and elasticity due to the reversible interactions and much better water retention capacities and even self-healing properties. These properties make them suitable to fabricate, as seen along this section, many types of geometries flexible batteries encompassing several zinc chemistries. With these arguments, we strongly believe PVA has its own niche within the big GPE family.

### 3.3. Polyacrylamide (PAM) and Its Derivatives

Polyacrylamides are high molecular weight water swell-able polymers formed from acrylamide or its derivatives. PAM is a non-ionic, water-soluble, and biocompatible polymer that can be tailored to meet a broad range of applications such water thickening, food packaging, soil conditioning, protein separation, etc. but also as gel polymer electrolytes for energy storage [260]. Adding cross-links between polymer chains affect the physical properties of the polymer depending upon the degree of cross-linking rendering them insoluble in water. Thus, the uncrosslinked polyacrylamides are soluble in water, and crosslinked polyacrylamides can absorb water but are insoluble [261]. PAM is a charge–balanced polyampholyte random copolymer with high water retention ability, due to the electrostatic interactions between the electrostatic groups and water molecules, and possess an antipolyelectrolytic effect, where inclusion of salt ions in the solution causes swelling of the hydrogel instead of the collapse that occurs in ordinary polyelectrolytes. The polyampholyte and anti-polyelectrolyte effect favors their use in high electrolyte solutions due to their ability to bind to low molecular weight substances such metal ions [262].

**Figure 7:**
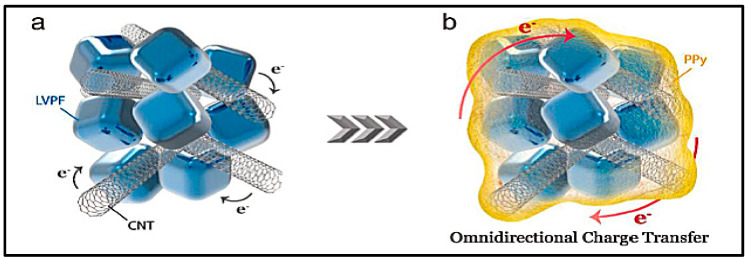
Schematic illustrations of (**a**) the CNTs networking for LVPF and (**b**) the CNTs networking + PPy coating for LVPF. Arrows indicate the efficient omnidirectional charge transfer. Reproduced with permission from [263]. Copyright 2019 Wiley.

The all-purpose material such PAM have been used as GPE in dual-ion batteries. Liu et al. [263] reported a flexible solid-state aqueous zinc hybrid battery with flat and high-voltage discharge plateau with hierarchically structured LiVPO_4_F (LVPF) cathode and a highly concentrated dual-ion hydrogel electrolyte. The poor Electronic conductivity of LiVPO_4_F was boosted by (CNTs) networking as “scarf” and polypyrrole (PPy) wrapping as “blanket” (Figure 7).

To address the concern of the narrow voltage window of aqueous solutions, a highly concentrated dual-ion electrolyte (HCE) based on the concept of “water-in-salt” electrolyte was used to allow the high-potential redox reactions of LiVPO_4_F. An HCE-based gel polymer electrolyte (HCGPE) was developed by using crosslinked polyacrylamide (PAAm) as polymer matrix with a breaking stress of 28.53 kPa and tensile strength of (1.93 ± 0.33) kPa. The highly concentrated 21 M LiTFSI + 2 M Zn(Tf)_2_ (HCGPE) not just retained water but also absorbed moisture from the ambient, showing high hygroscopicity. Such a feature addresses the concern of battery encapsulation and electrolyte replenishment and endows the flexible battery with high durability. The liquid battery exhibited a flat high-voltage discharge plateau of nearly 1.9 V, a capacity of 146.9 mAhg^−1^ at 0.2 Ag^−1^ and an energy density of 235.6 Whkg^−1^. The solid stated Zn//GPE//LVPF-CNTs@PPy battery exhibited a rate performance with specific capacities of 141.2 mAhg^−1^ at 0.2 Ag^−1^ and 58.3 mAhg^−1^ at 2.0 Ag^−1^, which are 96.5% and 85.2% of the capacities of its liquid electrolyte-based counterpart, respectively. In the battery, Zn is stripped and deposited in the anode and Li^+^ is intercalated and de-intercalated in the LiVPO_4_F.

#### 3.3.1. Zn-MnO_2_

**Figure 8:**
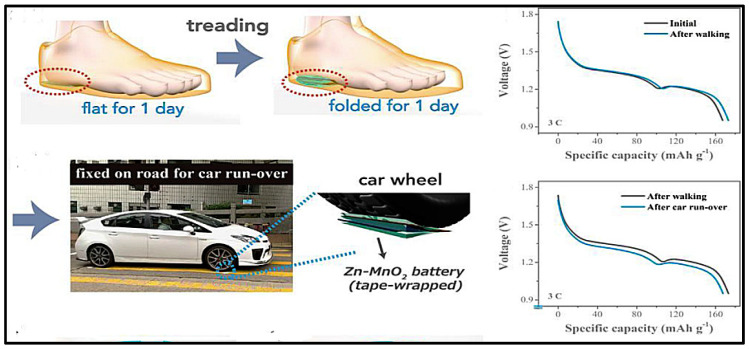
Illustrations of the Zn-MnO_2_ battery being placed under foot and going through car run-over and discharge curves of the battery after 2 days’ everyday treading and after 20 times of random run-over by cars on road. All the discharge curves were recorded at 0.924 Ag^−1^ (3C rate). Reproduced with permission from [154]. Copyright 2018 Elsevier.

As stated in the PVA section, alkaline zinc-manganese dioxide (Zn-MnO_2_) batteries have long dominated the battery market. Zn-MnO_2_ batteries have also been envisaged using PAM or PAM blends. Thus, Liu et al. [154] reported a highly soft yet super-tough Zn-MnO_2_ battery by employing a dual-crosslinked energy-dissipative hydrogel electrolyte. The hydrogel electrolyte is based on the framework of covalently crosslinked polyacrylamide (PAAm) together to a second ionic network into the PAAm network by crosslinking alginate chains with Zn^2+^. The battery was able to delivers 300.4 mAhg^−1^ at 0.11 Ag^−1^ and 82% capacity retention after 500 charge-discharge cycles at 0.88 Ag^−1^. The flexible battery hold excellent mechanical durability through a series of mechanical tests, such folding, squeezing, twisting, hammering, etc. The battery was even attached to a car tire (Figure 8) and was run over for 20 times. The performance recorded after test completion showed not apparent noticeable change on it.

#### 3.3.2. Zinc-Air

The different electrochemical storage systems have different performance in terms of specific energy and power; rechargeable batteries are typically characterized by low specific power and high specific energy, while the opposite applies for the electrochemical capacitors. However, highenergy–highpower devices are sought being able to fulfill the high output requirements of today’s demanding applications. One way to accomplish such task is the hybridization of electrochemical capacitors with rechargeable batteries, combining the high specific power of the electrochemical capacitor with the high specific energy of the battery [264].

Other way to accomplish the formerly is with batteries that have dual-functional cathodes. Thus, Longtao Ma and his group at the Department of Materials Science and Engineering City University of Hong Kong have been very active developing hybrid zinc batteries. However, previously, and base of the subsequent work, they developed a “firstly reported” high reversible battery Zn/Co(III) rich-Co_3_O_4_ working well in a liquid mild aqueous electrolyte solution (2 M ZnSO_4_ + 0.2 M CoSO_4_) [155]. Co(III) rich-Co_3_O_4_ nanorods by introducing excess cobalt salt in the synthesizing process confirmed by the increase of relative intensity of Co(III)-O/Co(II)-O in Raman spectra. Based upon the good results obtained for the liquid battery, they built a freestanding flexible battery Zn/CC//GPE//Co_3_O_4_/CC, where the GPE (PAM/ZnSO_4_/CoSO_4_) showed a ionic conductivity up to maximum value of 0.12 Scm^−1^ at 300% swelling ratio. The electrochemical reaction of the Zn/Co(III) rich-Co_3_O_4_ battery with ZnSO_4_-CoSO_4_ mild electrolyte can be summarized:

Co_3_O_4_ + Zn + (x/3)H_2_O + ⅓ZnSO_4_ ↔ 3CoO + ⅓ZnSO_4_[Zn(OH)_2_ ]_3_∙(x − 1)H_2_O

**Figure 9:**
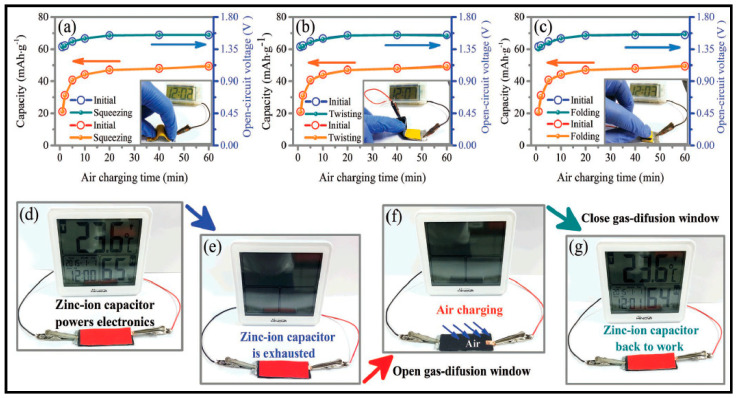
Demonstration of the “air chargeable” zinc-ion capacitor and its operation under various deformations: Capacity versus “air charging” time and corresponding open-circuit voltage of the capacitor under (**a**) squeezing, (**b**) twisting, and (**c**) folding and compressing deformations. (**d**–**g**) Demonstration of the process of “air chargeable” zinc-ion capacitor. (**d**) A zinc-ion capacitor is used to power a digital hygrometer, and (**e**) the zinc-ion capacitor is exhausted after working for several hours. (**f**) Then, the sealing tape is removed and air diffuses in and “air charging” is triggered. (**g**) The zinc-ion capacitor is successfully charged and starts to power the hygrometer again. Reproduced with permission from [156]. Copyright 2019 Wiley.

The reaction in mild electrolyte is significantly different from that in alkaline one, with lower voltage 1.9 V and poor cycling stability due to the irreversible reaction of Zn + 2OH^−^ → Zn(OH)_2_ +2e^−^ → ZnO + H_2_O. In contrast, in mild 2 M ZnSO_4_ + 0.2 M CoSO_4_ aqueous electrolyte, exhibited a high voltage of 2.2 V, and high cycling stability of 5000 cycles with 92% capacity retention. In a second step, they created a solid-state rechargeable and washable Zn-Co_3_O_4-x_/air hybrid battery with high water-retaining polyacrylamide (PAM) hydrogel electrolyte and high dual-functional cathode of oxygen vacancies rich-cobalt oxide of Co_3_O_4_-x [265]. As a hybrid battery, the battery comprises two electrochemical processes: process I, first plateau at 1.92 V from Co-O-H → Co-O Faradaic redox reaction, and process II, second plateau at 1.25 V resulting from ORR reaction. The hybrid battery hold high power density of 3200 W·kg^−1^ and energy density of 1060 Wh·kg^−1^ using liquid electrolyte and subsequently, a flexible hybrid battery based on solid-state hydrogel electrolyte was fabricated, being capable of working both under water and/or in air. The same group in another approach [156] proposed a highly integrated system of “air charging” zinc-ion capacitor/battery with a “U” shaped air electrode with dual functions (energy conversion and storage). The “U” shaped battery/capacitor contained a common zinc foil in the middle with different GPEs at both sides, PAM soaked in 2 M ZnSO_4_ for the capacitor side and sodium polyacrylate (PANa) soaked in Zn(Ac)_2_ 0.2 M and 6 M KOH for the air charging side. The system was able to work in three modes: at discharging mode, the system will work as an ordinary zinc-ion capacitor. The self-charging mode (the second mode) is triggered by unsealing the air electrode: the zinc-air components will spontaneously start to work and charge the zinc-ion capacitor. At total charging mode, both the zinc-ion capacitor and zinc-air component will be fully charged by external power supply (Figure 9). They used a MOF (Metal Organic Framework) specifically a Zeolitic Imidazolate Frameworks (ZIF) to develop a porous carbon fibers Co-N-C ZIF-67-based cathode, used in both zinc-ion capacitor for charge storage, and air electrode for energy conversion. However, the outlook of batteries using cobalt could become overcast due to all issues related to cobalt [266] despite the interesting results obtained. In addition, cobalt cannot be considered to be a benign and friendly material as the Ma group stated. This hybrid battery/capacitator system is also evaluated by Sun et al. very recently [267]. In this case, no cobalt related material is used in the cathode, but a defective reduced graphene-based cathode (defective RG cathode), which is considered ideal since the RG surface oxygen-containing functional groups can chemical interact with the divalent metal ions (such as zinc ions) in electrolyte, boosting its capacitive storage ability and would provide rich active sites for ORR.

Tan et al. [157] synthetized a freestanding GPE planar sheet, derived from a highly crosslinked homo-polymer, which enabled bendable metal-air batteries with high performance in the absence of any kind of mechanical supports. The crosslinked polymer, polyacrylamide (PAM), was synthesized via an ultraviolet (UV) light-initiated radical polymerization of acrylamide monomers using *N*,*N*′-methylenebis-(acrylamide) (MBA) as the crosslinkers. Soaking the PAM films in KOH aqueous solution led to the formation of freestanding alkaline GPEs which were used in Zn-air batteries with a power density of 39 mWcm^−2^ and steady cycling over 50 h at a stable discharge and charge potential of 1.2 and 2.0 V, respectively.

**Figure 10:**
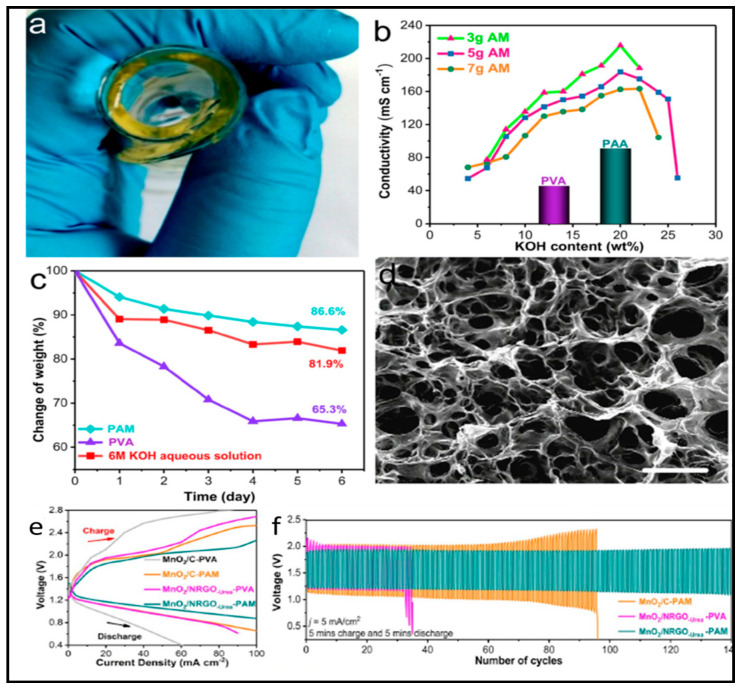
(**a**) A photograph of PAM-based AGE with excellent flexibility. (**b**) Ion conductivities of PAM-based AGEs versus the KOH mass fractions and AM contents. (**c**) Weight changes of PAM, PVA-based AGEs and KOH solution exposed in the ambient air environment. (**d**) SEM image showing the microstructures of PAM-based AGE. Scale bar: 100 mm. (**e**) Charge-discharge polarization curves of flexible ZABs. (**f**) Charge-discharge cycling curves of flexible ZABs. Reproduced with permission from [158]. Copyright 2020 Elsevier.

Very recently Miao et al. [158] developed oxygen catalyst of MnO_2_ nanowires supported on nitrogen-doped reduced graphene oxide (MnO_2_/NRGO-Urea). They integrated this cathode in a flexible ZAB PAM-based, obtaining a maximum power density of 105.0 mWcm^−2^ and great flexibility and robustness. The flexible ZAB with MnO_2_/NRGO-Urea and PAM-based GPE, delivered a stable discharge voltage around 1.2 V with a slight degradation. The calculated specific capacity at 10.0 mAcm^−2^ is as high as 720 mAhg^−1^ normalized to the mass of Zn consumption (theoretical capacity of 820 mAhg^−1^). They established a comparative study of the ionic conductivity of three different alkaline gel electrolytes, namely PVA, PAM, and PAA and cycling stability comparison between PVA and PAM-based flexible batteries (Figure 10).

#### 3.3.3. Zinc-Ion

Li et al. [12] used a hierarchical gelatine and PAM-based electrolyte and α-MnO_2_ nanorod/CNT composite cathode to explore the use of this flexible batteries to power sensor for collecting movement. The hierarchical polymer electrolyte is synthesized by grafting polyacrylamide (PAM) onto gelatine chains that are filled in a network of a polyacrylonitrile (PAN) electrospun fiber membrane. The grafting of PAM onto a gelatine hydrogel (gelatine-g-PAM) significantly enhanced the mechanical strength and ionic conductivity (1.76 × 10^−2^ Scm^−1^) of the HPE film. The battery was submitted to a comprehensive series of destructive tests, such soaking, heating, punching, bending, etc. demonstrating its safety as wearable flexible battery. The flexible battery showed an energy density of 6.18 mWhcm^−2^, power density of 148.2 mWcm^−2^, a specific capacity of 306 mAhg^−1^, and a capacity retention as high as 97% after 1000 cycles. Proper proof of the reliability of the battery was done powering a smart watch, a pulse sensor and more surprisingly, a smart insole was powered with two batteries. These smarts insoles usually consist of various sensors, which can collect movement data, it was even possible to run outside for over 2.1 km or walk for 80,000 steps with the ZIB-powered smart insole.

In another step forward in order to integrate this types of batteries in textiles, Wang et al. [159], from the active Department of Materials Science and Engineering, City University of Hong Kong, in the research field of flexible batteries, developed a sewable Zn–MnO_2_ based on a nanofibrillated cellulose (NFC)/polyacrylamide (PAM) hydrogel, electrodeposited Zn nanoplates as anode, and carbon nanotube (CNT)/α-MnO_2_ as cathode. This battery, unlike the yarn type seen so far, it is considered finished fabric to be sewed, being the sewed battery able to withstand a high shear force more than 43 N. The NFC/PAM hydrogel can be easily stretched to 1100% strain, with no visible crack or breakage an its ionic conductivity increased up to 22.8 mScm^−1^ respect to the PAM hydrogel without nanofibrillated cellulose (165.9 mScm^−1^).

**Figure 11:**
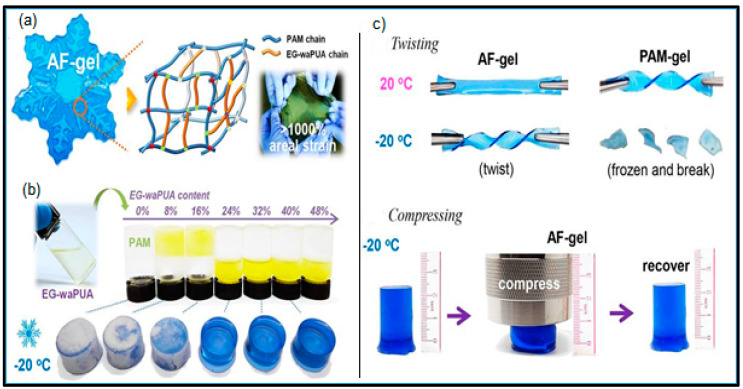
Characteristics of the AF gel electrolyte. (**a**) Optical images of the AF gel (colored blue for visibility) and a hypothetical molecular model. The soft hydrogel can be stretched more than 1000% in area without fracture. The dots with different colors indicate hydrogen bonds among different species (**b**) The effect of EG-waPUA weight percentage (Gw%) on the freeze-resistant performance of the hydrogel electrolyte after one day of cooling at −20 °C. The EG-waPUA/water solutions were stained by yellow ink (0.1 wt.%). (**c**) Elastic stability of AF gel at −20 °C. Reproduced with permission from [160]. Copyright 2019 Royal Society of Chemistry.

**Figure 12:**
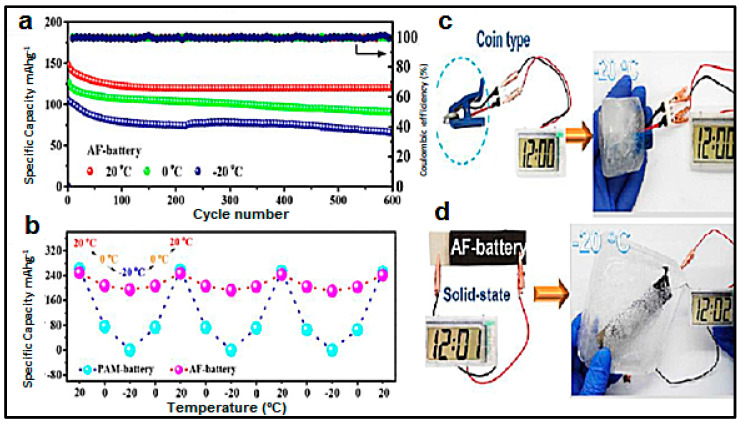
(**a**) Extended cycling performance at 2.4 Ag^−1^ of the AF-battery at different temperatures. (**b**) Cyclic testing of PAM-battery and AF-battery under 20, 0, −20 °C at 0.3 Ag^−1^. Battery in ice package. Freeze-resistant performance of the (**c**) coin-type and (**d**) solid-state flexible AF-batteries. Reproduced with permission from [160]. Copyright 2019 Royal Society of Chemistry.

It is a concern for aqueous zinc-ion batteries to work at temperatures below 0 °C degrees, since the water in aqueous electrolytes would freeze and inhibit the transportation of ions with this, detrimental effects on the battery performance. Quan et al. [268] proposed an anti-freezing gel electrolyte that contains polyacrylamide, graphene oxide, and ethylene glycol. According to the authors GO provided help to construct a three-dimensional macroporous network that facilitates ionic transport, whereas EG improved freezing resistance. δ-MnO_2_ was used as cathode material for its large interlayer distance, which can easily accommodate foreign cations such as Zn^2+^ ions. The Zn/GPE/δ-MnO_2_ battery delivered a discharge capacity of 284.8 mAhg^−1^ at 0.2 Ag^−1^ at 20 °C and 183.2 mAhg^−1^ at −20°C. When the same battery was tested with neat PAM the obtained results for discharge capacity were lowers. Similarly, Mo et al. [160] also used PAM hydrogel crosslinked covalently with EG-waPUA (ethylenglycol waterborne polyurethane acrylates, end capped) in order to synthetized an hydrogel to be used in wearable devices with antifreeze properties. The idea behind using such complex hydrogel is to avoid that the alcohol molecules belonging to the EG or other anti-freezing additives damage the intrinsically weak mechanical properties of physically cross-linked hydrogels. Hence, the alcohols molecules must be anchored onto the polymer chains through covalent bonds to form a stable unified matrix. The anti-freezing mechanism of the AF gel was also studied by density functional theory (DFT) calculations. Thus, in the polymer networks the multiple interactions can firmly lock water molecules and disrupt the formation of crystal lattices, endowing the AF gel with excellent anti-freezing property (Figure 11 and Figure 12).

To demonstrate the usefulness of the AF gel, a piece of the as-synthesized AF gel electrolyte, serving also as the separator, was sandwiched between a flexible zinc anode and α-MnO_2_/CNT cathode to assemble a full cell. The ionic conductivity of the AF gel electrolyte (2 mol L^−1^ ZnSO_4_ and 0.1 mol L^−1^ MnSO_4_) was 16.8 mScm^−1^ at RT, at −20 °C the ionic conductivity was14.6 mScm^−1^. The AF-battery exhibited a volumetric energy density of 32.68 mWh·cm^−3^, as well as a specific capacity of 275 mAhg^−1^ at 20 °C. Significantly, the discharge capacity retentions of the AF-battery at 0 and −20 °C compared with that at 20 °C are 88.36% (243 mAhg^−1^) and 82.18% (226 mAhg^−1^) at 0.2 Ag^−1^ with high cycling stability of 81.67% (0 °C) and 74.54% (−20 °C) capacity retentions over 600 cycles at 2.4 Ag^−1^. The same group reported a reversible sol-gel transition electrolyte with PNIPA-co-PAA, poly(N-isopropylacrylamide-co-Acrylic acid) dissolved in ZnSO_4_ and MnSO_4_, for thermal self-protection of batteries. The denominated PNA sol-gel electrolyte, allows the zinc ions freely migrate between electrodes in the sol state at low temperature. Once heated to a certain temperature, the sol-gel electrolyte forms stationary hydrogel that inhibits the diffusion of zinc ions, thus shutting down the batteries. The process can be reverted backing the battery on service again. The concept about active self-protection of thermoresponsive batteries, battery was tested on a rechargeable Zn//GPE//α-MnO_2_ sandwich type battery, where GPE was a PAN membrane soaked in the PNA sol-gel electrolyte [269].

As a typical conducting polymer, polyaniline (PANI) shows high conductivity, multiple reversible redox/doping states, and good stability in water and air rendering it a good candidate to construct flexible batteries. Thus, Xiao et al. [161] built a flexible solid-state zinc battery with a sandwich structure by using the PANI/CNT as the cathode, Zn/CC as the anode and the PAAM-ZnSO_4_ gel cross-linked with *N*,*N*′-methylenebis-(acrylamide) (MBA). During the discharge process, Zn/CC is oxidized and PANI is reduced with the de-doping of SO_4_^2−^, while during the charge process, Zn^2+^ deposits on the anode and PANI is oxidized with the entrance of SO_4_^2−^. Hence, the overall reaction can be described as:

nZn + PANI (ES) (SO_4_^2−^)_n_ ↔ nZn^2+^ + PANI (LE) + nSO_4_^2−^

Being ES, and LE leucoemeraldine (PANI oxidized form) and emeraldine (PANI reduced form) respectively. The flexible battery shows a specific capacity of 144 mAhg^−1^, and excellent cycling stability with a capacitance retention of 91.1% after 150 cycles. In addition, the as-prepared Zn showed no noticeable change in its electrochemical performance under mechanical bending tests.

Zhao et al. [270] developed an Zn/GPE/[EMIM]PF_6_-PEDOT:PSS/Bi_2_S_3_ with a PAM-based GPE and 1 M Zn (TFSI)_2_ and 2 M LiTFSI as salts. Bi_2_S_3_ has never been used in aqueous ZIBs due to the structure degradation. Thus, ultra-high-conductivity IL-enhanced PEDOT:PSS network successfully stabilize the B_i2_S_3_ cathode for aqueous ZIBs by inhibiting grain pulverization and sulfur dissolution indicating that the existing irreversible reactions are reduced at higher charge/discharge rates. The as-fabricate flexible quasi-solid battery has an energy density of 315 Whkg^−1^ and long-term lifespan over 5300 cycles. As a practical demo, a single battery powered a digital hygrometer thermometer for more than 14 h.

**Figure 13:**
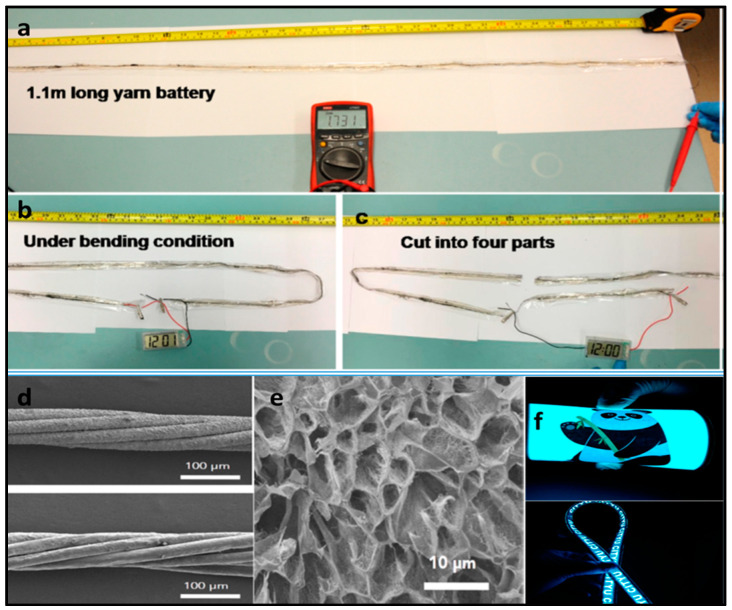
Tailoring test and demonstration of solid-state yarn ZIBs. (**a**) Optical image of the as-prepared 1.1 m long yarn ZIB. (**b**) Long yarn ZIB under bending condition. (**c**) Long yarn battery was cut into four parts, and each part can power the digital watch. (**d**) SEM images of the CNT@Zn yarn (**top**) and SEM images of the CNT@ MnO_2_ yarn (**bottom**). (**e**) SEM image of the freestanding PAM. (**f**) Eight segmented yarn batteries were connected in series to power a 100 cm^2^ electroluminescent panel (size 10.0 × 10.0 cm) under different bending conditions and a 1 m long electroluminescent panel (size 1.0 × 100 cm). Reproduced with permission from [162]. Copyright 2018 the American Chemical Society.

Yarn type electrodes (Figure 13) for “smart textiles or E-Textiles” have been also used with PAM GPEs. Thus, Li [162] from City University of Hong Kong designed a quasi-solid-state washable and tailorable elastic yarn ZIB constructed by double-helix yarn electrodes and a polyacrylamide (PAM)-based polymer electrolyte. They synthesized the yarn electrodes from pristine double-helix carbon nanotube CNT yarns. By roll-dip-coating and roll-electro-deposition processes, they manufactured the MnO_2_ yarn cathode and zinc yarn anode continuously in a very facile way based on the CNT fiber as shown in the article Supplementary Information. The prepared PAM electrolyte containing 2 mol L^−1^ ZnSO_4_ and 0.1 mol L^−1^ MnSO_4_ showed a conductivity of e 17.3∙10^−3^ Scm^−1^. When subjected to a strain of 300%, still showed a high ionic conductivity of 16.5∙10^−3^ Scm^−1^. The battery delivered a specific capacity and volumetric energy density (302.1 mAhg^−1^ and 53.8 mWhcm^−3^, respectively) as well as excellent cycling stability (98.5% capacity retention after 500 cycles).

To evaluate the durability and stability as well as the waterproof capability, the battery underwent tests to assure its reliability under strain, bending, cutting, knotted and immersion conditions. The waterproof capability of the battery was achieved thank to an Eco-flex silicone coating. Finally, eight segmented yarn batteries were connected in series to power a 100 cm^2^ electroluminescent panel, Figure 13f.

PAM can also be considered to be one of the most important material for hydrogels production. It is hard to say, in this quick evolving research field of the GPE, which of the material base to produce hydrogel is taking the lead. According to the research showed in this review, it seems PAM is prone to be used as energy-dissipative dual-crosslinked hydrogel, by the rupture of sacrificial covalent bonds of the brittle network, or antifreezing hydrogel. Its compatibility with more complex hybrid systems is also demonstrated. Furthermore, its thermo-responsive behavior through a reversible sol-gel transition in PNIPA-co-PAA copolymer and the processing into a variety of battery shapes due to its tunable viscoelastic behavior make this material a serious candidate to be used for the massively deployment of power sources in wearable electronics.

### 3.4. Poly-Acrylic Acid (PAA) and Sodium Polyacrylate (PANa)

Hydrogel networks formed from poly-(acrylic acid) (PAA) have the ability to absorb many times their weight in water and are the basis of a class of materials called super absorbent [271] (PAM is also considered a superabsorbent). The degree of crosslinking in the polymer network structure is critical, as it will endows the hydrogel with specific mechanical strength, swelling ratio, and many other properties [163]. As polymeric host material, PAA is chemically and electrochemically stable under highly alkaline environments. The conductivity of PAA–KOH remains stable at high temperatures (360 mScm^–1^ at 65 °C) because of its high water retention. In battery testing, ZABs with PAA–KOH electrolytes have the potential to outperform batteries with conventional aqueous electrolytes (e.g., 6 M KOH) by reducing interfacial and charge transfer resistance [163]. PAA undergoes a reversible conformational transition (coil-to-globule transition) around pH 5 that is driven by the state of ionization of the carboxylic group. At low pH, PAA adopts a compact (but not fully collapsed) globular conformation. However, as the pH is increased, ionization occurs and the polymer expands into a fully solvated open coil conformation [272]. Usually, ionic conductivity measured to PAA-KOH has been frequently reported higher than the PVA–KOH one. These properties make PAA as a good candidate to replace PVA as the most polymer host used in GPEs for alkaline batteries.

Gaikwad et al. [164] reported a new way of fabricating Zn and MnO_2_ for flexible battery purposes addressing the thickness and capacity limitations of thin-film flexible batteries without compromising the power performance, simply embedding active material on meshes. To do so, 50-mesh size nylon meshes were used as support for both electrodes with silver ink stencil printed onto the underside of the mesh to form the current collector. 200-mesh size nylon mesh was used as separator and drop-cast with PAA-KOH-ZnO-based GPE. They studied PAA-based GPE with different weight ratios. Contrary to expectation, the conductivity of the PAA-based GPE increased with increasing PAA concentration, leading to a bigger quantity of aqueous electrolyte absorbed.

**Figure 14:**
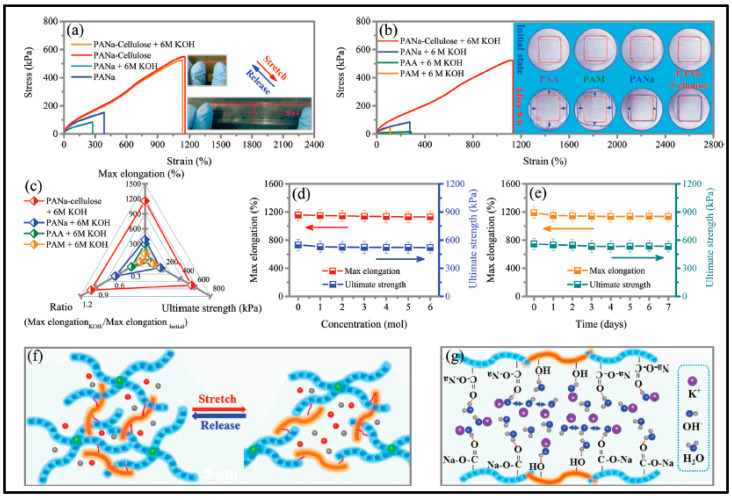
(**a**) Tensile stress versus strain curves of the as-synthesized PANa and PANa-cellulose hydrogel electrolyte with and without 300% 6 M KOH + 0.2 M Zn(CH_3_COO)_2_ intake. The insets are optical photos of the relaxed and elongated states of the 300% 6 M KOH + 0.2 M Zn(CH_3_COO)_2_ solution incorporated PANa-cellulose hydrogel electrolyte showing excellent stretchability. (**b**) Comparison of tensile properties of PAA, PAM, PANa and PANa-cellulose hydrogel under alkaline condition. The inset is the photos of PAA, PAM, PANa and PANa-cellulose hydrogel at initial state and containing 300% 6 M KOH solution for 8 h. The red and blue rectangle represent the shape of hydrogel before and after infiltrating alkaline solution. (**c**) Comparison of alkaline-tolerant capability of different hydrogel electrolyte. (**d**) Ultimate strength and maximum elongation of PANa-cellulose hydrogel electrolyte containing KOH solution with different concentrations. (**e**) Ultimate strength and maximum elongation of PANa-cellulose hydrogel electrolyte containing 300% 6 M KOH solution after different alkaline corrosion times. (**f**) Schematic illustration for origin of ultra-stretchability. (**g**) Schematic diagram reflecting structure of PANa-cellulose hydrogel electrolyte entrapped KOH and water via the interactions of hydrogen bonds.Reproduced with permission from [273]. Copyright 2019 Wiley.

The ever-present group of Longtao Ma created an alkaline-tolerant dual-network hydrogel, Figure 14, electrolyte-based sodium polyacrylate (PANa) and cellulose [273]. The cross-linking among PANa, *N*,*N*’-methylenebisacrylamide (MBA), and cellulose is responsible for mechanically strengthening and keeping high stretching ability even after in strong alkaline, 6 M KOH, soaking. Thus, the hydrogel showed, after soaking, over 1000% stretchability, without any breakage or even visible cracking, together with a 0.28 Scm^−1^. At this point, the last work moves us to summarize and clarify the relationship between PVA, PAM and PAA/PANa and their healing and energy-dissipative properties in dual network hydrogel already, or to be, mentioned in this review in several references [153,154,168,273,274]. To this aim, the work done by Gong et al. [275] is taken as reference. These networks with differentiate structures are separately cross-linked by covalent or noncovalent bonds, and the interpenetration of the two network makes the DN gels simultaneously both tough and soft. Upon deformation, the first network broke and dissipated the energy, while the second network is able to retain the whole integrity of the gel. Gong and co-workers studied the effect of different ratio of comonomer on the self-healing efficiency of a PVA/(PAM-co-PAA) dual network gel. The observed gel healing efficiency is higher when the number of PAA segments exceed that of PAM segments, presuming that there are more H-bonds formed between; in a fresh cut interface, hydroxyl and carboxylic group provided by PVA and PAA respectively. Another factor affecting the strength of the dual network are the PVA crystallites formed which act as physical cross-links to hold the network structure in the hydrogel. However, PVA crystallites in the gel scatter light and affect transparency, so that measuring transparency, the strength of the dual network can be followed. Finally, the free hydroxyl and carboxyl groups are responsible for the H-bonds that can be formed across the interface when the two surfaces are brought together. As mentioned, Double network (DN) gels show higher toughness by sacrificial fracture of the brittle network and weak physical bonds, dissipating energy, whereas the covalent network holds the whole structure. This concept can be broadened, changing the weaker and brittle polymeric network by ceramics and minerals such phosphate salt or hydroxyapatite [276].

In addition, PAA has been used to strengthen PVA by simply soaking PVA hydrogels obtained by the typical freezing-thawing method in PAA solutions. Thus, super-strong and tough PVA/PAA hydrogels reinforced by hydrogen-bonding were obtained. Up to 140 MPa of tensile strength were obtained which can be considered a tough material. The soaking or immersing strategy is more effective for reinforcing the PVA hydrogel than simply casting a mixture of both components [277]. Usually PAA is cross-linked using MBA, (*N*,*N*’-methylenebisacrylamide) but it can also be done with 2D graphene oxide as to get mechanically tough and highly stretchable hydrogels [278].

Zhu et al. [279] synthetized a PAA-KOH-H_2_O gel by polymerization of the Acrylic acid monomer with *N*,*N*’-methylene-bisacrylamide (MBA), KOH, and K_2_S_2_O_8_ as initiator. The polymer electrolyte exhibited a maximum ionic conductivity of 0.288 Scm^−1^, about 60% of the maximum value of KOH solution. It has to be noted that PAA synthesized at low KOH concentrations, the GPE conductivity was higher than the liquid KOH solutions with the same concentration, which can be explained because a larger number of ions could be obtained at low KOH concentration due to the dissociation of the coordinated K^+^ cations on the polyacrylate chains. PAA-KOH-H_2_O membranes were also successfully applied as electrolyte in Zn/Air, Zn/MnO_2_ and Ni/Cd batteries. The result obtained demonstrated similar performance characteristics for the three alkaline batteries, as well as the same chemical and electrochemical stability as in aqueous alkaline solution.

#### 3.4.1. Zn-MnO_2_

The engineering possibilities and availability of specific materials to make ideas coming to life have enabled research such the one done by Lao-Atiman et al. [280] engineering transparent Zn/MnO_2_ battery. Transparent batteries are very unusual in comparison to electrochromic devices. The battery could have had an extra feature, such flexibility, should the authors had used flexible conducting substrate instead of brittle ITO. The cathode was prepared by screen-printing the whole cathode area (40 mm × 40 mm) with the MnO_2_ functional ink onto the cathode current collector and the Zn ink was then deposited onto the anode current collector as micro-electrode array (75%, 80% and 85% opening area). The alkaline GPE film was prepared by polymerization of the solution of acrylic acid and KOH mixed solution with addition of MBA as a crosslinker and K_2_S_2_O_8_ as a polymerization initiator. The energy density of this battery was about 3.16 mWhcm^−2^ at 1 mAcm^−2^ and about 2.95 mWhcm^−2^ at 2 mAcm^−2^.

#### 3.4.2. Zinc-Air

Kwon et al. [281] developed a transparent bendable secondary zinc-air battery using PAA 10 wt.% 6 M KOH poly-acrylic acid (PAA) gel polymer electrolyte. The anode was fabricated electrodepositing zinc on SS mesh. This allows packing densely the active material in a confined space closer to the current collector gaining higher energy density without significant volume growth. An anionic exchange polymer (AEP) resin layer, FAA-3-SOLUT-10 in NMP, FUMATECH BWTGmbH, incorporated on to the cathode mesh, as separator, became the key component in the fabrication process. The AEP resin was prone to adhere to the cathode wires, as the cathode mesh settles on top of the AEP resin, the resin gradually adheres to the wires leaving void spaces behind in the open areas of the mesh, which facilitates the light way through. The cathode was obtained spraying a slurry of Pt/C and Ir/C on the same SS mesh. The performance of the secondary zinc-air battery was not an outstanding one in term of current density, ~701 mAhgZn^−1^ at 1 mAcm^−2^. However, as the authors themselves remarks, the aim of the work was not to develop a battery with exceptional power density, nor to develop new materials that perform outstandingly, but to develop a straightforward method to create batteries with newer physical properties.

Chaduang et al. [282] used commercial Carbopol 940 (crosslinked PAA) to highlight the improvement of discharge/recharge performance on a flexible Zinc-air battery. Likewise, Wang et al. [283] develop a separator based on a paper soaked in PAA/PVA solution to enhance the battery performance. The main special feature of the battery is the addition of MWCNTs to the anode, which was effective keeping zinc particles together. Not only MWCNT was added to the anode, PEDOT:PSS was also added as co-binder. Mohammad [284] used commercial hydroponic gel, gelled with KOH 6M to develop a zinc-air battery which showed no sign of degradation neither in the cathode nor in the GPE.

Huang et al. [165] used a PAA-based GPE, specifically PANa (sodium polyacrylate), also considered to be superabsorbent, trying to improve the low water take-up and retention, weak interaction with electrodes, and structural instability intrinsically associated with common polymer electrolytes. PANa hydrogel can absorb more than 52 times its own weight of the concentrated solution (0.2 M Zn (CH_3_COO)_2_ + 6 M KOH used in this work. Thus, Zn–air rechargeable batteries based on the PANa electrolyte, exhibited high capacities (≈800 mAhgZn^−1^, comparable to their liquid-state counterparts) and good cycling stability. Besides, the electrostatic interactions between the acrylate ions along the PANa backbone and Zn ions facilitated the formation of quasi-SEI, effectively eliminating zinc dendrites that were prevalent in aqueous and PVA-based electrolytes. PANa network greatly facilitated the ionic transport with a drastically increased ion conductivity with increasing water content to reach a remarkably high ionic conductivity of 0.17 Scm^−1^. Huang et al. [170] also tested the same hydrogel in ZnCo/Ni batteries. This in situ building of a protective solid electrolyte interface (SEI) coated Zn anode exhibits highly reversible (100% CE) efficiency.

Tran et al. [163,167] prepared PAA-KOH mixing AA (acrylic acic) monomer, *N*,*N*′-methylene-bis-(acrylamide) as crosslinker agent, potassium persulphate as thermoinitator in a KOH and ZnO, or Zn(Ac)_2_ solution and studied varying the concentration of each components. They concluded that ZnO is a more effective additive than Zn(Ac)_2_. Besides, the lowest concentration of AA tested (0.5 M) and the highest one of crosslinker (30 mM) provided the best results. The ZAB using PAA-KOH-ZnO GPE showed a specific energy of 913 WhkgZn^−1^ and more than 160 cycles were performed.

Guan et al. [227] applied a similar synthesized PAA-KOH-ZnO GPE for preparing flexible sandwich-like layered and coaxial fiber-shaped solid-state ZABs, using Zn negative (a foil or a microwire) and NC-Co/CoNx (hybrid cobalt/cobalt nitride nanoparticles with nitrogen-doped carbon) positive electrodes. Compared to sandwich-like ZABs, the fibre-shaped battery provided similar open-circuit potential, but narrower voltage gap between charge and discharge processes. Besides, density discharge capacity of 344.7 mAcm^−1^ and power density of 104.0 mWcm^−1^ were obtained for the fiber-type battery, whereas lower values of 96.6 mAcm^−1^ and 41.5 mWcm^−1^ resulted for the layered battery, demonstrating the much better properties for the fiber battery.

There are multiple combinations of polymers often used as host in the GPEs. A representative example of copolymerization was reported by Z. Cao et al. [168], who designed and synthesized a poly(acrylamide-co-acrylic acid) alkaline GPE, named as P-(AM-co-AA), to be applied in a planar zinc-air battery. The aim of the authors was to improve the mechanical properties of the PAA-KOH polymer, which can retain a high amount of water but, great retention, makes a pure PAA gel weak and brittle because the formed hydrogen bonds are constantly challenged by surrounding water molecules. The synthesis was carried out by a free radical copolymerization of acrylamide and acrylic acid, using the *N*,*N*’-methylenebisacrylamide (MBA) as the cross-linker. The polymer obtained was soaked in a 6 M KOH and 0.2 M Zn(Ac)_2_ solution before the battery assembly. The as-prepared P-(AM-co-AA), before soaking in KOH solution, contains a high number of acylamino and carboxylic groups, which forms multiple H-bonds between them and with water. These interactions generate a crosslinked network with a great water retention capability, conferring to this copolymer excellent mechanical property. However, when the polymer is immersed in the 6 M KOH solution, a degradation of the mechanical properties was observed, probably due to the deprotonation of carboxyl groups in high pH solutions, provoking dissociation of H-bonds between some acylamino and carboxyl groups. In any case, the mechanical properties are still superior to those of PAA or PAM alkaline gels, confirming the effectiveness of the copolymerization process. Besides, the authors claim a better conductivity values for this copolymer, 0.148 S cm^−1^, with respect to the PVA, 0.077 Scm^−1^. This behavior agrees with the SEM image obtained after freeze-dry procedure, a network of channels was observed for P-(AM-co-AA) whereas a relatively smooth profile with no obvious channels was observed for PVA. This result indicates that the channels structure found for P-(AM-co-AA) facilities the filling of the gel with plenty KOH solution, increasing the ionic transport. However, here it has to be noted that PVA used in this work was prepared with KOH 1 M solution, but higher conductivities have been reached using higher concentrations of KOH solutions [51].

#### 3.4.3. Zn-Ni

The cost, safety, and performance advantages of NiZn batteries make them yet well suited for a variety of applications. NiZn batteries are non-flammable, are recommended in applications where physical safety is essential and perform well at high discharge rates.

Iwakura et al. [169] tested a PAA-KOH GPE, fabricated directly from PAAK (potassium polyacrylate) and KOH with no previous polymerization step, in a Ni/Zn battery and it was compared with a similar cell but using 7.3 M KOH liquid solution. The last showed unstable and poor charge/discharge performance after a low number of cycles, which was ascribed to the dendritic formation in de Zn electrode. However, use of a PAA-KOH membrane improved the charge/discharge behavior, and no dendritic growth was observed. The PAA-KOH GPE reached a staggering conductivity value of 0.6 Scm^−1^.

Lie et al. [166] took advantage of the fact that PANa (sodium polyacrylate) hydrogel can maintain good performance under alkaline conditions and it can keep its stretchability and compressibility when it absorbs many ions to develop a flexible rechargeable NiCo//Zn alkaline. PANa hydrogel was obtained from AA monomers and then soaked in a concentrated solution of KOH and Zn (CH_3_COO)_2_. Anode and cathode share an Au sheet to further improve the conductivity of CNT papers. For the fabrication of the stretchable battery, the PANa polyelectrolyte was first pre-stretched over 400% strain. The flexible and stable battery Zn/Au/CNT//GPE//NiCo/Au/CNT cycles and compressed for 1500 cycles, was able to delivers 87% and 97% of its initial capacity and up to a discharge rate of 23 Ag^−1^ after being stretched for 500.

Another work recalling the self-healing properties of the GPEs was carried out by Huang et al. [274] using FeCl_3_ as crosslinker in PANa hydrogel which was soaked in 0.2 M Zn(CH_3_COO)_2_ and 6 M KOH to provide the charge carriers. The ferric ion-mediate self-healing mechanism was demonstrated when results were compared to those obtained with a PANa hydrogel with no FeCl_3._ The healing was confirmed visually upon two broken parts set aside of a Zn//GPE//NiCo battery powering a watch. After they were brought again together, the watch turned itself back on with little delay and with no obvious changes in the brightness.

**Figure 15:**
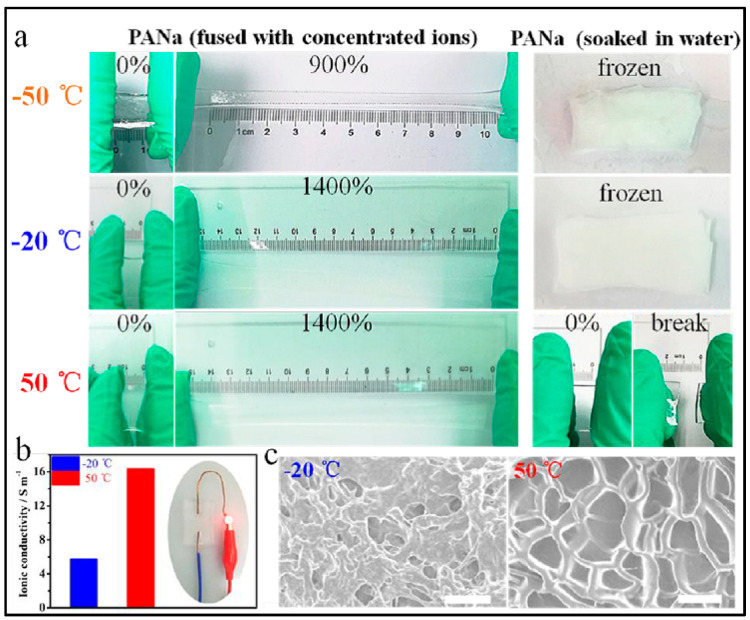
Physicochemical characterization of the electrolyte and the electrode. (**a**) The stretchability of the PANa hydrogel at different temperatures infused with concentrated ions and deionized water, respectively. (**b**) Ionic conductivities of the highly concentrated PANa hydrogel at different temperatures. The inset image shows the PANa hydrogel electrolyte used as an ionic conductor to successfully connect the LED circuit. (**c**) SEM images of the freeze-dried PANa hydrogel at different temperatures (**left**: −20 °C, **right**: 50 °C). Reproduced with permission from [170]. Copyright 2019 The American Chemical Society.

To enable practical application, portable and wearable electronics must operate in conditions such as freezing winter and scorching summer, in other words, these devices must withstand a wide operating temperature range (−20–50 °C). Most reported batteries with wide operating temperature ranges use organic electrolytes and ionic liquids. However, for aqueous batteries, freezing will results in low ionic conductivity what strongly hinders practical applications as it has already commented for PAM-based GPEs. A highly concentrated electrolyte reduces the proportion of water and therefore prevent the formation of hydrogen bonds. However, gel polymer electrolytes, generally are incompatible with high concentrations of ions such as OH^−^ and Zn^2+^, making them brittle. The former stands as starting point for Wang et al. [170] to get a highly salt concentrated hydrogel after soaking a sodium polyacrylate hydrogel (PANa), synthesized from acrylic acid monomers and ammonium persulphate as initiator, in a 6 M concentrated solution of KOH and 0.2 M Zn (CH_3_COO)_2_. As shown in Figure 15, the PANa hydrogel soaked in concentrated ions can be easily stretched to 1400% in both cold (−20 °C) and hot (50 °C) environments The hydrogel showed conductivities of 0.057 Scm^−1^ at −20 °C, 0.12 Scm^−1^ at 24 °C and 0.16 Scm^−1^ at 50 °C. The flexible CC/Zn//GPE//NiCo hydroxide/CC battery was able to deliver high energy density and power density of 172 Whkg^−1^ and 6.8 kWkg^−1^ (all based on the mass of NiCo hydroxide), respectively, at −20 °C, and of 210 Whkg^−1^ and 11.6 kWkg^−1^, respectively, at 50 °C. Additionally, the battery demonstrates the outstanding cycle stability at −20 °C, it retains 87% capacity after 10,000 cycles along with a high Coulombic efficiency close to 100%.

#### 3.4.4. Zn-Ag

The need for high single use energy density batteries and its implementation in a manufacturing process flow made Braam and Subramanian [285] to follow the typical organic solar cell configuration to create a silver-zinc battery, monovalent silver oxide, for simplification purposes, using PAA as GPE-separator containing (PAA, PEO as filler, PEGDE (poly(ethylene glycol) diglycidyl ether) as crosslinker and KOH). Firstly, after stencil printing both zinc electrode and silver electrode, the acrylic solution was let to photopolymerize on top of zinc electrode. According to the authors, unlike other alkaline separators based on PVA, separators based on PAA are not susceptible to oxidation by silver oxide. The swelling capacity of the PAA was avoided by strong crosslinking with PEGDE (1 wt.%) in order to get robust gel network. PEO was also added as filler to enhance mechanical stability. The fabricated batteries, upon GPE saturation with 8.4 M KOH, demonstrated an open-circuit potential of ≈1.54–1.55 V. The capacity of the printed silver oxide battery is ≈5.4 mAhcm^−2^ at a C/2 discharge rate of 2.75 mAcm^−2^.

Tattoo-like Zn-Ag-based was the approach followed by Berchmans et al. [286] to develop an epidermal alkaline rechargeable Ag-Zn printable tattoo battery for wearable electronics. Printed Ag and Zn electrodes were electrodeposited on the active carbon. The Ag electrode was covered with gel containing 3% poly-acrylic acid (PAA) containing 6 M KOH + 1 M LiOH. The Zn electrode was covered with a gel containing 10% poly-acrylic acid partial potassium salt (PAA-K) containing 6 M KOH + 1 M LiOH saturated with ZnO. The gap between the electrodes was filled with the gel containing 3% poly-acrylic acid (PAA) and 6 M KOH + 1 M LiOH. The battery was stuck to a human’s deltoid to demonstrate the epidermal application of the new tattoo battery in real-life scenarios. In this study, two tattoo cells were connected in series to power a LED.

PAA as pH sensitive has also showed itself as a useful polymer for the preparation of GPE. It was copolymerized with PAM and cellulose to form dual network hydrogels with energy-dissipative and self-healing properties. Due to its carboxylic groups it can also be used as reinforce PVA hydrogel trough H-bonding creating tough hydrogels or being itself cross-linked with 2D graphen oxide or more typically with MBA. PANa hydrogels were obtained through chemical synthesis of PANa or directly from it or PAAK. Both PAA and PANa or PAAK have been used indistinctly in different batteries with different chemistries, even an epidermal alkaline rechargeable Ag–Zn printable tattoo battery was developed using PAA/PAAK.

### 3.5. Polyethylene Oxide (PEO)

Among polymer used as polymer electrolytes, PEO is one of the most used, and the one used in its pioneering article by Fenton and Wright [35], to break through, within the polymer chemistry field, the use of polymer as gel polymer electrolyte in electrochemical storage devices. Hitherto, many articles has been devoted to investigate the use of PEO as gel polymer electrolyte in electrochemical devices, mainly for Li-ion batteries [45,287,288,289], but also for other battery technologies, supercapacitators [31] or electrochromic devices [290]. Poly(ethylene oxide) (PEO) is a polyether compound also known as polyethylene glycol (PEG). Depending on the molecular weight, PEO is the polymer with a molecular weight above 20,000 gmol^−1^, and PEG refers to the polymer with a molecular weight below 20,000 gmol^−1^.

PEG (polyethylene glycol), have been used as gelling agent in zinc batteries as dendritic suppressor, cycling stability enhancer and corrosion inhibiter [291,292,293]. However it has been also used as main component in gel polymer electrolytes [291]. Thus, Mitha et al. proposed a gel electrolyte consisting of an aqueous electrolyte containing 2 M ZnSO_4_ and 1 M Li_2_SO_4_, a fumed silica gelling agent, and short-chain poly(ethyleneglycol) with MW of 300 g·mol^−1^ (PEG300) as the non-thixotropic gelling agent, for secondary Zn//GPE//LiMn_2_O_4_ batteries. The addition of PEG300 to the gelled battery increases reliability, exhibiting 27–40% lower corrosion current density on the Zn anode. In comparison to the reference battery, the gelled battery exhibits significantly higher cycling performance after 300 cycles at 1C rate (12% higher capacity retention).

PEO-KOH polymer has been applied mainly to Li-based batteries. However, the number of articles reported about application of PEO-KOH membrane to Zn-based batteries is very low. There are some works published in the 80th and 90th decades, but the low output demonstrated by this type of GPE, compared with those observed for the previously commented GPEs has led to give up the use of PEO as host polymer in GPEs for zinc-based batteries.

Generally, PEO-based electrolytes are considered SPE, which are out of the scope of this review, but residual solvent content, due to the fabrication method, could make them to be considered to be GPE. It is well known residual solvents can play an important role in conductivity values in SPE [113,294]. Taking this into account, some work done so far could be considered to be GPEs. Thus, Fauvarque et al. [295] in an early work, compared a PEO-KOH-based anhydrous SPE (50/50%) with a water containing one (60–30−10%) being the water containing one, the one used in Nickel/Zinc and Nickel/Cadmium cells. Surprisingly the water free SPE showed unusual high conductivity. In the aftermath, they demonstrated in another study [171] the existence of a crystalline phase or complex formed by PEO-KOH and water, depending on O/K molar ratio, in the water containing SPE, which could explain the unusual high conductivity of the water free SPE found in the first study.

In an early work, Yang et al. [172] reported the preparation and properties of an alkaline composite polymer electrolyte based on a PEO–PVA–glass-fiber-mat system. It is known from literature that polymer electrolytes blend exhibit generally better properties compared to the electrolytes using a single polymer host. Thus, combining the good salt solvating ability of PEO and the good film-forming properties of PVA better properties are expected. In general, this composite electrolyte can be applied in all alkaline batteries systems. The conductivity of the electrolyte film was around 0.01 Scm^−1^ at RT, and was composition dependent [172].

Rathika and Suthanthiraraj [173] studied the influence of 1-ethyl-3-methylimidazolium bis(trifluoromethylsulfonyl)imide (EMIMTFSI) as plasticizers on GPEs composed of PEO (90 wt.%)/PVdF (10 wt.%)]—15 wt.% ZnTf_2._ The spherulites domains seen in SEM images correspond to the crystalline phase of the PEO. These domains reach a minimum when 7 wt.% of EMIMTFSI is added to the blend and the conductivity value reached a maximum value of 1.63∙10^−4^ Scm^−1^.

Blends of PEO and PVDF-HFP have also been explored. Ye and Xu [175] studied polyethers/PVdF-HFP-based GPE with and without the addition of organic carbonates. As polyether, PEGDME possess unusually strong capability for dissolving large amounts of zinc salts. PEGDME (poly(ethylene glycol) dimethyl ether) with small quantities of ethylene carbonate has large beneficial effects on the ionic conductivity; however GPEs with EC/PC are considered not suitable for open system such zinc-air batteries due to large weight loss in TGA analysis. When a small amount of EC is added to the blend, 0.1 EC + 0.9 PEGDME, based on PEGDME membranes exhibited negligible weight loss occurs.

Zhao et al. [176] used a Pluronic copolymer, PPO-PEO, which combines the more hydrophobic behavior of the PPO with the more hydrophilic one due to the PEO, arranged in a three-block structure. The intimate contact between PHE (Pluronic Hydrogel Electrolyte) and electrode also guarantees an even and stable ion transport. Besides the wide electrochemical window of PHE with suppressed O_2_ evolution can potentially enable a range of cathode materials, such as LiFePO_4_ (LFP) and LMP (LiMnPO_4_). The most astonishing result obtained by the authors is the ability of the battery to recover its normal performance after extreme deformations. A simple cooling process can repair the fractured electrode-electrolyte interface based on the thermoreversible gelation behavior of the Pluronic hydrogel electrolyte, hence enabling in situ restoration of the electrochemical performance. Perfect wetting of the electrodes, especially in the low temperature range, is required for realizing the cooling-recovery what is provided by the amphiphilic nature of the Pluronics. Dropping a droplet of PHE on the Zn electrode spreads and penetrates into the electrode automatically within 2 min at −5 °C.

**Figure 16:**
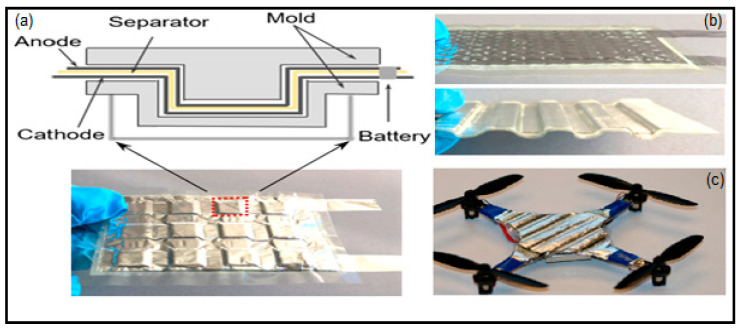
(**a**) Schematic of the mold used for plastic deformation studies. (**b**) Different plastically deformed shapes of Zn battery with solid-state biomimetic electrolyte PZB-931. (**c**) Design of corrugated Zn/γ-MnO_2_ battery pack as a replacement for the original device cover to supplement main power source of UAVs Reproduced with permission from [85]. Copyright 2019 The American Chemical Society.

Another illustrative example of PEO blends was shown by Wang and coworkers [85] at the University of Michigan together with the Harbin Institute of Technology. They have developed a branched aramid nanofibres (BANFs) and poly(ethylene oxide)-based battery. The so-called PZB-931 composite was able to adopt the round and wavy form typical in corrugated steel. Therefore, corrugated structural batteries made from stacked Zn/PZB- 931/γ-MnO_2_ cells with a size of 5 cm × 7 cm were fitted, as extra or secondary power source, to an UAV (Unmanned Aerial Vehicle) vehicle, replacing the initial factory installed structural cover of the UAV by the structural corrugated batteries, Figure 16. The full battery cell Zn//PZB-931//γ-MnO_2_ revealed discharge capacities of 146.2 mAhg^−1^, at 0.1 C and the PZB-931 showed effective dendrite suppression in a symmetrical Zn/PZB-931/Zn cell.

#### 3.5.1. Zn-Air

Similarly, Vassal et al. [296] synthetized a GPE of epichlorohydrin with ethylene oxide P(ECH-co-EO), which combines the amorphous properties of the poly(epichlorohydrin) with the solvating behavior of the poly(ethylene oxide), obtaining comparable conductivity values for the PEO/KOH/H_2_O system. The GPE presented an ionic conductivity of 10^−3^ Scm^−1^ at room temperature, for [O]/[K] = 0.32 The GPE was probed on a zinc-air battery.

#### 3.5.2. Zn-Ag

Braam and co-workers [297] following their research on Zn-Ag batteries used also PEO and methylcellulose as separator to which a certain amount of KOH was micropipetted on top becoming then a GPE. Two type of PEO with different molecular weights, *M*_W_ = 600,000 and *M*_W_ = 12.000 were used, exhibiting the first one lower internal resistance value compared to the last one The fully printed battery (Zn/Ag_2_O) with 57:29:14 H_2_O:KOH:PEO (*M*_W_ = 600,000) hat the best performance after optimization of anode and cathode formulations.

### 3.6. Biobased GPEs

GPEs made out of biopolymers has become a new prospect in the electrochemical energy storage research field [298]. Since the 1990s, the polymer industry has faced serious problems. The two most important ones being global warming and depletion of fossil resources. The use of renewable resources has become a paramount in the overall process of invention, innovation, and development in any technological change, although a biobased polymer cannot be considered per se an ecological polymer; as it depends on a plethora of factor including the material origin and the production method as the major ones. Even so, the use of biopolymer has accelerated lately due to the improvement in the chemical and biological processes involved in their production [299]. Proof of that is the massive ongoing research for the use of these polymers in the biomedical field where the number of targets application is endless [300]. The same goes for synthetic biodegradable polymers [301]. In fact, the state-of-the-art fluoropolymers, e.g., poly(vinylidene difluoride) (PVdF), are not only expensive, but require a toxic solvent (i.e., N-methyl-2-pyrrolidone, NMP) for electrode manufacturing. Greener alternatives are represented by water-processable Fluor free (F-Free) polymers, which additionally will allow a cost reduction by a factor of 2–3 for the polymer and by a factor of about 100 for the processing solvent (NMP vs. water) [302].

Bio-based polymers can be classified into three main categories based on their synthesis and origin of source. Polymers directly extracted from biomass, such as starch, cellulose, chitosan, and alginates, the second category are polymers synthesized from bio-derived monomers, and the third includes polymers synthesized by microorganisms/bacteria [55] such xanthan and gellan gums. Rayung et al. has recently written a very comprehensive review of biobased polymer electrolytes used in electrochemical storage devices [55], but there is lack of electrochemical devices based on Zinc chemistries. The excellent work done by Rayung et al. gathers in an extensive table a vast number of electrolyte systems classified by the biopolymer which form part of. The modern application of biobased GPES includes not only batteries, but also aqueous electric double-layer capacitors, dye-sensitized solar cells, and materials for proton-exchange membranes (PEMs) for fuel cells.

Here, only the most used biobased polymers in zinc-based batteries will be reviewed (Table 4). There are some others biobased polymers that have not been explored so deep to get gel polymer electrolytes for zinc-based batteries. Locust bean gum, tamarind gum, tara gum, gellan gum, dextran, Arabic gum, etc., therefore, can be also the base polymer to create GPEs for these batteries in the short term.

#### 3.6.1. Starch

Starch is mostly composed of a mixture of amylose and amylopectin; amylose is a linear polymer, whereas amylopectin has moderately branched chains instead. The ratio between the two components can differ depending on the origin, being amylopectin often the main component accounting for more of 80%.

To our knowledge, there are not many zinc-based batteries using starch as GPE however it has been more extensively used as binder [303]. Masri et al. [304] used sago powder (starch from various tropical palms) to fabricate a sago-KOH gel electrolyte. The optimum ratio of sago to water is 1:20, as to get the highest conductivity at 6M KOH. Similarly, Zahid et al. [177] obtained similar results using, cassava (Manihot esculenta) one of the most important sources of starch in tropical and subtropical areas, the optimum ratio cassava to water is 1:20 to get the highest conductivity.

#### 3.6.2. Cellulose and Its Derivatives

Cellulose is the most abundant polymer available worldwide. Cellulose has a high molecular weight and contains a linear homopolysaccharide polymer that consists of β-d-glucopyranose units joined by (1→4) glycosidic linkage, see Table 4. Recently, cellulose and its derivatives have been successfully applied in rechargeable batteries for the production of electrodes or separators or as gel polymer electrolyte [305]. Pure cellulose is not soluble in water, due to intramolecular hydrogen bonds imposing serious limitations toward its use and processability for practical applications; however, the replacement of the reactive –OH groups renders cellulose water-soluble. These reactive –OH groups present in the anhydroglucose unit, give rise, after replacement, to cellulose derivatives which are classified into cellulose ester and cellulose ether [306]. In fact, many types of cellulose derivatives have been studied, such as methyl cellulose, ethyl cellulose, hydroxyethyl cellulose, hydroxypropyl cellulose, cellulose acetate, carboxymethyl cellulose, etc. [55].

Recently, Poosapati et al. [178] synthetized NFC (nano-fibers cellulose) hydrogel which could be used as GPE in flexible batteries. The hydrogel with the appropriate amounts of gelatine, PAA, and KOH recorded an average ionic conductivity value of 0.1 Scm^−1^, but they did not subject the obtained GPE to further electrochemical test in a battery prototype.

However cellulose appear to be used extensively as separator or separator electrolyte soaked in several technologies [307,308]. Some researcher has used cellulose soaked in KOH, which can be considered to be GPE. Thus, Fu et al. from the Waterloo Institute for Nanotechnology [309] introduced a 3D nanoarchitectured rechargeable air electrode through morphological emulation of human hair array. Each individual hair-like catalyst of the array contains a nanoassembly of 2D mesoporous Co_3_O_4_ nanopetals in a 1D nitrogen-doped multiwalled carbon nanotube (NCNT). Thus, the battery Zn//Cellulose(6 M KOH)//Co_3_O_4_-NCNT/SS mesh was able to delivers, normalized to the mass of the zinc electrode, ≈652.6 and ≈632.3 mAhg^−1^ at a current density of 5 and 50 mAcm^−2^ with large gravimetric energy densities of ≈847.6 and ≈802.6 WhKg^−1^. The outstanding electrochemical performance of the Co_3_O_4_-NCNT air electrode is due to the large amount of accessible active Co_3_O_4_-NCNT nanoassemblies, which endows the battery a higher power generation per unit area of the cathode. Zhang et al. [179] also from Waterloo Institute for Nanotechnology reported a laminate-structured nanocellulose/GO membrane functionalized with highly hydroxide-conductive quaternary ammonium (QA) groups.

**Figure 17:**
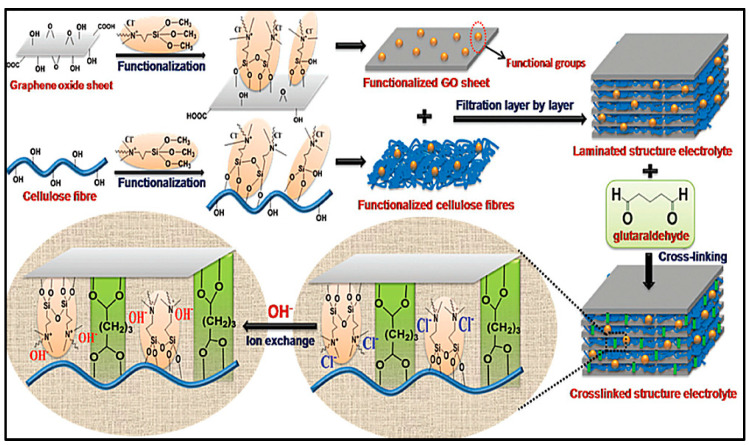
Schematic diagram of the overall preparation procedure (functionalization, filtration, cross-linking, and hydroxide-exchange) for the QAFCGO membrane. Reproduced with permission from [179]. Copyright 2016 Wiley.

The QA-functionalized nanocellulose/GO (QAFCGO) membrane is fabricated through several steps: chemical functionalization, layer-by-layer filtration, cross-linking, and ion-exchange processes. The two outer layers of GO (graphene oxide) were crosslinked with the cellulose fibers using glutaraldehyde, to guarantee its structural stability as well as low anisotropic swelling degree. In doing so, makes the whole structure self-contained, which eliminate the possibility of pushing the water out of the membrane during handling or bending, Figure 17. The good hydroxide conductivity of 58.8 mScm^−1^ at 70°C was achieved by functionalization; the enlarged d-spacing of GO nanosheets gives more spaces for the hydrated hydroxide ions to migrate via vehicle mechanism, due to its lower activation energy, without disregarding the Grotthus mechanism due to the abundance of water molecules. The QAFCGO-based zinc-air battery Zn//QAFCGO//Co_x_O_y_/CC exhibits a high open-circuit voltage of ≈1.4 V, similar to that of the A201 (commercial anion exchange membrane)-based battery. At high current densities beyond, 20 mAcm^−2^, the QAFCGO-based battery showed a remarkable advantage over the A201 battery.

In another approach Ma et al. [180] explored nanostructured PANI-cellulose papers and Zn-grown graphite papers as the flexible cathode and anode, respectively, with a cellulose nanofibres-based GPE assembled in sandwiched configuration. Flexible cathodes were fabricated by growth of PANI (polyaniline) on lens papers via in situ polymerization whereas flexible anodes were fabricated by means of electrochemical deposition of thin layers of metal Zn on the graphite paper and the gel film was prepared by swelling dry membranes of cellulose nanofibres in a liquid electrolyte, 2 M ZnCl_2_ and 3 M NH_4_Cl. The nanostructured PANI facilitates electron and ion transport as well as its use during electrochemical process and the zinc-ion battery exhibited a specific capacity of 142.3 mAhg^−1^ at a current density of 0.2 Ag^−1^ and a capacity retention of 84.7% after 1000 charge/discharge cycles.

An important issue associated with gel electrolyte membranes is their progressive release of the doped alkali because of water loss. This will cause a large ohmic polarization, which will damage the batteries’ power and energy performance. To address this issue Fu et al. [181] fabricated a thin, ultra flexible, and hydroxide-conductive quaternary ammonium functionalized cellulose membrane (2-QAFC) with high water retention, which were soaked in 1 M KOH at a later stage. The functional groups attached were further alkaline and thus the membrane became OH^−^ conductive. The water can be held strongly by hydrogen bonds and capillary forces in hydrophilic nanopores of the 2-QAFC membrane, which does not restrict the hydroxide-ion conduction. The superior hydroxide-ion conductive property and water retention boosted the specific capacity and improved the cycling stability of the zinc-air battery, compared to the commercial alkaline anion exchange membrane (A201) which was also used in the paper to compare water uptake and swelling behaviors. 2-QAFC membrane exhibited a similar positive temperature-conductivity linear relationship to the A201 membrane, indicating that the transport of hydroxide charge (OH^−^) in the 2-QAFC membrane results from the Grotthuss mechanism.

Bacterial cellulose (BC) is a naturally occurring nanomaterial produced as an exopolysaccharide by some bacteria, such as those from the family Clostridiaceae or Acetobacteraceae among others cultivated in a medium with carbon and nitrogen sources. BC has been used in the food and cosmetic industries but mainly in the biomedical area. However, the high cost of BC production is usually considered to be a limiting factor for its widespread deployment. BC has been explored also as GPE mainly, as expected, for lithium-based batteries. Although it shares the same chemical formula with plant celluloses, BC is free of lignin, hemicelluloses, and pectin, which are normally present in plant-derived celluloses, thus BC purification is easier and harmless, as the purification of plant celluloses requires harsh chemicals. In addition, BC have higher specific area higher water holding capacity respect to the plant cellulose and remarkable tensile properties due to its web-like network structure [310]. Zhao et al. [182] synthetized flexible BC/PVA composite hydrogel electrolytes (BPCE) with superior mechanical strength with microporous dual-network structure an ionic conductivity of 80.8 mScm^−1^ at RT. By adding 6 wt.% BC, the tensile strength of the entire BC/PVA membrane is increased by 9 times and the elongation at break is doubled. BPCE 6 wt.% BC was wetted in 6.0 M KOH and 0.2 M Zn(CH_3_COO)_2_ mixed solutions prior assembling the battery. Thus, the flexible Zn/BPCE wetted 6 wt.%/Co_3_O_4_@Ni ZAB battery achieved 650 cycles, over 440 h, but at a low discharging intensity of 0.5 mAcm^−2^.

Microcrystalline Cellulose (MCC) is a pure partially depolymerized cellulose synthesized from α-cellulose precursor removing amorphous regions by hydrolysis with mineral acids. In the presence of water and acid, hydrolysis process breaks cellulose polymers into smaller chain polymers or microcrystals. It is widely used in pharmaceutical industry as filler, binder, gelling agent, etc. but also in electrochemical systems.

Thus, in order to achieve a good balance between enhanced ionic conductivity and desired mechanical strength, cross-linked structures are the preferred option which are immersed in the desired solution in the aftermath, what weakens the mechanical properties. Mechanical properties of these weakened gels structures can be enhanced using MCC as filler. Thus, Zhang et al. [311] dissolved MCC in *N*,*N*-Dimethylacetamide/Lithium Chloride (DMAc/LiCl) to destruct amorphous MCC phases and to achieve the homogeneous dispersion of crystal phases. This LD-MCC solution was added to a PVA/PEO one and the resulting one mixed with the appropriate KOH solution as to get the desired gel after casting. 5 wt.% LD-MCC loading produced the highest value (0.153 Scm^−1^.) of ionic conductivity at RT. However, the electrochemical tests carried out on a ZAB prototype did not contemplate rechargeability.

A hydrogel is a three-dimensional (3D) hydrophilic cross-linked polymeric network resulting from a physical or chemical process, whereas aerogels are 3D porous network replacing liquid hydrogel by air without structural collapse. Shinde et al. [312] synthetized hierarchical sulfur-modulated 3D holey C_2_N aerogels as bifunctional oxygen catalysts and functionalized cellulose nanofibres using dodecyl-dimethylammonium chloride (DDAC) which was then immersed in 6 M KOH solution. The Zn//GPE//3D-holey-C_2_N-aerogels battery using this functionalized cellulose-KOH GPE exhibited a OCV of 1.47 V, high power density of 187 mWcm^−2^ and energy densities of 862 and 805 Whkg^−1^, when normalized to the mass of zinc, at current densities of 5 and 50 mAcm^−2^, respectively. Recently, the same research group has synthesized a choline/chitosan functionalized bio-cellulose nanofibrous membrane and was applied as GPE to a flexible rechargeable ZAB. This membrane exhibited an ionic conductivity of 64 mScm^−1^ higher than the commercial A201, a OH^−^ ion transport number of 0.734 and large water uptake (97%) [313].

Carboxymethyl cellulose can be obtained from cellulose, which is insoluble in water, by derivatization. Thus, CMC is a similar water-soluble structure derived from cellulose that consists of β-linked glucopyranose residues with partial hydroxyl groups substituted with carboxymethyl (–CH_2_COO–) groups. A biodegradable, cheap, low environmental toxicity and semi-crystalline material that exhibit an excellent film-forming ability. Among the cellulose derivatives, only NaCMC is negatively charged that can establish a strong linkage with oppositely charged materials, forming polyelectrolyte complexes. CMC is used in the form of salts, namely calcium CMC (CaCMC) and sodium CMC (NaCMC). Cross-linked CMC is water-insoluble in contrast to conventional NaCMC-based hydrogels, it is highly water-swellable to form superabsorbent hydrogels. Carboxymethyl cellulose used in the hydrogels has increased the pore sizes because of the repulsion caused by the electronic cloud of carboxyl groups which produce to a greater swelling ratio [186,314]. The most common use of CMC in the electrochemical field is as a binder in electrodes [302].

Dueramae et al. [186] blended CMC sodium salt and Poly(N-isopropylacrylamide) (PNiPAM) a well-known thermo-responsive polymer, the properties of which can be fine-tuned at 32 °C, a temperature close to that of the human body, via the switchable hydrophilic amide group (–CONH–) and hydrophobic propyl [–CH(CH_3_)_2_] moieties in the monomer structure. PNiPAM was synthesized by free radical polymerization using benzene as solvent. The CMC/PNiPAMx-based SPEs (where x is the wt.% of PNiPAM) were fabricated by solution casting. The CMC was dissolved in deionized, the PNiPAM was added and dissolved in the CMC solution to the desired weight ratio (0–40 wt.%). ZnTf_2_ was used as salt resulting in the high Zn^2+^ ion transference number (0.56) and ionic conductivity (1.68∙10^–4^ Scm^−1^). Apart from the good results obtained, the synthesis process will have to be redefined to avoid the use of benzene.

**Figure 18:**
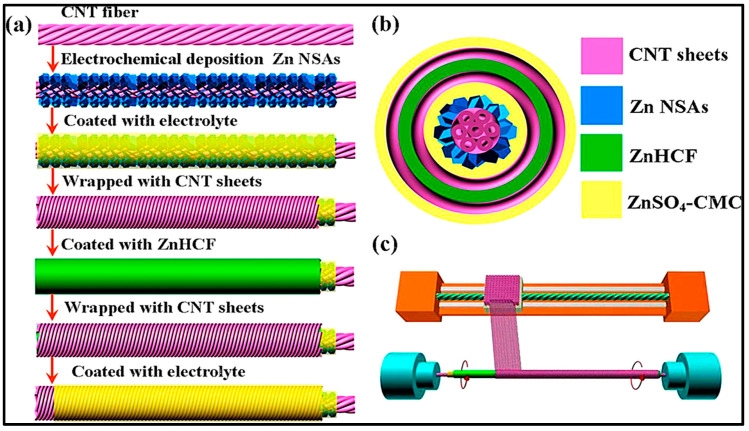
(**a**) Schematic illustrations showing the fabrication process of the coaxial aqueous rechargeable ZIB. (**b**) Schematic illustration of the cross-section view of the coaxial aqueous rechargeable ZIB. (**c**) Wrapping CNTSs around the modified CNTF. Reproduced with permission from [315]. Copyright 2019 The American Chemical Society.

A very interesting approach to develop fiber architecture suitable to be integrated in textiles is the one followed by Zhang et al. [315]. Unlike from the twin-fiber architecture, the single coaxial- fiber architecture offers a simple yet effective path for developing ultraflexible and small-size energy storage device avoiding the risk of fibers coming apart with movement or strains once integrated in fiber textiles, Figure 18. So far, it has been showed mainly in zinc-air and Zinc/MnO_2_ batteries. Although this work relies on the Zinc-hexacyanoferrate chemistry, it has been included here, as it describes a GPE made of CMC, which is a biobased polymer. Besides, in this section, it is intended to be only a paragon example for the yet to come massive research on single coaxial architecture. The discharge capacities maintain 100.2 mAhcm^−3^ at a current density of 0.1 Acm^−3^ and 66.5 mAhcm^−3^ at a high current density of 1 Acm^−3^. Energy and power density were 195.4 mWhcm^−3^ at a power density of 0.2 Wcm^−3^, and still deliver 126.9 mWhcm^−3^ even at a high power density of 1.9 Wcm^−3^—The battery was subsequently knitted in a textile powering a led.

#### 3.6.3. Gelatine

**Figure 19:**
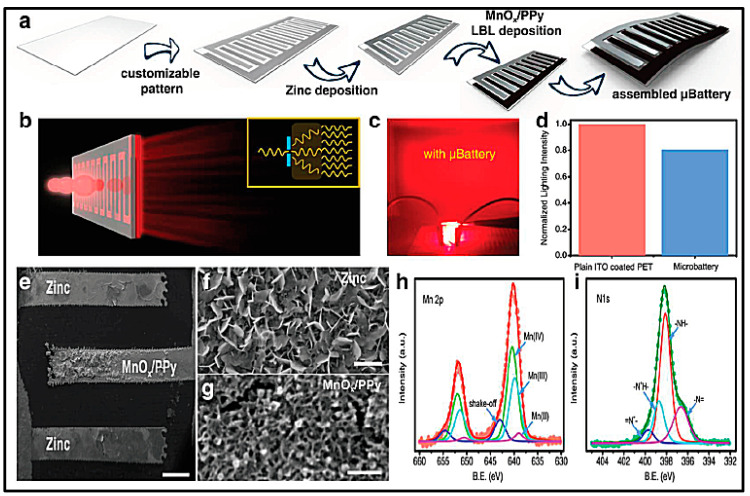
Fabrication and characterizations of the microbattery. (**a**) Schematic illustration of the fabrication process for the microbattery. (**b**) Schematic illustration of the light permeability and hazing effect of the microbattery. (**c**) The digital photograph with a red LEDs as the backlight, showing good light permeability and hazing ability. (**d**) Light intensity after permeating the microbattery, normalized to the light intensity after permeating the plain ITO coated PET. (**e**) SEM image of the interdigitated micro-electrodes (scale bar: 500 μm), (**f**) SEM images of the electrodeposited zinc anode. (**g**) SEM images of the electrodeposited MnOx/PPy cathode (scale bar: 500 nm). (**h**) Mn 2p core spectrum and (**i**) N 1s core spectrum of PPy measured by XPS. Reproduced with permission from [317]. Copyright 2018 Royal Society of Chemistry.

Gelatine is a polypeptide consisting mostly of proline, hydroxyproline and glycine amino acids extracted from denatured collagen (Figure 19). It dissolves in boiling water to form a pale yellow, semi-transparent, viscous solution. On cooling below 30–35 °C a solution of gelatine (minimun 2% w/v) forms a reversible physical hydrogel by intermolecular hydrogen bonds between large fractions of gelatine chains. This non-covalent associations are easily broken if Tª is risen again. This labile behavior explains why the utility of such a hydrogel is so limited due to its low network rigidity. To increase mechanical strength usually a chemical crosslinker or other crosslinking method are used. One special way of increasing its strength is by simply removing the natural occurring divalent ions, such Ca, Fe, Cu, etc., within the gelatine which disrupt the electrostatic interactions between the carboxylic acid groups and amine groups. Removing these divalent cations will enhance the electrostatic interactions carboxylic acid groups and amine groups hence increasing mechanical strength and stability of the gelatine hydrogel [316].

Han et al. [87]. argued that the key to enabling Zn metal-based flexible aqueous batteries is to develop a stable and durable electrolyte with excellent stability toward metal anodes. Quasi-solid-state electrolytes based on PVA, xanthan gum, etc., show high conductivity; however, they lack mechanical strength. Others suffer from high cost or complicated preparation. They presented a flexible gelatine hydrogel electrolyte (GHE) for rechargeable solid-state Zn metal batteries with high safety, taking advantage of the unique thermo-reversibility of the gelatine. The GHE can reversibly melts and gelates above and below the gelation temperature (*T*_g_) of about 38.4 °C. The 10 mm thick, interdigitated Zn//GHE//LMO battery used an absorbent glass mat (AGM) separator impregnated with an aqueous electrolyte (0.5 M Li_2_SO_4_ and 0.5 M ZnSO_4_) together with a gelatine electrolyte based on the same salts. The solid-state Zn//GHE//LMO battery possesses decent capacity, 104.4 mAhg^−1^ at 100 mAg^−1^ based on the mass of LMO (LiMn_2_O_4_).

Wang et al. [183] used also gelatine as biopolymer but crosslinked with borax. They used as support for anode and cathode Nitinol wire and SS fibers respectively. The zinc-ion battery Nitinol/Zn//GPE//PPy-MnO_2_/SS was tested also in aqueous electrolyte 1 mol L^−1^ ZnSO_4_ and 0.1 mol L^−1^ MnSO_4_ and compared with the electrochemical performance of the gelatine-borax-based electrolyte, the former delivering comparable electrochemical performance, demonstrating attractive properties as electrolyte material. The most remarkable feature of the battery was, the flexible battery could effectively recover to its original state maintaining most of the electrochemical performance for multiple times thanks to the shape memory effect provided by the Nitinol wire.

A very interesting application of flexible batteries with GPEs polymer biobased is presented by Zhu et al. [317]. They produced a photoluminescent microbattery array embedded in a battery-in-screen configuration, i.e., the batteries are integrated into a highly elaborate screen, Figure 19. The battery fulfills two function within the screen, namely as power source and as color filter due to tunable emission wavelength, narrow emission spectra and high luminescent efficiency properties of the added quantum dots (QDs). Borax was added to the gel polymer electrolyte (gelatine/1 M ZnSO_4_/QDs) to diminish the luminescence quenching of the QDs due to the high concentration of Zn Ions into the electrolyte. In addition to preventing luminescence quenching of QDs, the addition of borax also improves the ionic conducting performance of gelatine-based electrolyte, increasing by 27%. Thus, the semitransparent aqueous Zn-MnO_2_/polypyrrole-based microbattery (μZMB) based on an interdigitated architecture of electrodes and transparent electrolyte can be integrated in higher elaborated devices reducing two volume-consuming components.

**Figure 20:**
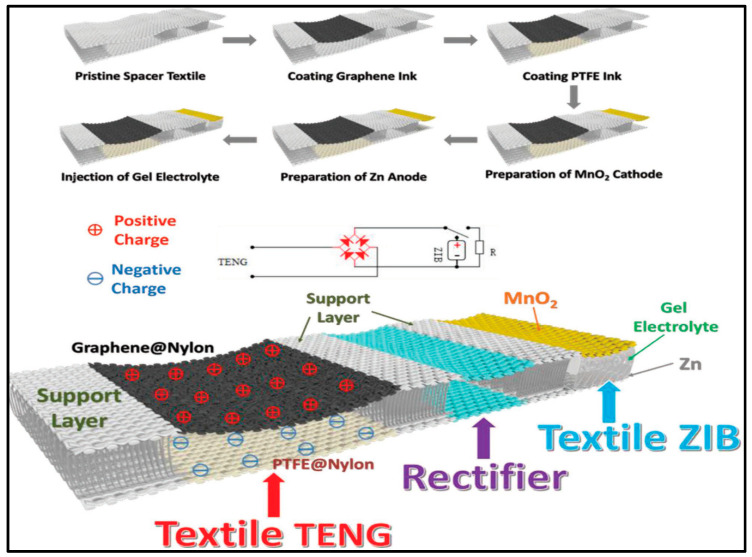
Fabrication protocol and the schematic diagram of the 3D spacer fabric-based energy harvesting and storage device. Reproduced with permission from [318]. Copyright 2018 Wiley.

In another interesting proof-of-concept, Wang et al. [318] developed an integrated electrochemical devise made up of a zinc-ion gelatine-based battery and a tribolectric nanogenerator (TENG). Friction between materials with different dielectric constants, would lead to the occurrence of triboelectrification on contacting surfaces converting mechanical energy into electricity [319]. Spacer fabrics are a kind of 3D manufactured textile structures in which two outer fabric layers are connected by a layer of pile threads. A defined fixed distance can be set between the two outer layers, ranging from 1.5 to 10 mm. The construction design affects the functionality of the 3D structure in terms of thermoregulation, breathability, pressure stability and pressure elasticity. In a 3D spacer fabric, the upper and lower layers serve as flexible substrate for electrode materials whereas the space in between is suitable for filling the gel electrolyte. Thus, Zn nanorod 10 µm long were electrodeposited on carbon cloth, MnO_2_ nanorods with PVdF and acetylene black were pasted, as slurry, on a carbon cloth and both of then attached to the two sides of the 3D spacer and gelatine-based gel electrolyte containing 1.0 M ZnSO_4_ and 0.1 M MnSO_4_ was introduced between them to form the Zn-Ion battery. Graphene and PTFE inks were spread on the two sides of the same 3D fabric but wide apart from the battery section (Figure 20), to form the TENG (Triboelectric nanogenerator). The whole assembly has three parts using a common 3D spacer fabric as shown in the figure. A rectifier circuit was intercalated between the battery and the TENG to control the charging of the battery. The battery was charged by simply pressing on the space fabric section where the TENG was placed.

Inspired by the high performance of the textile flexible electrodes, Zhao et al. [320] fabricated Zn-MnO_2_ batteries based on the self-branched MnO_2_ textile cathodes, and Zn nanosheet textile anode with gelatine/PAM as GPE. Self-branched hierarchical nanostructures ensure the fast electron and ion transport, and are favorable to high mass loading. Accordingly, it synergistically improves the energy storage and high rate capability. Thus, the flexible self-branched textile Zn-MnO_2_ batteries reached the maximum volumetric energy density of 12 mWhcm^−3^ at 13 mWcm^−3^.

With thicker gel polymer electrolyte a flexible cable-type zinc-air battery was developed by Park et al. [184] using spiral zinc anode with a free-standing GPE (gelatine and 0.1 M KOH), and air electrode with non-precious metal catalyst. Not only gel polymer electrolyte was fabricated using a biopolymer. The catalyst used silk as a source of N and C together with iron acetylacetonate as iron source. After pyrolysis, the Fe/N/C-900 catalyst with high catalytic activity for ORR was obtained.

Other authors explored the use of V_2_O_5_ as intercalation cathode for flexible zinc batteries; hence the use of gel polymer electrolytes is almost compulsory. Zhao et al. [88]. obtained layer-expanded V_2_O_5_·2.2H_2_O (E-VO) nanosheets which show large interlayer distance, fast charge transfer kinetics, and high structural stability to accommodate zinc ions in a sandwiched Zn//GPE//V_2_O_5_ (E-VO) battery with a gelatine-based GPE. The anode was fabricated by calendaring zinc foil onto SS mesh that produced much better performance that the bare zinc foil anode. Likewise, using a gelatine-based GPE, have proceed Wan et al. [86] but using a Vanadate cathode NaV_3_O_8_, with interlayer distance (0.708 nm), this would be large enough to enable the insertion/extraction of Zn^2+^ (0.074 nm), and H^+^ could steadily exist between the V_3_O_8_ layers. The nanostructured NaV_3_O_8_ would be an ideal positive electrode of aqueous rechargeable ZIB as it is able to have simultaneous H^+^ and Zn^2+^ insertion/extraction process. The simultaneous insertion extraction have synergistic effects according to the authors, dual carriers, and should be a guide for further developing new appropriate host materials for aqueous metal-ion batteries with high performance.

#### 3.6.4. Xantan Gum

**Figure 21:**
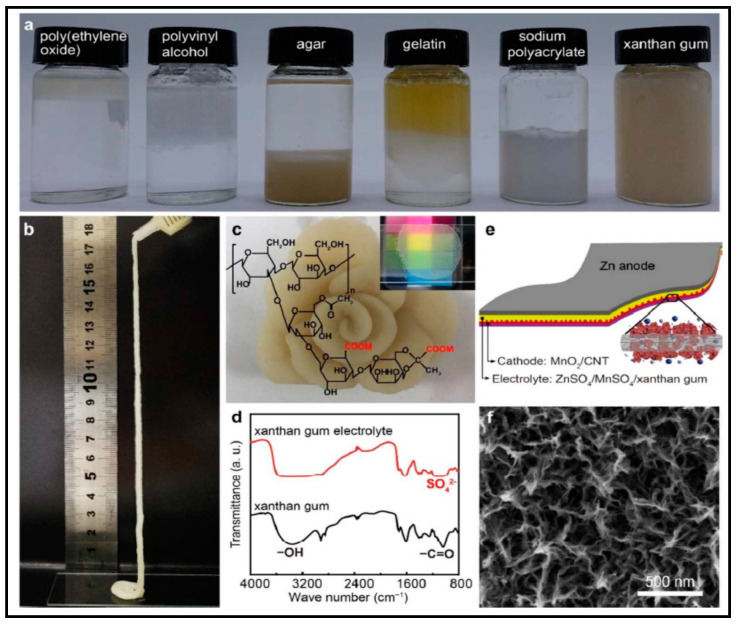
(**a**) Photograph of aqueous solutions of 2 M ZnSO_4_ and 0.1 MnSO_4_ after adding 10 wt.% polymers of poly(ethylene oxide) (PEO), polyvinyl alcohol (PVA), agar, gelatine, sodium polyacrylate and xanthan gum showing that PEO, PVA, agar, gelatine and sodium polyacrylate were aggregated while xanthan gum was uniformly dissolved in the solution. (**b**) Photograph showing that the sticky xanthan gum electrolyte could extruded from a syringe and self-support at a long length. (**c**) A hand-made flower using the xanthan gum electrolyte, the molecular formula of xanthan gum floating on the flower, and the top-right photo showing that the xanthan gum electrolyte can be readily shaped into a 10-μm-thick film. (**d**) IR spectra of xanthan gum and the xanthan gum electrolyte. (**e**) Schematic showing the structure of our gum Zn–MnO_2_ battery, which consists of a zinc foil as the anode, a ZnSO_4_/MnSO_4_/xanthan gum film as the separator and the electrolyte, and a MnO_2_/CNT film as the cathode, respectively. The pop-up shows the structure of the MnO_2_/CNT electrode, where a porous MnO_2_ layer is coated on a CNT thin-film. (**f**) SEM image of the MnO_2_/CNT hybrid film. Reproduced with permission from [64]. Copyright 2018 Royal Society of Chemistry.

Xanthan gum is an anionic polysaccharide (1,4-linked β-D-glucose backbone with trisaccharide side chain), which is the product of Xanthomonas campestris (aerobic bacteria) fermentation of sugars. Xanthan gum, like other carbohydrates, are strongly water absorptive and may dissolve in water to give highly viscous solutions Physical crosslinking can be done to obtain xanthan hydrogels by freeze thawing, ionotropic gelation, and polyelectrolyte complexation. Xanthan gum is considered an anionic polyelectrolyte due to the presence in its side chains of glucuronic acid and pyruvate, and for this reason, it is possible to form PEC (Polyelectrolytes complex) with polycations such as chitosan (Figure 21). A physical xanthan gel can be obtained by simple methods through incorporation of montmorillonite [321], KOH or HCl [66]. Xanthan gum was used as GPE for other electrochemical systems such Al-air [62].

Zhang et al. [64] developed a rechargeable adaptive and stable bio-electrolyte for rechargeable Zn-ion batteries Zn//GPE//MnO_2_/CNT using a high-salt-tolerant, water-soluble polysaccharide xanthan gum. Zn–MnO_2_ batteries were assembled using such gum electrolyte as both electrolyte and separator. The salt used, ZnSO_4_/MnSO_4_, according to the authors, causes serious precipitation to many polymers used for gel electrolytes, PEO, PVA, agar, gelatine, and sodium polyacrylate, whereas for xanthan gum it form very stable and uniform gum electrolyte. The ionic conductivity of the GPE (3 M ZnSO_4_/0.1 M MnSO_4_/20 wt.% xanthan gum) was 1.65∙10^−2^ Scm^−1^ using an AC impedance method. When compared to the liquid version of the battery, this showed a high discharge capacity of about 260 mAhg^−1^, based on MnO_2_ weight at 1 C, whereas the liquid one gave a relatively higher capacity of about 295 mAhg^−1^. They also found that the use of a xanthan gum electrolyte could significantly inhibit the growth of Zn dendrites as well as slow down the self-corrosion.

#### 3.6.5. Carrageenan

Carrageenan is the general term for linear sulfonated polysaccharide extracted from red seaweed (Rhodophyta), such as Kappaphycus alvarezii. These natural polymers comprise of repeating units of (1,3)-D-galactopyranose and (1,4)-3,6-anhydro-α-D-galactopyranose with sulfate groups in a certain amount and position. κ-carrageenan (one sulfate per disaccharide), λ-carrageenan (three sulfates per disaccharide), and ι-carrageenan (two sulfates per disaccharide) showed different structural features regarding the number and position of sulfate groups in the repeating unit. Carrageenan is hydrophilic in nature due to the presence of hydroxyl groups and the mentioned sulfate groups in it.

On an attempt to investigate and further minimize the environmental impact as well as manufacturing costs, Huang et al. [185] proposed the use of κ-carrageenan as GPE with ZnSO_4_/MnSO_4_, in highly flexible ZIBs with MnO_2_-based hybrid cathodes. However, the GPE hat to be reinforced with rice paper to improve its robustness. The battery can deliver discharging capacity of 291.5 mAhg^−1^ at 0.15 Ag^−1^ and high discharge capability of 120.0 mAhg^−1^ at a high current density of 6.0 Ag^−1^, based on MnO_2_ mass. They compared their results with another three different GPE-based batteries with similar results [64,142,218]. Same authors screened the use of guar gum gel polymer electrolyte in another work to be implemented in ZIB batteries as well [63].

#### 3.6.6. Chitosan

As one typical positively charged polysaccharide (Table 4), chitosan is derived from the deacetylation of chitin, which is the second most abundant natural biopolymer after cellulose, and is also under scrutiny to be used in batteries, for instance, for all-vanadium redox flow batteries (VRFBs) [322]. The positively charged groups of chitosan, come from protonation of its free amino groups, which is the key to its water solubility. Chitosan in aqueous acid solution reacted with anionic polysaccharides such as: carboxymethyl cellulose, xanthan, alginate, carrageenan, gellan, as well as synthetic polyanions such as poly(acrylic acid) to give polyelectrolyte complexes.

Kadir et al. [187] used a PVA–chitosan, 36 wt.% PVA and 24 wt.% chitosan, blend doped with 40 wt.% NH_4_NO_3_ and plasticized with ethylene carbonate (EC) to enhance the conductivity. The highest conductivity obtained was 1.60∙10^−3^ Scm^−1^ for EC concentration of 70% wt. A battery Zn(ZnSO_4_·7H_2_O)//GPE//MnO_2_ at a constant current of 2 mA was tested but adding liquid electrolyte to the cathode for comparison purposes. Very modest galvanostatic discharge were obtained.

Wei et al. [188] used an alkaline exchange membrane electrolyte, CS-PDDA-OH^−^ comprised of chitosan (CS) and poly(diallyldimethylammonium chloride) (PDDA) with high OH^−^ conductivity (0.024 Scm^−1^), strong alkaline stability, good thermal stability, and low degree of anisotropic swelling, was found to provide high electrochemical performance in fuel cell, supercapacitor and zinc-air battery. For what concerns to this work, zinc-based batteries, the CS-PDDA-OH^−^ membrane (PDDA 33%) as an effective separator and electrolyte in a rechargeable solid-state zinc-air exhibits a high open-circuit voltage of 1.3 V and peak power density of 48.9 mW cm^−2^, which is superior to the commercial A201 membrane-based cell (41.4 mW cm^−2^). The excellent performance of the CS-PDDA-OH^−^ membrane is due to its smaller anisotropic swelling and higher water uptake.

This is the first paper we come across where the same GPE is used in different electrochemical devices in the same work. This a proof of the versatility of biobased molecules that in the end, will produce a vast number of research items to probe its feasibility in any kind of electrochemical devices. It is surprising that one compound, despite its recent interest, has become one of the most studied polysaccharides for electrochemical devices. At this point, we have to recall that we have already mentioned some other polysaccharides such as Locust bean gum, tamarind gum, tara gum, gellan gum, dextran, Arabic gum, etc., which have not been much used or studied either, what it can lead us to imagine the possibilities ahead.

Biswal et al. [323] in a different way of using chitosan, managed to create a structure on the cathode which influences the nucleation and growth of the EMD during electrodeposition. EMD cathodes obtained in the presence of chitosan (1 g/L) and glutaraldehyde (1% glutaraldehyde) exhibited a reversible and better discharge capacity upon cycling than the blank which showed its typical capacity fade behavior with cycling. Chitosan did not play the typical role of GPE, strictly speaking, but at some extent it does in the cathode stabilizing the cathode and extending cycle life. The long-term cycling performance of this material was evaluated in Zn-MnO_2_.The authors also demonstrated that the positive effect showed by the crosslinked chitosan turned into no effect when chitosan was not crosslinked.

#### 3.6.7. Guar Gum

**Figure 22:**
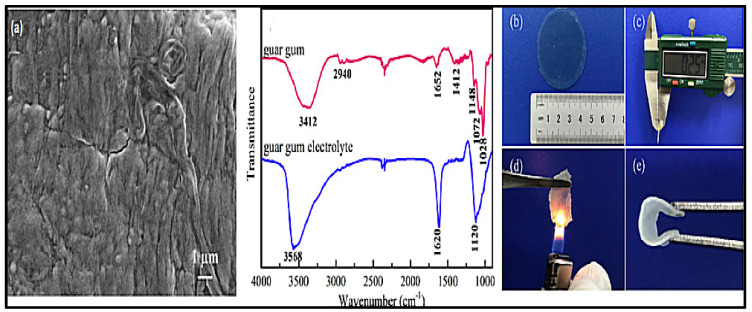
(**a**) SEM image of the guar gum electrolyte and FTIR spectra of guar gum and guar gum electrolyte. (**b**) Photograph of the guar gum electrolyte. (**c**) 0.25 mm thick guar gum electrolyte with 2 M ZnSO_4_ and 0.1 M MnSO_4_. (**d**) Flammability tests on the guar gum electrolyte. (**e**) Photograph of a folded guar gum electrolyte sample. Reproduced with permission from [63]. Copyright 2019 Elsevier.

Guar gum is a hydrophilic and nonionic polysaccharide derived from the seeds of the plant Cyamopsis tetragonolobus, Chemically, guar gum has a linear chain of (1→4)-linked β-D-mannopyranosyl units with (1→6)-linked α-D-galactopyranosyl residues as side chains with mannose: galactose ratio is approximately 2:1. (Figure 22) The guar gum also absorbs large quantities of water, resulting in dispersions of extremely high viscosity. However, due to uncontrollable rate of viscosity, uncontrollable rate of hydration and instability of its solution for a long time restricts its use [314].

Huang et al. [63] used guar gum to form free-standing solid-state ZnSO_4_/MnSO_4_ electrolyte. The fabricated flexible ZIBs with guar gum electrolyte showed several merits: high capacity (308.2 mAhg^−1^ at 0.3 Ag^−1^) and high rate capability (131.6 mAhg^−1^ at 6.0 Ag^−1^). The highest ionic conductivity at RT was 1.07∙10^−2^ Scm^−1^ for a GPE with 2 M ZnSO_4_ and 0.1 M MnSO_4_. The authors claimed this one to be allegedly one of the highest conductivity and provided a comparative table as supporting information. The reaction mechanism of the MnO_2_ cathode with the guar gum electrolyte, by ex situ XRD on the MnO_2_ electrode after discharge to 1.0 and charged to 1.9 V. showed typical ZnMn_2_O_4_ and MnOOH peaks. The presence of the ZnMn_2_O_4_ and MnOOH peak strongly supports the mechanism that the α-MnO_2_ cathode experiences Zn^2+^ and H^+^ insertions. Finally, they assembled a solid-state ZIBs with guar gum where (MnO_2_/rGO) composite electrode on carbon cloth was used as cathode and electrodeposited Zn on carbon cloth as anode. They also gathered in a table, provided in the Supporting Information file, comparative information regarding conductivity of different gel polymer electrolytes as well as the performance of the batteries in which the GPEs were fitted to.

Guar hydroxypropyltrimonium chloride is a biodegradable water-soluble quaternary ammonium derivative of guar used mainly in cosmetics. Despite this, Wang et al. [189] tried to use it as GPE. Excellent hydroxide ion conductivity plays a vital role in the performance of AAEM. Improvement of the hydroxide ion conductivity, it is at the same time accompanied by a serious side effect: the consequent excess of water uptake will result in a considerable swelling ratio, leading to the progressive loss of dimensional stability. They proposed a highly conductive AAEM with binary cross-linking strategy using glutaraldehyde (GA) and pyrrole-2-carboxaldehyde (PCL) as binary cross-linking agents to. The resulting, PGG-GP called, membrane showed an ionic conductivity of 0.123 Scm^−1^ at RT. The AEEM was used in both supercapacitator and zinc-air batteries. Water-retaining ability is a key factor affecting cycling stability of zinc-air batteries. Thus, loss and gain of water give rise to wrinkling of the membrane, which cause poor contact between the membrane and the electrode. The PGG-GP membrane exhibited a water uptake of more than 3-fold of A201 membrane, whereas its anisotropic swelling degree was smaller than that of A201 one. Consequently, the zinc-air battery using the PGG-GP membrane displayed better stability performance.

#### 3.6.8. Sodium Alginate

Alginate, water-soluble sodium salt of alginic acid, is an anionic natural polymer that can be extracted from renewable resources such as brown seaweed with mannuronic and glucuronic acid units (Table 4). Alginate has been used in medical applications due to its biocompatibility, low toxicity, and rapid gelation in the presence of divalent cations, commonly Ca^2+^, which form ionic bridges between alginate chains forming the so-called “egg-box”. This gelation can be observed at room temperature and physiological pH. Alginate contains hydroxyl groups and carboxylic groups can take part in multiple chemical reactions to modify the characteristics of the alginate. As biobased material, and presumably harmless, the main use of alginate is related to biomedical applications; however researchers have tried also use it in GPEs.

Thus, Lu et al. [190] developed a three-dimensional, double cross-linked gelatine and sodium alginate hydrogel, ZnSO_4_ aqueous solution embedded, for flexible Zn-ion batteries. First gelatine is cross-linked with glutaraldehide and then sodium alginate is, gelated with CaCl_2_ everything through a one pot synthesis. Membrane is then soaked to get the gelatine and alginate-based membrane electrolyte (denoted as GAME). The mass ratio of GE and SA in GAME was optimized based on mechanical strength, phase composition, and ionic conductivity. GAME with mass ratio of GE/SA = 1/1 showed the maximum ionic conductivity of 3.7∙10^−2^ Scm^−1^, hence this composition was selected for further electrochemical tests in a practical ZIB in which GPE was assembled with V_2_O_5_ nanowires/CNT as cathode and graphite paper loaded with Zn, prepared by the electrodeposition, as anode. Thus, the V_2_O_5_ nanowires/CNT//GPE//Zn/graphite foil battery showed good cyclic stability at 2.0 Ag^−1^, delivering a capacity of 251 mAhg^−1^ at the first cycle and 188 mAhg^−1^ after 200 cycles and a Coulombic efficiency > 99.8%.

Military needs and space exploration spurred much of the first research and development to improve batteries, which had been in use for decades. This role seems now to be taken over by biomedicine. The importance of flexible batteries to the biomedical field is simply patent when specific biomedical implants need a power source to perform sensing or stimulation functions that can influence critical biological processes such as wound healing, tissue regeneration or brain activity. These power sources become then the cornerstone allowing sensing or stimulation by an actuator whose data or result will trigger the subsequent research. This the case of a fully biodegradable batteries for self-powered temporary or transient biomedical implants as the one presented by Huang at al. [324] by introducing dissolvable bio compatible or biodegradable metals and oxides and polymers. The authors proposed a high-performance fully biodegradable primary magnesium–molybdenum trioxide (Mg–MoO_3_). Mg serves as the anode material and MoO_3_ as cathode using an alginate hydrogel phosphate doped electrolyte. The battery could satisfy most of the ultralow-power implantable devices as well as maintain robust functions, as the required voltage and power are typically in the range of ≈0.5–1.6 V and ≈10–1000 µW. So far, we have not found any work devoted to the use of zinc in bioresorbable electronics or self-deployable power sources but the perspectives are optimistic [325,326].

Another interesting work, outlining one more time the relation of zinc-based batteries with biomedical research, is the one done by Majdecka et al. [327] with the oxygen reduction reaction active enzyme Laccase *Cerrena unicolor*. These last two research works by Huang at al. and Majdecka et al. should be considered differently here, as outliers, which will be too soon ordinary or run-of-the-mill works done on zinc-based batteries.

### 3.7. Synthetic Biodegradable Polymers

Biodegradable polymers degrade in vivo, either by enzymatic or non-enzymatic means and transform into biocompatible, innocuous, or nontoxic products. A More formal definition for biodegradable: “Capable of undergoing decomposition into carbon dioxide, methane, water, inorganic compounds, or biomass in which the predominant mechanism is the enzymatic action of microorganisms that can be measured by standardized tests, in a specified period of time reflecting available disposal conditions” is the one given by the ASTM organization [328]. We have to keep in mind that PVA is a biodegradable polymer but, due to the fact it is the most important polymer concerning the GPEs field, has been treated separately.

Among them, PLA (polylactic acid), poly(ε-caprolactone), polyphosphazene, Poly(hydroxyl butyrate), polyanhydride, poly(lactide-co-glycolide) were already used as GPEs in lithium. There is a myriad of polymers belonging to this category that were not been yet explored as GPEs, hence the research possibilities ahead are enormous [329]. The research on zinc-based batteries using the just mentioned polymers lags far behind the one done on lithium-based batteries but it is expected soon to hit these polymers. However, there are some examples of the research done on these polymers, apart from PVA, for zinc-based batteries. Thus, Sownthari and Suthanthiraraj [191] in 2013, in an early attempt to replace more harmful polymers tried to use poly ε-caprolactone in combination with zinc triflate ZnTf_2_ in different weight percentages but the electrolyte is more a SPE than a GPE. Nevertheless, in this study a full discharge of Zn//Polymer electrolyte//MnO_2_ battery with a constant load of 1 MΩ at room temperature (25 °C) was achieved. More recently, the same authors studied the same electrochemical system to which an organically modified montmorillonite was added. Poly(ε-caprolactone) has been also used to fabricated biodegradable batteries for temporary implant similar to the one reported with sodium alginate by Huang et al. [324], in this case the battery is based on Mg/iron [330].

### 3.8. Others Polymer Electrolytes

PAN(polyacrylonitrile) has outstanding properties, such as high thermal stability, high ionic conductivity, good compatibility with lithium electrodes, a well-formed morphology for electrolyte absorption, and the ability to minimize dendritic formation during the charge and discharge processes [331]. However, its use is scarce in zinc-based batteries.

One of the first works with PAN as GPE in zinc-based batteries was done by Kumar and Sanpath [192]. They fabricated a GPE based on polyacrylonitrile (PAN), propylene carbonate (PC), ethylene carbonate (EC), and zinc trifluoromethane sulfonate ZnTf_2_ as salt. The maximum conductivity, 2.61∙10^−3^ Scm^−1^ was achieved for a GPE with a PAN:PC:EC:ZnTf mass ratio of 1:2.4:2.4:2. The reversible electrochemistry observed in CV for the zinc electrode spurred them to assemble several Zn/GPE/γ-MnO_2_ which were able to achieve 70 cycles with no significant changes in the discharge behavior. It is worth reviewing the work done by Zhang et al. [332]. They presented a hybrid battery where when charging takes place, sodium ions are removed from Na_4_Mn_9_O_18_ cathode and dissolved in the electrolyte, releasing electrons, and zinc cations are deposited from the electrolyte on the surface of the zinc anode. During the discharge process, Zn is oxidized and dissolved into the electrolyte and sodium ions are intercalated into the cathode to reversibly form Na_4_Mn_9_O_18_. PAN-nanofibres membrane with high capability to absorb liquid electrolyte (65 wt.%) was soaked in 1 M Na_2_SO_4_ and 0.5 M ZnSO_4_ and used as GPE and separator. The GPE-based battery reached a capacity of 88 mAhg^−1^ at 1C rate.

Fumed silica has proved to be an effective thickening agent in aqueous, organic and in aqueous-organic mixtures. It has relevant industrial interest in pharmaceuticals, cosmetics, foods, ceramics, electronics, etc. applications. The branched colloidal microstructure of fumed silica with large specific surface area and surface hydrophilicity is the reason for the extensive use of fumed silica as a thickening and gelling agent [193,333]. Thus, Chao et al. [194] used fumed silica/ZnSO_4_ (conductivity ≈8.1 mScm^–1^ at RT) electrolyte in a quasi-solid-state zinc battery (QSS). The anode was Zn nanoflakes array on a 3D porous conductive and zinc orthovanadates as cathode supported in graphene foam as well. The zinc orthovanadate was indexed to orthorhombic Zn_2_(OH)VO_4_, demonstrating the (de)intercalation trough the following solid-solution reaction mechanism without phase transformation Zn_2_(OH)VO_4_ + Zn^2+^ + 2e^−^ ↔ Zn_3_(OH)VO_4_. The QSS battery exhibits a maximum energy density of ≈140 WhKg^−1^ (with power density 70 WKg^−1^) and a maximum power density of 6200 WKg^−1^ (with energy density 65 WhKg^−1^) based on the total active material mass of cathode and anode.

Prasanna et al. [334] used a nanocomposite gel polymer electrolytes (NCGPEs) comprising of salt, 1-ethyl-3-methylimidazolium bis(trifluoromethylsulfonyl)imide (EMIMTFSI) ionic liquid (IL) and fumed silica (PVC/PEMA poly(ethyl methacrylate) blend, zinc triflate Zn(Tf)_2_, SiO_2_ as thickening agent. Maximum conductivity for this gel was achieved for 3 wt.% SiO_2_ (6.71∙10^−4^ Scm^−1^). They used PVC since it is a cheap and available material but exhibits poor thermal stability owing to its degrading nature above the glass transition temperature followed by elimination of HCl and formation of conjugated polyenes. They managed to solve this setback using PEMA as a scavenger of chlorine radicals as soon as they are formed. The addition of 3 wt.% fumed silica was found to be very effective in increasing the amorphous phase of PVC/PEMA blended polymer matrices doped with ZnTf_2_ and its consequent increase in conductivity. Finally, the establishment of high currents along with anodic and cathodic peaks by CV confirmed the reversibility of Zn/Zn^2+^ couple and its applicability in zinc-based batteries. In another step forward, the same authors studied the same electrochemical system replacing fumed silica by SnO_2_ and ZrO_2_ obtaining similar results. Yang et al. [335] used a composite polymer PVA/PVC, but PVC was considered to be filler since it cannot be dissolved in water, crosslinked with 5 wt.% glutaraldehyde (GA) and a 1 vol% HCl as catalyst.

Cellulose is associated with other natural occurring compounds, such as lignin and pectin. Thus, the lignocellulose materials are a mixture of biopolymers with cellulose intertwined (35–83%), hemicellulose (0–30%), lignin (1–43%) and some extra compounds. Lignin structure (p-coumaryl, coniferyl and sinapyl alcohols), heterogeneity and the industrial processing costs for delignification are the main reasons for the limited use of the lignin. However, it can be used as hydrogel mainly for biomedical applications [336,337].

Yuan et al. verified the formation of zinc hydroxide sulfate ZnSO_4_·[Zn(OH)_2_]_3_·xH_2_O, denominated ZHS, on Zn metal during stripping/plating in ZnSO_4_ using lignin/Nafion membranes obtained by casting solution, which were soaked in 2 M ZnSO_4_ at a later stage. Membranes with 10 and 20% weight lignin (NL10 and NL20) were compared to a normal Nafion membrane and ZnSO_4_ soaked filter papers. With 10 wt.% lignin, the cycle life can be further increased up to 410 h from the 345 h obtained using Nafion membranes.

The significance of ZHS being an effective SEI on Zn is discussed for rechargeable ZIBs. With lignin added, the water-deficient phase (ZnSO_4_·[Zn(OH)_2_]_3_·xH_2_O O/S ratio <13 indicates a water-deficient phase) showed a large increase in scattering intensity during cycling with NL10 as demonstrated by XRD. These results were correlated with the preferred growth of deposited Zn anodes. Thus, with filter paper, growth of Zn (0 0 2) was favored whereas no significant change was found for Zn (1 0 0). Suppression of Zn (0 0 2) growth was observed for Nafion during cycling and with NL10, both suppression of (0 0 2) growth and promotion of (1 0 0) growth were observed. Change of the interfacial resistance showed much better values for NL10 in the long term after an initial sharp rise and the cross-section views shows that except when using filter paper, no clear protrusion can be found on the Zn surface. The development of functional membranes able to reshape Zn^2+^ coordination highlights the significance of an effective SEI on Zn metal in mild electrolyte and open a feasible route to designing and fabricating effective SEIs for Zn metal toward high-performance rechargeable ZIB.

## 4. Summary and Perspectives

In the short term, lithium will continue to be the technology to overcome. This review has been gone through many gel polymer electrolytes systems, which have already been for lithium-based batteries studied used but could not find the same system for zinc-based batteries. A whole overview of gel polymer electrolytes was shown in this work, covering the most important and used polymer for creating these solid electrolytes. Furthermore, most of all zinc chemistries and gel polymers electrolytes either aqueous or non-aqueous have been reviewed. The wide-ranging catalog of polymers make us confident that GPE technology and technique are a step closer to deeper commercial deployment of wearable electronic gadgets, smart textiles and resorbable electronics.

So far, the driving force behind the use of gel polymer electrolytes in energy storage devices, according to the latest research available, seem to come for the interest of including them, due to its flexibility, as flexible power sources in wearable electronic devices. These wearable items, incorporating a flexible power source, could become fast-moving consumer electronics. One of the inherent features of fast-moving consumer goods is their short lifespan. Related to wearable electronics, due to the rapid change in electronic capabilities, theses will be discarded rather than repaired, hence worsening the electronic junk recycling problem. From the recycling point of view, LCA granted (Life Cycle Analysis), the ideal gel polymer electrolyte must be and aqueous one once all the electrochemical major drawback and disadvantages associated with them were overcome and, reiterating the recycling point of view, the most obvious choices would be PVA, PAM and PAA/PANa; cheap, safe, and available polymers, environmentally biodegradable with no reported adverse effects, with manufacturing processes, fully implemented and easy processing. However, none of the GPE host materials that this review has been trough can be ruled completely out as candidate for specific applications, for instance, alginate hydrogel phosphate for resorbable batteries. The same criteria applies to biobased gel polymer electrolytes including the most abundant polymer, cellulose, which has proven to be also a versatile material. We have seen that using bacteria cellulose, higher purity, would diminish the use of plant celluloses and therefore, the use of chemical for purification of plant celluloses would also diminish provided that cost of bacterial cellulose decreases in the next years.

However, these small wearable systems should be considered to be lab scale research for a more ambitious and crucial stationary and EV energy storage systems to come, in the near future that will regulate the intermittent energy output from renewable energy sources (e.g., wind and sunlight) and expand the autonomy range of the EV. All the expertise gained with these materials should be applied to bigger systems using liquids electrolytes, where the later may mean a risk to the operation or safety.

From an electrochemical point of view, electrochemically stable electrolyte/electrode interfaces are a prerequisite for achieving long-term cyclability. The solid electrolyte interface (SEI) exists and forms both at the interface of electrolyte/anode and electrolyte/cathode. The interfacial properties, such as charge transfer reactions at the interface, have a vital influence on performance in cycle life, Coulombic efficiency, and voltage efficiency. Generally, gel polymer electrolytes protect surface anode creating a stable SEI, which will benefits batteries performance. On the other hand, specifically engineered SEI has even showed higher protective behaviors.

A double decision has to be made with respect to the type of zinc battery and the gel which will form part of when envisaging electronic devices; both will have to fulfill all the required features such voltage, capacity, mechanical endurance, electrochemical compatibility, safety, cost, etc., when implemented in an electronic device.

Finally, yet importantly, direct comparisons between batteries performance are difficult to make successfully since tests are not usually done under the same operating conditions: cycling test parameters, the concentration of the aqueous electrolyte within the polymer and the molecular weight of the polymer are different. Besides, there is a big bundle of different types of bifunctional catalysts, in case of metal-air batteries, which lead to poor or inconsistent conclusions. Nonetheless, comparative tables, as the one presented here, are useful as a glimpse of the whole.

## Figures and Tables

**Table 1:** Comparison of different batteries. Data from references [20,21,22,23,24].

Batteries	Capacity (Whkg^−1^)	Capacity (Whl^−1^)	Life Cycles	Voltage (V)	Commercially Available Batteries [25,26,27]	
					Capacity (Whkg^−1^)	Capacity (Whl^−1^)
Ni-Cd	45–80	50–150	300–2000	1.2	48	122
Ni-MH	60–120	140–300	180–200	1.25	52	175
Lead-acid	33–42	60–110	200–300	2.1	25	85
Li-ion	160–300	250–693	400–1200	3.2–3.8	114–240	314–680
Zn-ion	80–120	>500	>1000	0.6–1.75	-	-
Zn-Ag	81–276	4–970	<100	1.6	163	308
Zn-MnO_2_	145	400	>500	1.5	163	398
Zn-Air	200–250	270	100–200	1.0–1.2	442	1673
Zn-Ni	70–100	280	500	1.7	75	170

**Table 2:** Common popular polymers used for making GPEs.

Polymer	Monomer	Glass Trans. Tª (°C) [129]	Crosslinking Methods
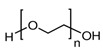 Polyethylene oxide (PEO)	–[CH_2_–CH_2_–O]_n_–	−64	Physical crosslinking: UV radiation.
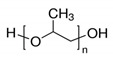 Polypropylene oxide (PPO)	–[CH_2_–CH(–CH_3_)O]_n_–	−60	Chemical crosslinking: Polysiloxane.
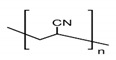 Polyacrylonitrile (PAN)	–[CH_2_–CH(–CN)]_n_–	109	Physical crosslinking: γ-rays radiation. Chemical crosslinking: Hydrazine
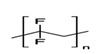 Polyvinylidenefluoride (PVdF)	–[CH_2_–CF_2_]_n_–	−34	Physical crosslinking: UV radiation. Chemical crosslinking: Diamines, dithioles, etc.
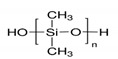 Poly(dimethylsiloxane) (PDMS)	–[(CH_3_)_2_–SiO)]_n_–	−127	Chemical crosslinking: pentaerythritol-derived.
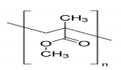 Poly(methylmethacrylate)	–[CH_2_C(–CH_3_)(–OOCH_3_)]_n_–	105	Chemical crosslinking: Ethyleneglycol dimethacrylate, 1,6-diaminohexane, etc.
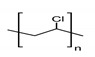 Poly(vinyl chloride) (PVC)	–(CH_2_CHCl)_n_–	83	Physical crosslinking: radiation. Chemical crosslinking: silanes, peroxides.
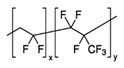 Poly(vinylidene fluoride-co- hexafluoropropylene) (PVdF-HFP)	–(–CH_2_CF_2_–)_x_–co– [–CF_2_CF(CF_3_)–]_y_–	−65	Physical crosslinking: electrospinning., radiation Chemical crosslinking: diamines, carbamates.peroxides, etc.
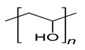 Poly(vinyl alcohol) (PVA)	–[CH_2_CH(OH)]_n_–	80	Physical crosslinking: freezing/thawing, H-bonding. Chemical crosslinking: GA, borax.
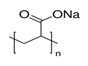 Sodium polyacrylate (PANa)	–(CH_2_CHCONH_2_)_n_–	-	Physical crosslinking: UV radiation. Chemical crosslinking: SiO_2,_ FeCl_3_.
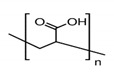 Polyacrilic acid (PAA)	–(C_3_H_4_O_2_)_n_–	101	Physical crosslinking: H-bonding. Chemical crosslinking: FeCl_3_, vinyl hybrid silica nanoparticles.
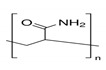 Polyacrylamide (PAM)	CH_2_=CHC(O)NH_2_–	165	Chemical crosslinking: (bisacrylamide, typically: *N*,*N*’-methylenebisacrylamide (MBA).
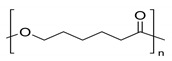 Poly(ε-caprolactone) (PCL)	–(C_6_H_10_O_2_)_n_–	−66	Physical crosslinking: radiation. Chemical crosslinking: peroxides.

**Table 3:** Summary of GPEs systems mentioned in this work.

Polymer Matrix	Salts	Salts Conc. sk. (Soaked)	Ionic Conductivity (S/cm)	Ta (°C)	Mechanical Strength (MPa)	Ref. No.	Battery Type	Remarks
PVDF-HFP	Zn(TFSI)_2_/ ZnTf_2_ + IL EMIMTFSI	0.2 M	1.31∙10^−3^	25	-	[138]	-	There is no visual difference between the membrane containing Zn salt and the membrane without Zn salt, which suggests homogeneous blending. Electrochemical stability window with anodic stability limit at 2.8 V vs. Zn^2+^/Zn and single phase behavior from −50 °C to 100 °C
PVDF-HFP	ZnTf_2_/ EMIMTf	30%	1.96∙10^−3^	30	-	[139]	Zn-MnO_2_	Inclusion of ILs inside the membranes is necessary to obtain GPEs with good electrical characteristics; poor results are obtained for IL-free GPEs. NMP residual is fundamental to rise the cation transport number
PVDF-HFP	ZnTf_2_/ TiO_2_	25%/ 5%	3.4∙10^−4^	25	-	[140]	Zn-ion	Cation transport due to the migration of Zn^2+^ ions has been enhanced, in the case of TiO_2_ up to 5 wt%, owing to the formation of a space charge region, which would provide an enhanced mobility of Zn^2+^
PVDF-HFP/PEO	Zn(BF_4_)_2/_ [EMIM]BF_4_	25%	16.9∙10^−3^	25	9.12	[141]	Zn-ion	Reversibility and stability of metallic Zn in ILZE cell under fixed galvanostatic condition shows a dense and dendrite-free morphology, which contains no zinc oxides or Hydroxides(>3000 cycles). The solid hydrogen free Zn/cobalt ferricyanide battery shows over 30,000 cycles with 90% capacity retained at a ≈100% columbic efficiency and can work at wide temperature range of −20 to 70 °C.
PVA	LiCl/ZnCl_2_/ MnSO_4_	3 M LiCl/ 2 M ZnCl_2_/ 0.4 M MnSO_4_	0.897∙10^−2^	/	/	[142]	Zn-MnO_2_	The protective layer of PEDOT as well as the mild neutral electrolyte suppress the structural pulverization and dissolution of MnO_2_ and help the rechargeable Zn–MnO_2_ battery to exhibits a favorable capacity retention
PVA	KOH	3 g	30.3∙10^−3^	25	-	[143]	Zn-air	Water plays a significant role in the transport of ions for the PVA–KOH GPE, which consists of a PVA polymer matrix and KOH aqueous electrolyte for mitigating ohmic polarization and promoting the resulting reaction kinetics
PVA	KOH	12 M(sk.)	0.34	20	-	[51]	Zn-air	The Grotthuss mechanism significantly contributes to or is the central mechanism for hydroxyl anion transport through PVA-KOH channels. Confinement of Zn^2+^ close to Zn electrode by GPE makes necessary the OH^–^ transport through the membrane as the only ionic species causing conductivity
PVA/TEAOH	KOH	18 M	30∙10^−3^	-	-	[144]	Zn-air	Tetraethylammonium hydroxide (TEAOH) replaces KOH as the ionic conductor in the TEAOH−PVA electrolyte. A greatly improved shelf life of the TEAOH−PVA electrolyte is achieved compared to normal PVA-KOH. The special NR4^+^ structure of TEAOH is relatively stable because the positive charge of the TEA^+^ remains the same regardless of the pH of the environment. TEAOH is hygroscopic which binds the water in the polymer electrolyte more tightly.
PVA	KOH	0.15 M	3.33∙10^–4^	-	-	[145]	Zn-air	Fiber-shaped zinc–air batteries are fabricated via a continuous method with a atomically thin mesoporous Co_3_O_4_ layers in situ coupled with N-rGO nanosheets as the bifunctional catalyst synthetized by high-yield method. The potential gap ∆E between E_j_ = 10 and E_1/2_ is generally used to assess the bifunctional activity of catalysts. The lower value of ∆E means better bifunctional activity.
PVA	KOH/ SiO_2_	6 M/5%	57.3∙10^−3^	25	-	[146]	Zn-air	Porous PVA-based nanocomposite GPE containing 5 wt% SiO_2_ exhibits good ionic conductivity and electrolyte retention capability as well as good thermal and mechanical properties. SiO_2_ contribute to the electrolyte retention property due to the presence of high levels of bound water in the GPE formed through SiO_2._
PVA/GA	KOH	2%	15∙10^−3^	-	-	[147]	Zn-air	Battery components can be prefabricated as sheets of customized shape and size to fit space and energy needs for a variety of applications.
PVA/PEO	KOH	8.3%	0.3	25	-	[148]	Zn-air	PEO was added to improve the mechanical properties of the electrolyte (0.83%). Aligned carbon nanotube (CNT) as sheets are materials that show conductivities of 10^2^–10^3^ S∙cm^−1^ and high tensile strengths in the order of 10^2^–10^3^ MPa. The number of CNT layer and the way CNT sheet electrode in rolled around hydrogel determines the performance of the battery.
PVA/PAA	KOH	32% (sk.)	0.301	RT	-	[149]	Zn-air	Ionic conductivity of the alkaline PVA/PAA polymer electrolyte membrane increased as the PAA content increased.
PVA/PAA/ NAFION	KOH	6 M (sk.)	6.6∙10^−3^	RT	39.4	[150]	Zn-air	Nafion, can be suggested as a potential single cation conductor suppressing Zn(OH)_4_^2−^ crossover without deteriorating OH- conduction. Bicontinuous phases provide synergistic effects of blends. Into porous regions of the intermolecular condensed PVA/PAA nanofibres mat, Nafion is impregnated as an anion-repelling phase.
PVA/PAA/GO (PVAA–GO)	KOH/ KI	4 M KOH/ 2 M KI (sk.)	0.155	-	67	[151]	Zn-air	I^−^ anions were employed as a soluble reaction modifier additive to reduce the charging potential alleviating degradation of the carbon-supported catalyst electrode. Hydrogen bonds of PVA have been partially replaced by hydrogen bonds among PVA, PAA, and GO.
PVA	KOH	-	10∙10^−3^	-	-	[152]	Zn/Ag	Cathode architecture with silver nanoparticle ink embedded into the conductive thread. Void spaces in the dendritic Zn deposit make it more flexible than compact Zn. batteries with 10 wt% KOH demonstrate stable capacity with lower silver migration.
PVA	Zn(CF_3_SO_3_)_2_	2 M	12∙10^−3^	-	-	[153]	Zn-ion	Freezing/thawing of PVA forms more crystalline microdomains, which serve as cross-links to achieve a porous network structure with pore size of 50–500 nm. When Zn(CF_3_SO_3_)_2_ is incorporated, gel fraction of free-moving PVA chain segments attract each other re-establishing hydrogen bonds and healing the fracture.
PAAM/ Zn-Alginate	ZnSO_4_/ MnSO_4_	2 M ZnSO_4_ + 0.1 M MnSO_4_	43∙10^−3^	-	-	[154]	Zn-MnO_2_	Dual-crosslinked energy-dissipative hydrogel electrolyte endows the battery with outstanding flexibility and exceptional stability. Under heavy stress the covalently crosslinked PAAm network is deformed but maintain shape and strength of the hydrogel, whereas alginate network breaks, leading to effective energy dissipation.
PAAM	ZnSO_4_/ CoSO_4_	2 M ZnSO_4_/ 2 M CoSO_4_	0.12	-	-	[155]	Zn/rich-Co_3_O_4_	Highly reversible conversion reaction in Zn/Co(III) rich-Co_3_O_4_ system in mild aqueous electrolyte. The freeze-dried PAM hydrogel possesses interconnected macro-pores which allows ions to transfer freely in the electrolyte allow. Hence fast kinetic in the process of charge-discharge. High content of Co(III) in the Co(III) rich-Co_3_O_4_ leads to highly effective redox reaction.
PAAM PANa	ZnSO_4_/ KOH-Zn(Ac)_2_	2 M ZnSO_4_/ 6 M KOH+ 0.2 M Zn(Ac)_2_	0.12/ ≈0.15	-	-	[156]	Zn-ion capacitor/Zn-air	U-shaped electrode is dual-functional with one part as a capacitor electrode and another part as an air electrode with two different electrolytes. The zinc-ion capacity maintains constant even overall 20,000 cycles. The high energy density of the zinc-air part could supply sufficient energy for zinc-ion capacitors.
PAM	KOH	6 M(sk.)	0.33	-	-	[157]	Zn-air	The highly crosslinked PAM film was also found compatible with aqueous saline neutral pH GPE for flexible aluminum-air (Al-air) batteries. The optimal crosslinker (MBA) concentration was 0.2 mol% Catalyst powder was just simply spread onto PAM to form a catalyst layer in the side air-cathode.
PAM	KOH	20%	0.215	-	-	[158]	Zn-air	The oxygen catalytic activity of MnO_2_/NRGO-Urea is much higher than that of MnO_2_/C and NRGO, suggesting the synergistic effect of MnO_2_ and NRGO
NFC/PAM	ZnSO_4_/ MnSO_4_	2 M ZnSO_4_/ 0.2 M MnSO_4_ (sk.)	22.8∙10^−3^	RT	-	[159]	Zn-MnO_2_	With the addition of cellulose (NFC), a much larger and stable porous structure is formed. Sewing, enhance the shear tolerance of the battery.
EG-WAPUA/ PAM	ZnSO_4_/ MnSO_4_	2 M ZnSO_4_/ 0.1 M MnSO_4_	14.6∙10^−3^	−20		[160]	Zn-MnO_2_	The alcohols molecules must be anchored onto the polymer chains through covalent bonds to form a stable unified matrix to get anti-freezing hydrogel electrolytes. Covalent cross-linking bonding and physical hydrogen-bonding endows the synthesized hydrogel with excellent flexibility. Water molecules connect hydroxyl groups of the EG- waPUA and carbonyl groups of PAM chains firmly locking water molecules disrupting the formation of water crystal lattices.
PAAM	ZnSO_4_	1 M ZnSO_4_	5.56∙10^−3^	-	-	[161]	Zn/ PANI	Vertically conducting PANI nanowires deposited on the CNT film with high crystallinity degree and uniform orientation, provides high electrical conductivity and substantial mechanical strength
PAM	ZnSO_4_/MnSO_4_	2 M ZnSO_4_/ 0.1 M MnSO_4_	1.73∙10^−3^	RT	0.27	[162]	Zn-MnO_2_	MnSO_4_ suppresses the dissolution of Mn^2+^ from MnO_2_ into electrolyte stabilizing the MnO_2_ cathode. Eco-flex silicone endows a superior waterproof performance to the flexible battery. Double-helix carbon nanotube (CNT) yarns are used as substrates. Roll-dip-coating and roll-electrodeposition continuous processes were used to produce the MnO_2_ yarn cathode and zinc yarn anode continuously.
PAA	KOH	7.5 M	0.36	65	-	[163]	Zn-air	Solvodynamic radii of Zn(OH)_4_^2–^ calculated from the Stokes–Einstein equation had a range of values from 0.35 to 0.41 nm. For hydroxide ions, the thermochemical radius is 0.152 nm. Water retention is similar for PAA–KOH with 0.3, 0.5 and 0.7 mol% MBA. Higher crosslinking creates a denser network. When polymer structure then collapses, the network voids available for retaining water are minimized and water retention is decreased. Shape change on the Zn surface is also reduced with increasing MBA but ZAB cyclability is reduced.
PAA	KOH/ZnO	8 M KOH/ Saturated	55∙10^−3^	-	-	[164]	Zn-MnO_2_	Silver composite ink as current collector and nylon mesh embedded with electroactive inks show good flexibility avoiding cracks and delamination. The shear thinning behavior of the GPE can be used advantageously to allow printing with a lower pressure head
PANa	Zn(Ac)_2_/ KOH	0.2 M Zn(Ac)_2_/ 6 M KOH	0.17	-	≈0.15	[165]	Zn/NiCo Zn–air	Electrostatic interactions between the acrylate ions along the PANa backbone and Zn^2+^ facilitated the formation of quasi-SEI eliminating zinc dendrites confirmed by SEM and TEM images of the Zn anode. PANa exhibited a tunable electrochemical performance depending on the concentration of water, OH^−^ and/or Zn^2+^. The flexible quasi-SEI accommodated interface fluctuation during repeated charging/discharging without breakdown.
PANa	Zn(Ac)_2_/ KOH	0.2 M Zn(Ac)_2_/ 6 M KOH(sk.)	0.2	-	-	[166]	Zn/NiCo	Resistance of CNT papers coated with Au foil drops from 31.1 to 0.8 Ω. Facilitating uniform electrodeposition of nickel cobalt hydroxide and zinc. PANa hydrogel was first pre-stretched to over 400% strain
PAA	Zn(Ac)_2_/ ZnO	0.1–0.5 M Zn(Ac)_2_/ZnO	0.28	-	0.047	[167]	Zn-air	In aqueous electrolyte 6 M KOH, ZnO was found to be saturated at 0.5 M. The addition of 0.25 M ZnO reduces ionic conductivity of PAA–KOH. All the 0.25 M ZnO appears to be Zn(OH)_4_^2–^ in a highly alkaline solution (~6.5 M KOH). ZnO to reduce water activity and Zn corrosion.
P-(AM-co-AA)	Zn(Ac)_2_/ KOH	0.2 M Zn(Ac)_2_/ 6 M KOH (sk.)	0.148	-	0.052	[168]	Zn-air	(Fe_3_C@N-doped carbons) as bifunctional non-noble-metal electro- catalyst at the air electrode with highly ordered graphitized structure. Abundant carboxylic acid groups (PAA) hinder packing of polymer chains, endowing PAA with amorphous properties. Silicone encapsulation slows down losses of water locked by GPE.
PAAK	KOH	7.3 MKOH	0.6	25	-	[169]	Zn/Ni	The high solubility of Zn(OH)_4_^2−^ in alkaline solution results in the shape change of the zinc electrode, and the poor charge–discharge characteristics.
PANa	Zn(Ac)_2_/ KOH	0.2 M Zn(Ac)_2_/ 6 M KOH (sk.)	0.12	24	-	[170]	Zn/Ni	PANa hydrogel soaked by concentrated ions can be easily stretched to 1400% in both cold (−20 °C) and hot (50 °C) environments. Concentrated ions reduce the freezing point of the hydrogel. Different ionic conductivity at different temperatures is substantiated by their microstructure, at −20 °C fewer micropores are observed and the micropores are smaller
PEO(H_2_O)	KOH	30%	5–10∙10^−4^	RT	-	[171]	Zn/Ni, Zn/Cd	Conductivity log(s) decreases almost linearly with 1000/T temperature, an Arrhenius-type behavior, which is often observed for semi-crystalline polymer. The presence, at high O/K ratios, of diffraction peaks not observed in the PEO nor in the KOH, suggests the existence of a different crystalline entity
PEO/ PVA(H_2_O)	KOH	-	4–5∙10^−2^	RT	-	[172]	Zn-air	The surface morphology of film has a micro-porous structure consisting of many small pores with a dimension of about 0.1–0.2 µm. The cell with the solid polymer electrolyte has higher capacity and higher use of zinc on account of the much smaller pore size compared to PE/PP and cellulose separator.
PEO/PVdF	Zn(Ac)_2_/EMIMTFSI	15% Zn(Ac)_2/_7% EMIMTFSI	1.63∙10^−4^	RT	-	[173]	-	PEO (90 wt%)/PVdF (10 wt%)]—15 wt% Zn (CF_3_SO_3_)_2_ IL 7 wt% (i.e., best conducting sample) shows by SEM the presence of uniformly distributed spherulites with numerous dark boundaries. Conductivity dependence vs. temperature exhibit curved plots, which is strongly associate with the segmental motion of polymer chain
PEO/PVDF-HFP/EC/PC	Zn(Tf)_2_/ ZnO	1 M Zn(Tf)_2_/ 10%	3∙10^−3^	30	-	[174]	-	Raman shows that ZnO nanoparticles are present in the gelled polymer matrix as a separate phase. The temperature dependence σ vs. 1/T of the ionic conductivity of nanocomposite films showed VTF behavior. The gel electrolyte system EC–PC–Zn(Tf)_2_ immobilized in PVdF-HFP offers an acidic character.
PE/PVDF-HFP/EC/ PEGDME	Zn(Tf)_2_/ Zn(TFSI)_2_	0.5 M Zn(TFSI)_2_	4.7∙10^−4^	25	-	[175]	-	(PEGDMEs), methyl capped short-chain PEO possess excellent thermal and chemical stability. Blending PEGDME with a small amount of EC has large beneficial effects on the ionic conductivity. It is likely that a solid electrolyte interface (SEI) film forms at the surface of zinc, as in the case of lithium
PEO-PPO-PEO Pluronic Hydrogel (PHE)	ZnSO_4_/ Li_2_SO_4_	0.25 M ZnSO_4_/ 0.25 M Li_2_SO_4_	6.33∙10^−3^	25	-	[176]	Zn/LMO Zn/LFP	Perfect wetting of the electrodes, especially in the low temperature range. High initial open-circuit voltage (1.60 V) of the Zn/LMO cell, close to the thermodynamic voltage (1.71 V). Li^+^—intercalation/de-intercalation processes on the cathode side upon cycling. After crack due to bending or By a cooling-recovery procedure, PHE reversibly turned into its fluid phase rewetting the electrode in situ in 5 min.
Starch	KOH	6 M	4.34∙10^−3^	RT	-	[177]	-	-
Cellulose/PAA/ gelatin	KOH	0.4%	0.097	RT	-	[178]	-	Pristine NFC hydrogel had 10^−6^–10^−7^ S/cm^−1^. With KOH NFC hydrogel became brittle, and not stable. Adding PAA and gelatin, more stable GPEs are attained.
QA-functionalized nanocellulose/ GO	KOH	1 M (sk.)	58.8∙10^−3^	70	-	[179]	Zn-air	According to the XRD results and activation energies, two types of ion transport including Grotthuss mechanism and vehicle mechanism exist. The water uptake of the QAFCGO membrane and performance stability in a zinc-air battery is higher than those of the A201 membrane.
Cellulose nanofibres	ZnCl_2_/ NH_4_Cl	2 M ZnCl_2_/ 3 M NH_4_Cl	16.4∙10^−3^	-	-	[180]	Zn-ion	Graphite papers as flexible substrates for anodes. Superior battery performance related to the nanostructured PANI grown on lens paper. The nanostructured PANI facilitates electron and ion transport as well as its use during electrochemical process.
Functionalized DMOAP Cellulose nanofibres	KOH	1 M	21.2∙10^−3^	-	-	[181]	Zn-air	Superior hydroxide-ion conduction and water retention of the membrane as well as low anisotropic swelling. Improved cycling stability of the battery, compared to commercial alkaline AEM (A201). Self-purging of carbonates in zinc-air batteries in CO_2_ rich atmosphere.
BC/PVA	Zn(Ac)_2_/ KOH	6.0 M KOH/0.2 M Zn(Ac)_2_	80.8∙10^−3^	RT	0.951	[182]	Zn-air	High crystallinity, high purity, and high hygroscopicity of bacterial cellulose (BCs). Load-bearing percolating dual network, these BC/PVA composite membranes exhibit superior mechanical strength and toughness.
Gelatin/Borax	ZnSO_4_/ MnSO_4_	4∙10^−2^ mol ZnSO_4_ 4∙10^−3^ mol MnSO_4_	20∙10^−3^	-	-	[183]	Zn-ion	Shape memory wire battery Zn-ion battery (SMWB) using Nitinol (NT). Twined yarns of SS/MnO_2_/PANI and Nitinol/Zn. Gelatin/Borax GPE showed similar performance respect to the liquid one.
Gelatin	KOH	0.1 M	3.1∙10^−3^	-	-	[184]	Zn-air	Nonprecious metal catalyst (NPMC) based on silk fibroin for metal/nitrogen/carbon (M/N/C) with high catalytic activity for the ORR. Cable-type flexible ZAB with a spiral zinc anode.
κ-carrageenan/ rice paper	ZnSO_4_/ MnSO_4_	2 M ZnSO_4_/ 0.1 M MnSO_4_	33.2∙10^−3^	RT	-	[185]	Zn-ion	After 300 bending cycles, 95% capacity was retained.
PNiPAM/CMC	Zn(Tf)_2_	0–30%	0.17∙10^−3^	RT	35.6	[186]	Zn-ion	PNiPAM suppress dendrite formation. Most CMC/PNiPAM blend exhibited better mechanical properties than that of CMC. Thermo-responsive macromolecular transition from a hydrophilic to a hydrophobic structure at 30–35 °C
PVA/chitosan/ EC	NH_4_NO_3_	40%	1.6∙10^−3^	RT	-	[187]	Zn/H^+^ battery	Conductivity–temperature plot is Arrhenian type. Proton battery. ZnSO_4_ added to zinc anode.
Chitosan/PDDA/GA	KOH	2 M	24∙10^−3^	RT	25.30	[188]	Zn-air	Glutareldehide crooslinking. Good stability in 8 M KOH at 80 °C. Used in fuel cell, capacitor, and zinc-air battery.
Guar ammonium salt/GA/PCL	KOH	2 M	0.123		90	[189]	Zn-air	Low anisotropic swelling degree, outstanding mechanical strength, and excellent thermal stability. Binary cross-linking strategy to prepare highly conductive alkaline anion polymer electrolyte. Discharge time and capacity are superior to that the A201 membrane
Gelatin/ NaAlginate/GA (GAME)	ZnSO_4_	2 M ZnSO_4_	3.7∙10^−2^	2.14	2.14	[190]	Zn-ion	Good interfacial contact between electrodes and GAME. 3D cross-linked IPN network with rich functional groups provides good ionic conductivity. Elasticity and toughness for a better resistance to Zn dendrite attack.
Poly ε-caprolactone	Zn(Tf)_2_	25%	8.8∙10^−6^	25	-	[191]	Zn-MnO_2_	PCL (polycaprolactone) becomes a smoother one due to the addition of salt as observed from SEM. Good electrochemical stability window of 3.7 V with an excellent reversibility
PAN/PC/EC	Zn(Tf)_2_	≈0.6 M	2.7∙10^−3^	27	-	[192]	Zn-ion	The mass ratio of (PC-EC) to PAN is approximately 5:1. The logσ vs. 1/T relationship follows an Arrhenius-type behavior at all compositions.
Silica	Li_2_SO_4_/ ZnSO_4_	2 M Li_2_SO_4_/ 1 M ZnSO_4_	60∙10^−3^	RT	-	[193]	Zn/LiMn_2_O_4_	The required quantities of silica materials are in the range of 4–15 wt%. 10–12% higher cyclability compared with the performance of the batteries using the conventional liquid.
Fumed silica	ZnSO_4_	2 M ZnSO_4_	8.1∙10^−3^	RT	-	[194]	Zn-ion	Zn dendrite growth is thoroughly eliminated.
Gelatin/PAM	ZnSO_4_/ MnSO_4_	2 M ZnSO_4_/ 0.1 M MnSO_4_	1.76∙10^−2^	RT	7.76	[12]	Zn-ion	Flexible ZIB can be tailored to any desired shape to meet the demands of high-level integration. ZIB did not catch fire even after being exposed to fire for more than 5 min. Reliable power source that can work under a variety of severe conditions, such as being bent, hammered, punctured, cut, sewed, washed in water, and set on fire
Gelatin	ZnSO_4_/ MnSO_4_	2 M ZnSO_4_/ 0.1 M MnSO_4_	5.68∙10^−3^	RT	1.25	[12]		
Gelatin	ZnSO_4_/ Li_2_SO_4_	0.5 M ZnSO_4_/ 0.5 M Li_2_SO_4_	6.1∙10^−3^	-	0.11	[87]	Zn/LiMn_2_O_4_	In situ coating of the GHE on the electrodes. Solidifies into a strong film, provides mechanical strength to suppress Zn dendrite formation. Fast cooling rate leads to more orderly localized gelatin molecules and a more robust electrolyte
Xanthan gum	ZnSO_4_/ MnSO_4_	3 M ZnSO_4_/ 0.1 M MnSO_4_	16.5∙10^−3^ 2.5∙10^−3^	−8 °C	-	[64]	Zn-ion	A high-salt tolerant, water-soluble polysaccharide. Conductivity at −8 °C, suggesting its ability of working at low temperatures. Gum electrolyte has a long-term stability.

**Table 4:** Common popular biobased polymers used for making Zinc batteries GPEs-based.

Polymer/Average MW (kDa)	
Chitosan/100–800 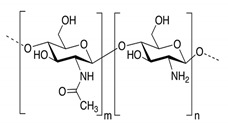	κ-Carrageenan/200–800 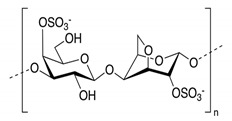
Carboxymethyl cellulose sodium salt/250 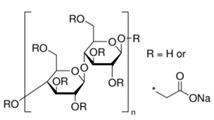	Sodium Alginate/900 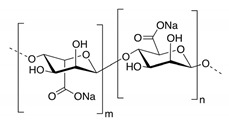
Gelatin/20–220 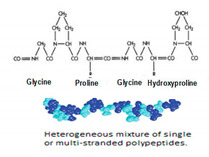	Cellulose/200–20,000 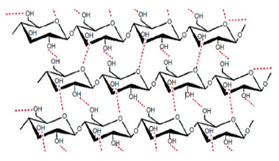
Lignine/600–180,000 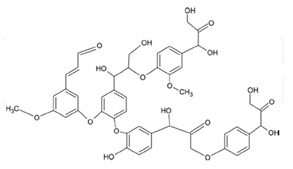	Xanthan gum/2000 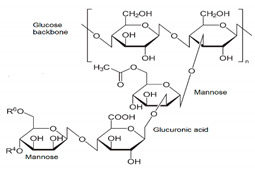
Guar gum/50–8000 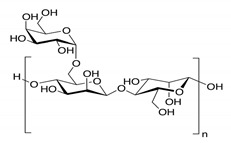	Agar-Agar/8–100 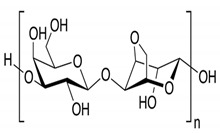
Starch/300 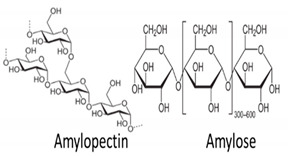	Methyl cellulose/10–220 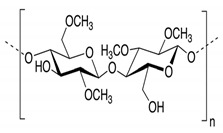
